# An efficient and concise access to 2-amino-4*H*-benzothiopyran-4-one derivatives

**DOI:** 10.3762/bjoc.15.65

**Published:** 2019-03-18

**Authors:** Peng Li, Yongqi Wu, Tingting Zhang, Chen Ma, Ziyun Lin, Gang Li, Haihong Huang

**Affiliations:** 1 State Key Laboratory of Bioactive Substance and Function of Natural Medicines & Beijing Key Laboratory of Active Substance Discovery and Druggability Evaluation, Institute of Materia Medica, Peking Union Medical College and Chinese Academy of Medical Sciences, 1 Xian Nong Tan Street, Beijing 100050, P. R. China

**Keywords:** 2-amino-4*H*-benzothiopyran-4-ones, addition–elimination, scale-up synthesis, sulfinyl group

## Abstract

A highly efficient and convenient protocol was developed to access 2-amino-4*H*-benzothiopyran-4-ones through a process of conjugated addition–elimination. The sulfinyl group was proved to be the optimum leaving group by thorough investigations on the elimination of sulfide, sulfinyl, and sulfonyl groups at the 2-position of benzothiopyranone. Most 2-aminobenzothiopyranones were obtained in good to excellent yields under refluxing in isopropanol within 36 h. This method is base-free and the substrate scope in terms of electronic properties of the substituents of the benzothiopyranone is broad. The ten grams scale-up synthesis of the representative compounds **4a** and **4d** was implemented to show the practical application of this reaction, which afforded the corresponding compounds in good yields and excellent chemical purity without requiring column chromatographical purification.

## Introduction

Benzothiopyranones are a class of molecules displaying biological activities in part due to their structural relationship with benzopyranones, which are known as one privileged scaffold in medicinal chemistry [1]. The corresponding 2-aminobenzothiopyranones are molecules of high interest as they have remarkable anticancer, antifungal and antitubercular activities [2–5].

**Scheme 1:**
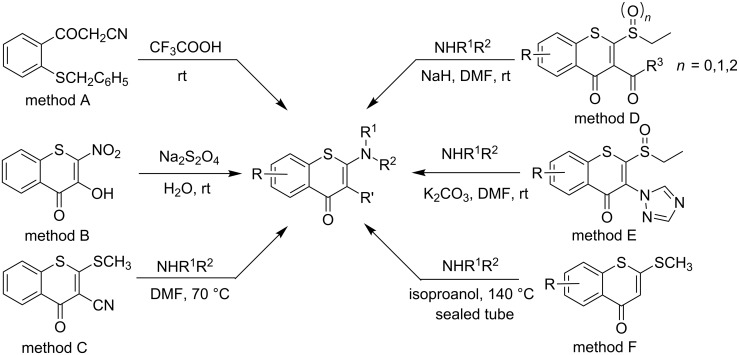
Representative strategies for the synthesis of *N*-substituted 2-aminobenzothiopyranones.

Compared to the versatile synthetic methods leading to 2-aminobenzopyranone derivatives, the approaches to 2-aminobenzothiopyranones are somewhat limited. Some simple 2-aminobenzothiopyranones have been obtained by the cyclization of 2-thiobenzoylacetonitrile treated with a strong acid (method A, Scheme 1) [6–7], or through the reduction of 2-nitrobenzothiopyranones with sodium dithionite (method B, Scheme 1) [8]. Apparently, synthetic methods to afford *N*-substituted 2-aminobenzothiopyranones are less well developed. The first example of an interchange of a 2-methylthio substituent for a nitrogen substituent leading to 2-methylamino-3-cyanobenzothiopyranones was disclosed by Rudorf and co-workers (method C, Scheme 1) [9]. Recently, a similar strategy was successfully utilized to afford *N*-substituted 2-aminobenzothiopyranones through the replacement of sulfide, sulfinyl or sulfonyl groups with amines in the presence of bases such as NaH, K_2_CO_3_, etc. However, for this reaction an adjacent electron-withdrawing group such as a carbonyl or triazole group is required at the 3-position of the thiochromone ring (method D and E, Scheme 1) [3–4]. Very recently, our group obtained a series of 2-aminobenzothiopyranones containing 8-nitro and 6-trifluoromethyl substituents in low to moderate yields through the transformation of a 2-methylthio substituent under harsh conditions (method F, Scheme 1) [5]. In addition, the nucleophile imidazole could also react with 3-bromobenzothiopyranones to afford 2-imidazolylbenzothiopyranones [10].

It is well known that the sulfide, sulfinyl and sulfonyl groups are generally used as the leaving group for the synthesis of 2-substituted 4*H-*chromen-4-ones [11–22]. Due to the higher electronegativity of the oxygen atom compared to the sulfur atom, the 2-position of the benzopyranone scaffold is easier to be attacked by nucleophiles than that the corresponding benzothiopyranone. We anticipate that the electron-withdrawing effect of a cyano, carbonyl, nitro or trifluoromethyl group, especially at the 3-position could render the sulfide, sulfinyl or sulfonyl moiety with higher reactivity at the 2-position of the benzothiopyranone ring.

In our continuing efforts to discover novel 2-amino-4*H*-benzothiopyran-4-ones with activity against *Mycobacterium tuberculosis* (*Mtb*) [5], we aim to develop an improved and efficient synthetic route by thoroughly evaluating the feasibility of the elimination of sulfide, sulfinyl or sulfonyl groups at the 2-position of the benzothiopyranone scaffold without requiring an adjacent electron-withdrawing substituent at the 3-position.

## Results and Discussion

**Scheme 2:**
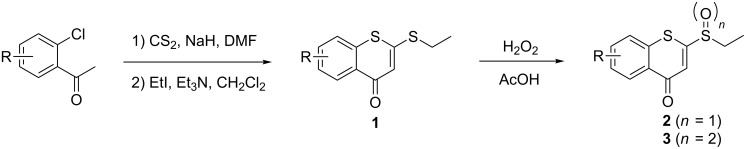
The synthesis of sulfide **1**, sulfoxide **2**, and sulfone **3**.

Firstly, 2-ethylthio-4*H-*thiochromen-4-one **1** was readily prepared from 2’-chloroacetophenone as the starting material through treatment with carbon disulfide in the presence of sodium hydride [23], followed by alkylation using iodoethane according to the literature procedures [13,16–17,19]. The oxidation of **1** with 1.2 or 5 equiv of H_2_O_2_ yielded ethyl sulfoxide **2** and ethyl sulfone **3**, respectively (Scheme 2).

With the substrates including sulfide, sulfoxide and sulfone in hand, we screened various solvents with compounds **1a**, **2a**, **3a** and 1-benzylpiperazine as the model substrates and the results are summarized in Table 1. No target compound **4a** was obtained at room temperature in all solvents with **1a** bearing an ethylthio group as the starting material according to the method of benzopyranone preparation [13–14,17]. Even under refluxing conditions, substrate **1a** could not provide the target compound **4a** (Table 1, entries 1, 4, 7, 10 and 13), suggesting that the replacement of an ethylthio group requires even more drastic conditions [21]. To our delight, under refluxing conditions, the substrates with sulfinyl and sulfonyl groups led to **4a** in THF and dioxane, however, in low to moderate yields (Table 1, entries 5, 6, 8 and 9). Furthermore, reaction of the substrate **2a** having a sulfinyl group in ethanol or isopropanol afforded the target compound in good to excellent yield from 75% to 90% (Table 1, entries 11 and 14), indicating that a polar protic solvent promotes the reaction. When the reactions were performed with 1.2 equiv of 1-benzylpiperazine with or without Et_3_N in isopropanol under refluxing conditions, the yields decreased from 90% to 26% and 30%, respectively, due to considerable amounts of unreacted substrate **2a** (Table 1, entries 14, 16 and 17). Although the sulfonyl group has been used more frequently due to its higher leaving ability reactivity toward nucleophiles, we found that substrate **3a** with sulfonyl group easily decomposed at high temperature and led to low yields (Table 1, entries 9, 12 and 15 vs entries 8, 11 and 14). Therefore, we were encouraged to confirm the consistent superiority of ethylsulfinyl over ethylsulfonyl as a leaving group to afford the desired 2-amino-4*H*-benzothiopyran-4-ones through conjugated addition–elimination by nucleophiles.

**Table 1:** Optimization of the reaction conditions.^a^

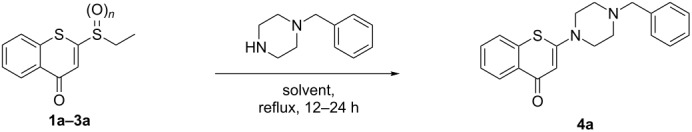				

Entry	*n*	Solvent	Time (h)	Yield^b^ (%)

1	0	acetonitrile	24	–
2	1	acetonitrile	24	trace
3	2	acetonitrile	24	trace
4	0	THF	24	–
5	1	THF	24	21
6	2	THF	24	18
7	0	dioxane	12	–
8	1	dioxane	12	58
9	2	dioxane	12	51
10	0	ethanol	24	–
11	1	ethanol	24	75
12	2	ethanol	24	52
13	0	isopropanol	12	–
14	1	isopropanol	12	90
15	2	isopropanol	12	37
16^c^	1	isopropanol	12	30
17^d^	1	isopropanol	12	26

^a^The reactions (entries 1–15) were performed with 1.0 mmol of **1a–3a** and 2.0 mmol of 1-benzylpiperazine in 5 mL of the indicated solvent under refluxing conditions. ^b^Isolated yields. ^c^The reaction was performed with 1.0 mmol of **2a** and 1.2 mmol of 1-benzylpiperazine in 5 mL of isopropanol under refluxing conditions. ^d^The reaction was performed with 1.0 mmol of **2a**,1.2 mmol of 1-benzylpiperazine and 1.2 mmol Et_3_N in 5 mL of isopropanol under refluxing conditions.

Subsequently, substrates **2a** and **3a** were reacted with a variety of alkyl/arylamines in the optimized solvent isopropanol (Table 2). Substrate **2a** having an ethylsulfinyl group in general provided a much higher product yield (Table 2, entries 1, 3 and 5 vs entries 2, 4 and 6, respectively) and sometimes required a shorter reaction time (Table 2, entry 3 vs entry 4) than the corresponding substrate **3a**. To further verify the advantage of the sulfinyl group as the optimal leaving group, the representative amines including primary and secondary amines were then investigated in reactions with substrate **2a**. We were pleased to find that various *N*-substituted 2-aminobenzothiopyranones **4e–i** were afforded in moderate to good yields. In general, aliphatic amines NHR^1^R^2^ are stronger nucleophiles to furnish the desired products in high yields in a relatively short reaction time compared to aromatic amines, exemplified by entries 1, 7 vs entries 3, 5, 8–10 in Table 2. Both primary and secondary aliphatic amines could smoothly afford the corresponding products (Table 2, entries 3, 5 and 8–11).

**Table 2:** Further confirmation of the sulfinyl/sulfonyl groups as optimal leaving groups.^a^

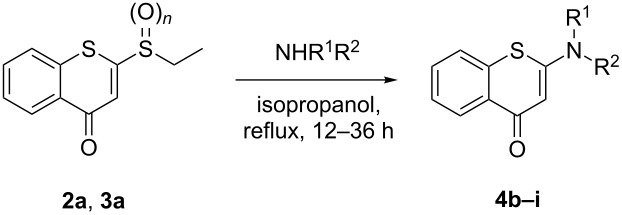					

Entry	*n*	Product	NHR^1^R^2^	Time (h)	Yield^b^ (%)

1	1	**4b**	aniline	36	71
2	2	**4b**	aniline	36	50
3	1	**4c**	benzylamine	12	75
4	2	**4c**	benzylamine	36	52
5	1	**4d**	propylamine	12	78
6	2	**4d**	propylamine	12	64
7	1	**4e**	*p*-methoxyaniline	36	55
8	1	**4f**	3,4-dimethoxybenzylamine	12	79
9	1	**4g**	cyclohexylmethylamine	12	76
10	1	**4h**	cyclohexylamine	24	71
11	1	**4i**	diethylamine	24	67

^a^All reactions were performed with 1.0 mmol of **2a**/**3a** and 2.0 mmol of NHR^1^R^2^ in 5 mL of isopropanol. ^b^Isolated yields.

**Scheme 3:**
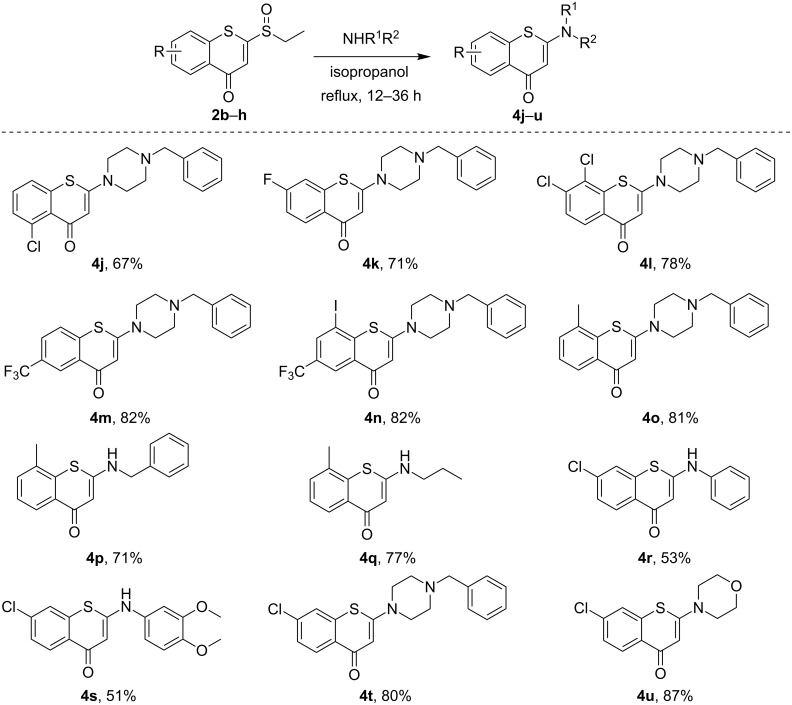
Scope of the synthesis of versatile 2-aminobenzothiopyranones. All reactions were performed with 1.0 mmol of **2b–h** and 2.0 mmol of NHR^1^R^2^ in 5 mL of isopropanol under refluxing conditions and yields refer to isolated yields.

Having identified the optimal conditions, we next explored the scope of the reaction. Various sulfinyl substrates **2b–h** with electron-donating or electron-withdrawing substituents in the benzothiopyranone scaffold were treated with 1-benzylpiperazine as well as other representative amines (Scheme 3). Due to the strong nucleophilicity of piperazine and morpholine, the substrates with electron-donating/withdrawing and halogen groups smoothly delivered the *N*-substituted 2-aminobenzothiopyranones **4j**–**o** and **4t**,**u** in good to excellent yields. Specifically, the substrates containing a strong electron-withdrawing group such as trifluoromethyl gave the desired products, e.g., **4m**,**n** in high yields. Substrates bearing electron-donating groups also reacted with aliphatic amines giving products **4o**–**q** in good yields. It was noted that the treatment of substrate **2** containing a chloro substituent with weaker nucleophilic amines such as arylamines successfully afforded the targeted compounds **4r**,**s** albeit in only about 50% yield. The results indicated that the nucleophilicity of the amine exerts a more pronounced effect on the reactivity (i.e., aliphatic amines vs aromatic amines) under the above optimal conditions established.

**Scheme 4:**
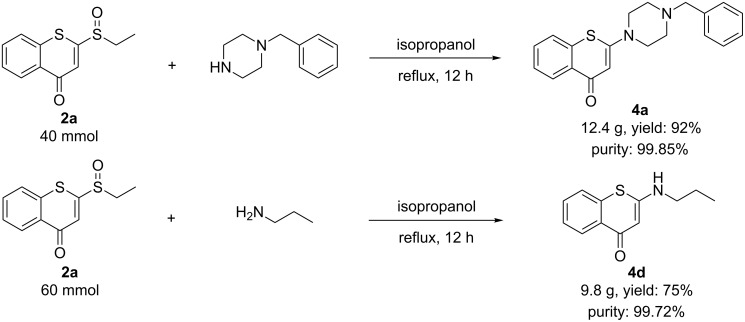
The gram-scale synthesis of 2-aminobenzothiopyranones **4a** and **4d**.

To further evaluate the efficiency of this methodology, a gram-scale synthesis of the representative compounds **4a** and **4d** was performed. As depicted in Scheme 4, by treatment of 40 mmol of **2a** with 1-benzylpiperazine under the optimized conditions, the product **4a** was obtained in an excellent yield (92%, 12.4 g) with >99% chemical purity. Following the same procedure starting from **2b** gave product **4d** in good yield (75%, 9.8 g) as well as with >99% chemical purity. To our delight, both products **4a** and **4d** were successfully isolated by directly filtration of the reaction mixture instead of column chromatographical purification that was employed in the milligram-scale synthesis. Overall, this synthetic route is efficient and applicable to scale-up.

## Conclusion

In summary, we have developed a facile and efficient strategy for the synthesis of 2-aminobenzothiopyranones through a conjugated addition–elimination process. The direct C–N bond formation reaction at the 2-position smoothly took place using ethylsulfinyl as the optimal leaving group and various nucleophiles such as aliphatic and aromatic amines. A variety of 2-aminobenzothiopyranones were obtained in moderate to excellent yields without the assistance of an adjacent electron-withdrawing group or additional base. This practical method could be scaled-up to a ten-gram scale allowing isolation of the products by simple filtration. This synthetic route is complementary to the existing methods for the synthesis of 2-aminobenzothiopyranone derivatives, thereby providing opportunities for discovering more active compounds for medicinal chemistry research.

## Supporting Information

File 1 General methods, general procedures, characterization data and copies of ^1^H and ^13^C NMR spectra of **1a–h**, **2a–h**, **3a** and **4a–u**.
