# Supplementary material for: An efficient and concise access to 2-amino-4H-benzothiopyran-4-one derivatives
Source: Beilstein J Org Chem. 2019 Mar 18;15:703–9. doi: 10.3762/bjoc.15.65 (PMC6444430; doi:10.3762/bjoc.15.65)

BEILSTEIN JOURNAL OF ORGANIC CHEMISTRY

Supporting Information

for

An efficient and concise access to 2-amino-4*H*-benzothiopyran-4-one derivatives

Peng Li, Yongqi Wu, Tingting Zhang, Chen Ma, Ziyun Lin, Gang Li and Haihong Huang

Beilstein J. Org. Chem. **2019,** *15,* 703–709. doi:10.3762/bjoc.15.65

General methods, general procedures, characterization data and copies of ^{1}H and ^{13}C NMR spectra of 1a–h, 2a–h, 3a and 4a–u

License and Terms: This is a supporting information file under the terms of the Creative Commons Attribution License (http://creativecommons.org/licenses/by/4.0). Please note that the reuse, redistribution and reproduction in particular requires that the authors and source are credited.
The license is subject to the *Beilstein Journal of Organic Chemistry* terms and conditions: (https://www.beilstein-journals.org/bjoc)

Table of contents

General methods......S1

General procedures......S1

Characterization data for the products......S3

Copies of ^{1}H and ^{13}C NMR spectra......S12

General methods

Reactions were monitored by thin layer silica gel chromatography (TLC) using GF254 or exposure to I_2. Melting points were determined on Yanaco MP-*J*3 microscope melting point apparatus. ^{1}H and ^{13}C NMR spectra were recorded on Varian 400 MHz NMR spectrometer with $CDCl_3$ or DMSO-d_6 as the solvent. Chemical shifts are referenced to the residual solvent peak and reported in ppm (δ scale) and all coupling constant (*J*) values are given in Hz. The following multiplicity abbreviations are used: (s) singlet, (d) doublet, (t) triplet, (q) quartet, (m) multiplet, and (brs) broad singlet. ESI-HRMS data were measured on Thermo Exactive Orbitrap plus spectrometer. All solvents and reagents were obtained from commercial suppliers and used without further purification.

The purity of **4a** and **4d** was measured by Shimadzu LC 20 with UV detector SPD-20A HPLC equipment with Kromasil ODS column. Column size: 250 mm × 4.6 mm, 5 µm. Mobile phase: 10 mM NH_4Ac (A) and methanol (B). The elution procedure: 0–5 min, B, 45%; 5–10 min, B, 45–85 %; 10–35min, B, 85%; 35–40 min, B, 85–45 %; 40–60 min, B, 45%. Detection wavelength: 235 nm. Column temperature: 30 °C. Injection volume: 25 µL. Flow rate: 1.0 mL/min.

General procedures

General procedure A for the preparation of sulfide 1a, sulfoxide 2a, and sulfone 3a.

Sodium hydride (1.20 g, 30 mmol, *c* 60% in oil) was added in portions to a stirred mixture of 2'-chloroacetophenone (2.32 g, 15 mmol), *N,N*-dimethylformamide (15 mL), toluene (10 mL) and carbon disulfide (2.28 g, 30 mmol) keeping the reaction temperature between 15–20 °C. After additional 30 min, methanol (0.4 mL) was carefully added and stirring continued for 30 min. After evaporation of toluene under reduced pressure, the residue was heated until the internal temperature reached 125–130 °C, maintained at 125–130 °C for 30 min, cooled and diluted with water (50 mL). After the addition of acetic acid (1.5 mL) and extraction with ether (2 × 50 mL), the aqueous phase was separated, filtered and acidified with conc. HCl to

pH 1. The precipitate was filtrated, washed with water, and dried to give 1.94 g of a yellow solid which was used in the next step without purification.

To a magnetically stirred solution of the above obtained solid (1.94 g, 10 mmol) and Et_3N (2.00 g, 20 mmol) in CH_2Cl_2 (50 mL) was added iodoethane (1.55 g, 10 mmol). The reaction mixture was stirred for 1 h at 35 °C. After cooling to room temperature, the reaction mixture was poured into 1 N HCl (50 mL). The organic phase was washed with H_2O (50 mL), brine (50 mL), and then evaporated under reduced pressure. The residue was purified by column chromatography (EtOAc/PE 1:4) to give the title compound **1a** (2.12 g, 64% over two steps).

To a magnetically stirred solution of **1a** (1.11 g, 5 mmol) in AcOH (5 mL) was added 1 M H_2O_2/AcOH (6 mL, 1.2 equiv) and the reaction mixture was stirred at 60 °C overnight. The reaction solution was poured into water (25 mL) and extracted with dichloromethane (2 × 25 mL). The organic extract was washed with water (50 mL), saturated $NaHCO_3$ (25 mL), brine (25 mL), dried over Na_2SO_4 and then evaporated. The crude material was purified by recrystallization (hexane/EtOAc 5:2) to give the title compound **2a** (1.05 g, 88%). The corresponding compounds **1b–h** and **2b–h** were prepared according to the general procedure A. The title compound **3a** was prepared using 5.0 equiv hydrogen peroxide following general procedure A.

General procedure B for the preparation of 2-(4-benzylpiperazin-1-yl)-4*H*-thiochromen-4-one (4a).

To a magnetically stirred solution of **2a** (238 mg, 1 mmol) in isopropanol (5 mL) was added 1-benzylpiperazine (353 mg, 2 mmol) and the reaction mixture was heated to reflux for 12 h. After cooling to room temperature, the reaction mixture was concentrated in vacuum, and the residue was purified by column chromatography (MeOH/CH_2Cl_2 1:100) to give the target compound **4a** (303 mg, 90%). The target compounds **4b–u** were prepared according to the general procedure B.

General procedure C for the scale-up preparation of 4a and 4d.

To a magnetically stirred solution of **2a** (9.53 g, 40 mmol) in isopropanol (70 mL) was added 1-benzylpiperazine (14.10 g, 80 mmol). The reaction mixture was heated to reflux for 12 h. After cooling to room temperature, the reaction mixture was left standing overnight, filtrated and the obtained solid was washed with 20 mL ethanol to give the target compound **4a**

(12.4 g, 92%). The target compound **4d** was prepared according to the general procedure C with **2a** (14.30 g, 60 mmol) and propylamine (7.09 g 120 mmol) in good yield (9.8 g, 75%).

Characterization data for the products

2-(Ethylthio)-4*H*-thiochromen-4-one (1a): Off-white solid; yield: 2.12 g (64%); mp 65-66 °C. ^{1}H NMR (400 MHz, $CDCl_3$) δ = 8.46 (d, *J* = 6.8 Hz, 1H), 7.58-7.55 (m, 1H), 7.51-7.49 (m, 2H), 6.96 (s, 1H), 3.13 (q, *J* = 6.0 Hz, 2H), 1.43 (t, *J* = 6.0 Hz, 3H). ^{13}C NMR (100 MHz, $CDCl_3$) δ = 178.6, 154.0, 137.7, 131.5, 130.7, 128.7, 127.7, 125.4, 122.7, 27.8, 14.0. HRMS (ESI): m/z $[M+H]^+$ calcd for $C_{11}H_{11}OS_2$, 223.0246; found, 223.0247.

5-Chloro-2-(ethylthio)-4*H*-thiochromen-4-one (1b): Off-white solid; yield: 3.15 g (82%); mp 87-89 °C. ^{1}H NMR (400 MHz, $CDCl_3$) δ = 7.49-7.45 (m, 1H), 7.41-7.39 (m, 2H), 6.86 (s, 1H), 3.10 (q, *J* = 7.6 Hz, 2H), 1.42 (t, *J* = 7.2 Hz, 3H). ^{13}C NMR (100 MHz, $CDCl_3$) δ = 178.2, 150.6, 140.6, 136.2, 131.5, 130.8, 127.6, 124.5, 124.3, 27.8, 14.0. HRMS (ESI): m/z $[M+H]^+$ calcd for $C_{11}H_{10}ClOS_2$, 256.9856; found, 256.9857.

2-(Ethylthio)-7-fluoro-4*H*-thiochromen-4-one (1c): Off-white solid; yield: 1.26 g (35%); mp 86-88 °C. ^{1}H NMR (400 MHz, $CDCl_3$) δ = 8.50-8.47 (m, 1H), 7.23-7.19 (m, 2H), 6.93 (s, 1H), 3.13 (q, *J* = 7.2 Hz, 2H), 1.43 (t, *J* = 7.6 Hz, 3H). ^{13}C NMR (100 MHz, $CDCl_3$) δ = 177.7, 164.0 (d, *J* = 255 Hz), 153.7, 139.8 (d, *J* = 10 Hz), 131.8 (d, *J* = 10 Hz), 127.4, 122.8, 116.2 (d, *J* = 22 Hz), 111.5 (d, *J* = 25 Hz), 27.9, 14.0. HRMS (ESI): m/z $[M+H]^+$ calcd for $C_{11}H_{10}FOS_2$, 241.0152; found, 241.0151.

7,8-Dichloro-2-(ethylthio)-4*H*-thiochromen-4-one (1d): Yellow solid; yield: 2.05 g (47%); mp 123-125 °C. ^{1}H NMR (400 MHz, $CDCl_3$) δ = 8.34 (d, *J* = 6.8 Hz, 1H), 7.59 (d, *J* = 6.8 Hz, 1H), 6.94 (s, 1H), 3.17 (q, *J* = 6.0 Hz, 2H), 1.45 (t, *J* = 6.0 Hz, 3H). ^{13}C NMR (100 MHz, $CDCl_3$) δ = 177.8, 154.8, 138.8, 137.0, 130.9, 128.8, 128.0, 127.8, 122.0, 28.0, 13.9. HRMS (ESI): m/z $[M+H]^+$ calcd for $C_{11}H_9Cl_2OS_2$, 290.9466; found, 290.9465.

2-(Ethylthio)-6-(trifluoromethyl)-4*H*-thiochromen-4-one (1e): Off-white solid; yield: 2.40 g (55%); mp 90-92 °C. ^{1}H NMR (400 MHz, $CDCl_3$) δ = 8.74 (s, 1H), 7.78 (d, *J* = 6.8 Hz,

1H), 7.63 (d, *J* = 6.8 Hz, 1H), 6.97 (s, 1H), 3.16 (q, *J* = 6.0 Hz, 2H), 1.45 (t, *J* = 6.0 Hz, 3H). ^{13}C NMR (100 MHz, $CDCl_3$) δ = 177.5, 154.8, 141.2, 130.9, 130.1 (q, $^{2}J_{F, C}$ = 33 Hz), 127.5, 126.3, 126.2, 123.5 (q, $^{1}J_{F, C}$ = 271 Hz), 122.4, 27.8, 13.9. HRMS (ESI): m/z $[M+H]^+$ calcd for $C_{12}H_{10}F_3OS_2$, 291.0120; found, 291.0120.

2-(Ethylthio)-8-iodo-6-(trifluoromethyl)-4*H*-thiochromen-4-one (1f): Yellow solid; yield: 2.87 g (46%); mp 147-149 °C. ^{1}H NMR (400 MHz, $CDCl_3$) δ = 8.76 (s, 1H), 8.23 (d, *J* = 1.6 Hz, 1H), 6.94 (d, *J* = 2.0 Hz, 1H), 3.19 (q, *J* = 7.2 Hz, 2H), 1.46 (t, *J* = 7.6 Hz, 3H). ^{13}C NMR (100 MHz, $CDCl_3$) δ = 178.0, 156.1, 145.5, 138.0, 132.9, 130.8 (q, $^{2}J_{F, C}$ = 34 Hz), 126.1, 122.6 (q, $^{1}J_{F, C}$ = 272 Hz), 121.1, 94.2, 27.9, 13.8. HRMS (ESI): m/z $[M+H]^+$ calcd for $C_{12}H_9F_3IOS_2$, 416.9086; found, 416.9082.

2-(Ethylthio)-8-methyl-4*H*-thiochromen-4-one (1g): Off-white solid; yield: 2.34 g (66%); mp 93-95 °C. ^{1}H NMR (400 MHz, $CDCl_3$) δ = 8.36 (d, *J* = 5.6 Hz, 1H), 7.43-7.40 (m, 2H), 6.99 (s, 1H), 3.16 (q, *J* = 5.6 Hz, 2H), 2.50 (s, 3H), 1.44 (t, *J* = 5.6 Hz, 3H). ^{13}C NMR (100 MHz, $CDCl_3$) δ = 179.2, 153.4, 137.2, 133.8, 132.7, 131.0, 127.0, 126.5, 122.5, 27.9, 19.4, 14.0. HRMS (ESI): m/z $[M+H]^+$ calcd for $C_{12}H_{13}OS_2$, 237.0402; found, 237.0402.

7-Chloro-2-(ethylthio)-4*H*-thiochromen-4-one (1h): Off-white solid; yield: 2.50 g (65%); mp 120-121 °C. ^{1}H NMR (400 MHz, $CDCl_3$) δ = 8.38 (d, *J* = 8.8 Hz, 1H), 7.49 (s, 1H), 7.46-7.43 (m, 1H), 6.92 (s, 1H), 3.13 (q, *J* = 7.2 Hz, 2H), 1.43 (t, *J* = 7.6 Hz, 3H). ^{13}C NMR (100 MHz, $CDCl_3$) δ = 177.8, 153.9, 139.0, 138.2, 130.3, 129.1, 128.4, 124.7, 122.7, 27.9, 12.9. HRMS (ESI): m/z $[M+H]^+$ calcd for $C_{11}H_{10}ClOS_2$, 256.9856; found, 256.9858.

2-(Ethylsulfinyl)-4*H*-thiochromen-4-one (2a): Off-white solid; yield: 1.05 g (88%); mp 96-97 °C. ^{1}H NMR (400 MHz, $CDCl_3$) δ = 8.53 (d, *J* = 8.0 Hz, 1H), 7.69-7.67 (m, 2H), 7.63-7.59 (m, 1H), 7.25 (s, 1H), 3.21-3.12 (m, 1H), 3.06-2.97 (m, 1H), 1.37 (t, *J* = 7.2 Hz, 3H).^{13}C NMR (100 MHz, $CDCl_3$) δ = 178.6, 160.1, 136.1, 132.3, 131.5, 129.1, 128.6, 127.3, 122.9, 50.2, 5.8. HRMS (ESI): m/z $[M+H]^+$ calcd for $C_{11}H_{11}O_2S_2$, 239.0195; found, 239.0192.

5-Chloro-2-(ethylsulfinyl)-4*H*-thiochromen-4-one (2b): Off-white solid; yield: 0.97 g (71%); mp 120-122 °C. ^{1}H NMR (400 MHz, $CDCl_3$) δ = 7.61-7.49 (m, 3H), 7.16 (s, 1H), 3.18-3.13 (m, 1H), 3.03-2.98 (m, 1H), 1.37 (t, J = 7.2 Hz, 3H). ^{13}C NMR (100 MHz, $CDCl_3$) δ = 178.0, 156.9, 139.0, 136.6, 132.3, 131.6, 128.1, 126.4, 124.5, 49.9, 5.8. HRMS (ESI): m/z $[M+H]^+$ calcd for $C_{11}H_{10}ClO_2S_2$, 272.9805; found, 272.9804.

2-(Ethylsulfinyl)-7-fluoro-4*H*-thiochromen-4-one (2c): Off-white solid; yield: 0.90 g (70%); mp 115-117 °C. ^{1}H NMR (400 MHz, $CDCl_3$) δ = 8.57-8.53 (m, 1H), 7.40-7.37 (m, 1H), 7.34-7.29 (m, 1H), 7.22 (s, 1H), 3.20-3.12 (m, 1H), 3.07-2.98 (m, 1H), 1.38 (t, J = 7.6 Hz, 3H). ^{13}C NMR (100 MHz, $CDCl_3$) δ = 177.6, 164.3 (d, J = 257 Hz), 159.9, 138.3 (d, J = 10 Hz), 132.2 (d, J = 10 Hz), 128.1, 123.1, 117.3 (d, J = 22 Hz), 113.3 (d, J = 25 Hz), 50.3, 5.8. HRMS (ESI): m/z $[M+H]^+$ calcd for $C_{11}H_{10}FO_2S_2$, 257.0101; found, 257.0100.

7,8-Dichloro-2-(ethylsulfinyl)-4*H*-thiochromen-4-one (2d): Yellow solid; yield: 0.83 g (54%); mp 160-162 °C. ^{1}H NMR (400 MHz, $CDCl_3$) δ = 8.41 (d, J = 6.8 Hz, 1H), 7.68 (d, J = 6.8 Hz, 1H), 7.28 (s, 1H), 3.22-3.18 (m, 1H), 3.06-3.02 (m, 1H), 1.38 (t, J = 6.0 Hz, 3H). ^{13}C NMR (100 MHz, $CDCl_3$) δ = 177.9, 160.4, 138.0, 137.6, 131.6, 129.7, 128.4, 128.3, 122.6, 50.1, 5.8. HRMS (ESI): m/z $[M+H]^+$ calcd for $C_{11}H_9Cl_2O_2S_2$, 306.9416; found, 306.9416.

2-(Ethylsulfinyl)-6-(trifluoromethyl)-4*H*-thiochromen-4-one (2e): Off-white solid; yield: 1.30 g (85%); mp 126-128 °C. ^{1}H NMR (400 MHz, $CDCl_3$) δ = 8.80 (s, 1H), 7.90-7.87 (m, 1H), 7.83-7.81 (m, 1H), 7.28 (s, 1H), 3.21-3.15 (m, 1H), 3.06-3.00 (m, 1H), 1.39 (t, J = 7.2 Hz, 3H). ^{13}C NMR (100 MHz, $CDCl_3$) δ = 177.6, 160.9, 139.7, 131.6, 131.0 (q, $^{2}J_{F,C}$ = 34 Hz), 128.3, 128.2, 126.6, 123.3 (q, $^{1}J_{F,C}$ = 271 Hz), 123.2, 50.4, 5.8. HRMS (ESI): m/z $[M+H]^+$ calcd for $C_{12}H_{10}F_3O_2S_2$, 307.0069; found, 307.0065.

2-(Ethylsulfinyl)-8-iodo-6-(trifluoromethyl)-4*H*-thiochromen-4-one (2f): Off-white solid; yield: 1.84 g (85%); mp 146-148 °C. ^{1}H NMR (400 MHz, $CDCl_3$) δ = 8.82-8.81 (m, 1H), 8.34-8.33 (m, 1H), 7.31 (s, 1H), 3.24-3.19 (m, 1H), 3.08-3.04 (m, 1H), 1.40 (t, J = 7.2 Hz, 3H). ^{13}C NMR (100 MHz, $CDCl_3$) δ = 178.2, 161.6, 144.5, 138.8, 133.7, 131.6 (q, $^{2}J_{F,C}$ = 34 Hz), 126.5, 122.3 (q, $^{1}J_{F,C}$ = 272 Hz), 122.2, 96.6, 50.2, 5.9. HRMS (ESI): m/z $[M+H]^+$ calcd for $C_{12}H_9F_3IO_2S_2$, 432.9035; found, 432.9032.

2-(Ethylsulfinyl)-8-methyl-4*H*-thiochromen-4-one (2g): Off-white solid; yield: 1.06 g (84%); mp 106-108 °C. ^{1}H NMR (400 MHz, $CDCl_3$) δ = 8.42-8.40 (m, 1H), 7.55-7.49 (m, 2H), 7.28 (s, 1H), 3.19-3.16 (m, 1H), 3.08-3.03 (m, 1H), 2.58 (s, 3H), 1.37 (t, *J* = 7.2 Hz, 3H). ^{13}C NMR (100 MHz, $CDCl_3$) δ = 179.1, 159.0, 135.7, 135.4, 133.4, 131.9, 127.9, 126.9, 122.8, 50.2, 19.7, 5.9. HRMS (ESI): m/z $[M+H]^+$ calcd for $C_{12}H_{13}O_2S_2$, 253.0352; found, 253.0349.

7-Chloro-2-(ethylsulfinyl)-4*H*-thiochromen-4-one (2h): Off-white solid; yield: 1.15 g (84%); mp 117-118 °C. ^{1}H NMR (400 MHz, $CDCl_3$) δ = 8.45 (d, *J* = 8.4 Hz, 1H), 7.67 (s, 1H), 7.56-7.54 (m, 1H), 7.23 (s, 1H), 3.21-3.12 (m, 1H), 3.06-2.97 (m, 1H), 1.37 (t, *J* = 7.2 Hz, 3H). ^{13}C NMR (100 MHz, $CDCl_3$) δ = 177.8, 160.1, 139.1, 137.4, 130.6, 129.8, 129.3, 126.6, 123.1, 50.3, 5.9. HRMS (ESI): m/z $[M+H]^+$ calcd for $C_{11}H_{10}ClO_2S_2$, 272.9805; found, 272.9803.

2-(Ethylsulfonyl)-4*H*-thiochromen-4-one (3a): Off-white solid; yield: 1.08 g (85%); mp 99-100 °C. ^{1}H NMR (400 MHz, $CDCl_3$) δ = 8.53 (d, *J* = 8.0 Hz, 1H), 7.74-7.71 (m, 2H), 7.67-7.63 (m, 1H), 7.59 (s, 1H), 3.30 (q, *J* = 7.2 Hz, 2H), 1.43 (t, *J* = 7.2 Hz, 3H). ^{13}C NMR (100 MHz, $CDCl_3$) δ = 179.7, 150.7, 135.9, 133.0, 130.9, 129.1, 128.9, 127.4, 50.4, 7.4. HRMS (ESI): m/z $[M+H]^+$ calcd for $C_{11}H_{11}O_3S_2$, 255.0144; found, 255.0143.

2-(4-Benzylpiperazin-1-yl)-4*H*-thiochromen-4-one (4a): Yellow solid; yield: 303 mg (90%); mp 124-126 °C. ^{1}H NMR (400 MHz, $CDCl_3$) δ = 8.44-8.42 (m, 1H), 7.52-7.42 (m, 3H), 7.35-7.34 (m, 4H), 7.31-7.29 (m, 1H), 6.20 (s, 1H), 3.57-3.55 (m, 6H), 2.59 (brs, 4H). ^{13}C NMR (100 MHz, $CDCl_3$) δ = 180.4, 159.5, 137.3, 133.2, 130.7, 130.0, 129.1, 128.4, 128.1, 127.4, 127.1, 125.6, 101.2, 62.7, 52.2, 47.6. HRMS (ESI): m/z $[M+H]^+$ calcd for $C_{20}H_{21}N_2OS$, 337.1369; found, 337.1365.

2-(Phenylamino)-4*H*-thiochromen-4-one (4b): Off-white solid; yield: 180 mg (71%); mp 207-209 °C. ^{1}H NMR (400 MHz, DMSO-d_6) δ = 9.79 (brs, 1H), 8.23 (d, *J* = 6.8 Hz, 1H), 7.71 (d, *J* = 7.6 Hz, 1H), 7.60 (t, *J* = 7.2 Hz, 1H), 7.51-7.44 (m, 3H), 7.34 (d, *J* = 7.6 Hz, 2H), 7.25 (t, *J* = 7.2 Hz, 1H), 6.22 (s, 1H). ^{13}C NMR (100 MHz, DMSO-d_6) δ = 178.8, 156.8, 138.8,

133.3, 131.6, 130.5, 130.1, 127.6, 127.5, 126.8, 126.0, 124.0, 100.9. HRMS (ESI): m/z $[M+H]^+$ calcd for $C_{15}H_{12}NOS$, 254.0634; found, 254.0633.

2-(Benzylamino)-4*H*-thiochromen-4-one (4c): Yellow solid; yield: 200 mg (75%); mp 157-159 °C. ^1H NMR (400 MHz, $CDCl_3$) δ = 8.43 (dd, *J* = 7.2, 1.6 Hz, 1H), 7.50-7.31 (m, 8H), 6.14 (s, 1H), 5.45 (brs, 1H), 4.46 (d, *J* = 5.2 Hz, 2H). ^{13}C NMR (100 MHz, $CDCl_3$) δ = 180.0, 158.1, 136.3, 132.9, 130.7, 130.5, 129.0, 128.3, 128.1, 127.7, 126.9, 125.4, 99.5, 48.1. HRMS (ESI): m/z $[M+H]^+$ calcd for $C_{16}H_{14}NOS$, 268.0791; found, 268.0790.

2-(Propylamino)-4*H*-thiochromen-4-one (4d): Off-white solid; yield: 170 mg (78%); mp 194-196 °C. ^1H NMR (400 MHz, $CDCl_3$) δ = 8.47-8.45 (m, 1H), 7.49-7.40 (m, 3H), 6.21 (s, 1H), 5.49 (brs, 1H), 3.30-3.25 (m, 2H), 1.77-1.71 (m, 2H), 1.02 (t, *J* = 7.2 Hz, 3H). ^{13}C NMR (100 MHz, $CDCl_3$) δ = 179.1, 159.0, 132.7, 130.7, 130.2, 128.3, 127.0, 125.3, 99.0, 46.0, 22.1, 11.4. HRMS (ESI): m/z $[M+H]^+$ calcd for $C_{12}H_{14}NOS$, 220.0791; found, 220.0791.

2-((4-Methoxyphenyl)amino)-4*H*-thiochromen-4-one (4e): Off-white solid; yield: 156 mg (55%); mp 189-191 °C. ^1H NMR (400 MHz, DMSO-d_6) δ = 9.65 (brs, 1H), 8.19 (d, *J* = 7.6 Hz, 1H), 7.64 (d, *J* = 7.6 Hz, 1H), 7.56 (t, *J* = 7.2 Hz, 1H), 7.46 (t, *J* = 7.6 Hz, 1H), 7.25 (d, *J* = 8.4 Hz, 2H), 7.00 (d, *J* = 8.4 Hz, 2H), 6.06 (s, 1H), 3.77 (s, 3H). ^{13}C NMR (100 MHz, DMSO-d_6) δ = 178.4, 158.4, 158.0, 133.2, 131.4, 131.1, 130.5, 127.6, 127.5, 127.0, 126.7, 115.3, 99.7, 55.8. HRMS (ESI): m/z $[M+H]^+$ calcd for $C_{16}H_{14}NO_2S$, 284.0740; found, 284.0740.

2-((3,4-Dimethoxybenzyl)amino)-4*H*-thiochromen-4-one (4f): Off-white solid; yield: 259 mg (79%); mp 152-154 °C. ^1H NMR (400 MHz, $CDCl_3$) δ = 8.45-8.43 (m, 1H), 7.47-7.39 (m, 3H), 6.90-6.83 (m, 3H), 6.15 (s, 1H), 5.20 (brs, 1H), 4.39 (d, *J* = 5.2 Hz, 2H), 3.88 (s, 3H), 3.87 (s, 3H).^{13}C NMR (100 MHz, $CDCl_3$) δ = 180.0, 157.6, 149.4, 149.0, 132.8, 130.8, 130.5, 128.6, 128.4, 127.0, 125.4, 120.3, 111.4, 111.0, 99.5, 56.0, 48.1. HRMS (ESI): m/z $[M+H]^+$ calcd for $C_{18}H_{18}NO_3S$, 328.1002; found, 328.1000.

2-((Cyclohexylmethyl)amino)-4*H*-thiochromen-4-one (4g): Off-white solid; yield: 208 mg (76%); mp 181-183 °C. ^{1}H NMR (400 MHz, CDCl$_3$) δ = 8.46 (d, *J* = 6.0 Hz, 1H), 7.48-7.40 (m, 3H), 6.14 (s, 1H), 5.20 (brs, 1H), 3.14 (t, *J* = 5.2 Hz, 2H), 1.82-1.68 (m, 5H), 1.28-1.17 (m, 4H), 1.03-0.98 (m, 2H). ^{13}C NMR (100 MHz, CDCl$_3$) δ = 179.5, 158.7, 132.7, 130.6, 130.4, 128.3, 126.9, 125.3, 99.0, 50.5, 37.2, 31.0, 26.3, 25.7. HRMS (ESI): m/z [M+H]$^+$ calcd for $C_{16}H_{20}NOS$, 274.1260; found, 274.1259.

2-(Cyclohexylamino)-4*H*-thiochromen-4-one (4h): Off-white solid; yield: 184 mg (71%); mp 238-240 °C. ^{1}H NMR (400 MHz, CDCl$_3$) δ = 8.46-8.44 (m, 1H), 7.50-7.38 (m, 3H), 6.17 (s, 1H), 5.15 (brs, 1H), 3.50-3.48 (m, 1H), 2.12-2.09 (m, 2H), 1.82-1.78 (m, 2H), 1.69-1.65 (m, 1H), 1.43-1.21 (m, 5H). ^{13}C NMR (100 MHz, CDCl$_3$) δ = 179.5, 157.5, 132.8, 130.6, 130.4, 128.3, 126.9, 125.2, 99.0, 53.1, 32.7, 25.4, 24.7. HRMS (ESI): m/z [M+H]$^+$ calcd for $C_{15}H_{18}NOS$, 260.1104; found, 260.1104.

2-(Diethylamino)-4*H*-thiochromen-4-one (4i): Off-white solid; yield: 156 mg (67%); mp 182-184 °C. ^{1}H NMR (400 MHz, CDCl$_3$) δ = 8.47-8.45 (m, 1H), 7.48-7.41 (m, 3H), 6.08 (s, 1H), 3.51 (q, *J* = 7.2 Hz, 4H), 1.28 (t, *J* = 7.2 Hz, 6H). ^{13}C NMR (100 MHz, CDCl$_3$) δ = 179.5, 157.6, 133.0, 130.3, 130.0, 128.0, 126.9, 125.4, 98.5, 45.6, 12.5. HRMS (ESI): m/z [M+H]$^+$ calcd for $C_{13}H_{16}NOS$, 234.0947; found, 234.0947.

2-(4-Benzylpiperazin-1-yl)-5-chloro-4*H*-thiochromen-4-one (4j): Yellow solid; yield: 250 mg (67%); mp 169-171 °C. ^{1}H NMR (400 MHz, CDCl$_3$) δ = 7.42-7.29 (m, 8H), 6.10 (s, 1H), 3.55 (s, 2H), 3.47 (brs, 4H), 2.56 (brs, 4H). ^{13}C NMR (100 MHz, CDCl$_3$) δ = 180.0, 156.6, 137.3, 136.3, 135.6, 131.1, 130.0, 129.1, 128.4, 127.4, 126.9, 124.9, 102.8, 62.7, 52.1, 47.4. HRMS (ESI): m/z [M+H]$^+$ calcd for $C_{20}H_{20}ClN_2OS$, 371.0979; found, 371.0983.

2-(4-Benzylpiperazin-1-yl)-7-fluoro-4*H*-thiochromen-4-one (4k): Yellow solid; yield: 250 mg (71%); mp 171-173 °C. ^{1}H NMR (400 MHz, CDCl$_3$) δ = 8.45-8.41 (m, 1H), 7.33-7.27 (m, 5H), 7.14-7.12 (m, 2H), 6.14 (s, 1H), 3.56 (s, 2H), 3.51-3.50 (m, 4H), 2.57 (brs, 4H). ^{13}C NMR (100 MHz, CDCl$_3$) δ = 179.4, 163.6 (d, *J* = 252 Hz), 159.0, 137.3, 135.1, 130.9, 129.1, 128.4, 127.5, 126.5, 115.2 (d, *J* = 22 Hz), 111.7 (d, *J* = 25 Hz), 100.9, 62.7, 52.1, 47.7. HRMS (ESI): m/z [M+H]$^+$ calcd for $C_{20}H_{20}FN_2OS$, 355.1275; found, 355.1276.

2-(4-Benzylpiperazin-1-yl)-7,8-dichloro-4*H*-thiochromen-4-one (4l): Yellow solid; yield: 316 mg (78%); mp 214-216 °C. ^{1}H NMR (400 MHz, $CDCl_3$) δ = 8.32 (d, *J* = 8.8 Hz, 1H), 7.53 (d, *J* = 8.4 Hz, 1H), 7.35-7.29 (m, 5H), 6.16 (s, 1H), 3.59-3.57 (m, 6H), 2.60 (brs, 4H). ^{13}C NMR (100 MHz, $CDCl_3$) δ = 179.4, 159.1, 137.2, 135.9, 134.3, 130.3, 129.1, 128.5, 128.1, 127.9, 127.5, 127.4, 100.4, 62.7, 52.1, 47.8. HRMS (ESI): m/z $[M+H]^+$ calcd for $C_{20}H_{19}Cl_2N_2OS$, 405.0590; found, 405.0590.

2-(4-Benzylpiperazin-1-yl)-6-(trifluoromethyl)-4*H*-thiochromen-4-one (4m): Yellow solid; yield: 330 mg (82%); mp 124-126 °C. ^{1}H NMR (400 MHz, $CDCl_3$) δ = 8.72-8.71 (m, 1H), 7.71-7.68 (m, 1H), 7.54 (d, *J* = 8.4 Hz, 1H), 7.35-7.29 (m, 5H), 6.21 (s, 1H), 3.57-3.54 (m, 6H), 2.60-2.58 (m, 4H). ^{13}C NMR (100 MHz, $CDCl_3$) δ = 179.1, 159.1, 137.2, 136.8, 130.3, 129.5 (q, $^{2}J_{F, C}$ = 33 Hz), 129.1, 128.5, 127.5, 126.8, 126.4, 125.5, 123.7 (q, $^{1}J_{F, C}$ = 271 Hz), 101.0, 62.7, 52.1, 47.8. HRMS (ESI): m/z $[M+H]^+$ calcd for $C_{21}H_{20}F_3N_2OS$, 405.1243; found, 405.1243.

2-(4-Benzylpiperazin-1-yl)-8-iodo-6-(trifluoromethyl)-4*H*-thiochromen-4-one (4n): Yellow solid; yield: 435 mg (82%); mp 165-167 °C. ^{1}H NMR (400 MHz, $CDCl_3$) δ = 8.75 (s, 1H), 8.17 (s, 1H), 7.35-7.29 (m, 5H), 6.17 (s, 1H), 3.62-3.58 (m, 6H), 2.62-2.60 (m, 4H). ^{13}C NMR (100 MHz, $CDCl_3$) δ = 179.4, 159.4, 141.0, 137.4, 137.1, 132.5, 130.3 (q, $^{2}J_{F, C}$ = 34 Hz), 129.1, 128.4, 127.5, 125.5, 122.7 (q, $^{1}J_{F, C}$ = 271 Hz), 99.7, 94.6, 62.7, 52.1, 47.8. HRMS (ESI): m/z $[M+H]^+$ calcd for $C_{21}H_{19}F_3IN_2OS$, 531.0209; found, 531.0210.

2-(4-Benzylpiperazin-1-yl)-8-methyl-4*H*-thiochromen-4-one (4o): Yellow solid; yield: 285 mg (81%); mp 162-164 °C. ^{1}H NMR (400 MHz, $CDCl_3$) δ = 8.33 (d, *J* = 4.8 Hz, 1H), 7.36-7.35 (m, 6H), 7.30 (d, *J* = 2.4 Hz, 1H), 6.22 (s, 1H), 3.58 (brs, 6H), 2.61 (brs, 4H), 2.46 (s, 3H). ^{13}C NMR (100 MHz, $CDCl_3$) δ = 181.0, 158.8, 137.2, 133.7, 132.5, 132.2, 130.2, 129.2, 128.5, 127.5, 126.3, 126.0, 101.3, 62.7, 52.2, 47.6, 19.5. HRMS (ESI): m/z $[M+H]^+$ calcd for $C_{21}H_{23}N_2OS$, 351.1526; found, 351.1522.

2-(Benzylamino)-8-methyl-4*H*-thiochromen-4-one (4p): Off-white solid; yield: 200 mg (71%); mp 208-210 °C. ^{1}H NMR (400 MHz, DMSO-d_6) δ = 8.46 (t, *J* = 6.0 Hz, 1H), 8.07 (d,

J = 7.6 Hz, 1H), 7.47 (d, *J* = 7.2 Hz, 1H), 7.38-7.33 (m, 4H), 7.37 (brs, 1H), 7.30-7.27 (m, 1H), 5.83 (s, 1H), 4.48 (d, *J* = 6.0 Hz, 2H), 2.40 (s, 3H). ^{13}C NMR (100 MHz, DMSO-d_6) δ = 178.7, 157.1, 138.1, 133.9, 132.6, 132.4, 130.8, 129.0, 127.7, 127.6, 126.4, 125.6, 97.5, 46.8, 19.3. HRMS (ESI): m/z $[M+H]^+$ calcd for $C_{17}H_{16}NOS$, 282.0947; found, 282.0947.

8-Methyl-2-(propylamino)-4*H*-thiochromen-4-one (4q): Off-white solid; yield: 180 mg (77%); mp 178-180 °C. ^{1}H NMR (400 MHz, $CDCl_3$) δ = 8.35 (t, *J* = 4.0 Hz, 1H), 7.34-7.31 (m, 2H), 6.14 (s, 1H), 5.44 (brs, 1H), 3.29-3.25 (m, 2H), 2.41 (s, 3H), 1.75-1.71 (m, 2H), 1.02 (t, *J* =6.0 Hz, 3H). ^{13}C NMR (100 MHz, $CDCl_3$) δ = 180.1, 157.9, 133.4, 132.3, 132.0, 130.7, 126.2, 126.1, 98.6, 45.9, 22.1, 19.4, 11.4. HRMS (ESI): m/z $[M+H]^+$ calcd for $C_{13}H_{16}NOS$, 234.0947; found, 234.0946.

7-Chloro-2-(phenylamino)-4*H*-thiochromen-4-one (4r): Off-white solid; yield: 153 mg (53%); mp 264-266 °C. ^{1}H NMR (400 MHz, DMSO-d_6) δ = 9.86 (brs, 1H), 8.17 (d, *J* = 7.2 Hz, 1H), 7.94 (s, 1H), 7.51 (d, *J* = 6.8 Hz, 1H), 7.44 (t, *J* = 6.0 Hz, 2H), 7.31 (d, *J* = 6.0 Hz, 2H), 7.24 (t, *J* = 5.6 Hz, 1H), 6.17 (s, 1H). ^{13}C NMR (100 MHz, DMSO-d_6) δ = 177.9, 156.8, 138.6, 136.4, 135.2, 130.1, 129.5, 129.2, 127.9, 126.2, 126.1, 124.2, 100.6. HRMS (ESI): m/z $[M+H]^+$ calcd for $C_{15}H_{11}ClNOS$, 288.0244; found, 288.0247.

7-Chloro-2-((3,4-dimethoxyphenyl)amino)-4*H*-thiochromen-4-one (4s): Yellow solid; yield: 177 mg (51%); mp 250-252 °C. ^{1}H NMR (400 MHz, DMSO-d_6) δ = 9.74 (brs, 1H), 8.17 (d, *J* = 6.8 Hz, 1H), 7.91 (s, 1H), 7.52-7.50 (m, 1H), 7.02 (d, *J* = 6.8 Hz, 1H), 6.91 (s, 1H), 6.88 (d, *J* = 6.8 Hz, 1H), 6.07 (s, 1H), 3.79 (s, 3H), 3.78 (s, 3H). ^{13}C NMR (100 MHz, DMSO-d_6) δ = 177.6, 158.2, 149.8, 147.8, 136.2, 135.2, 131.0, 129.4, 129.3, 127.7, 126.0, 117.7, 112.6, 109.9, 99.7, 56.2. HRMS (ESI): m/z $[M+H]^+$ calcd for $C_{17}H_{15}ClNO_3S$, 348.0456; found, 348.0457.

2-(4-Benzylpiperazin-1-yl)-7-chloro-4*H*-thiochromen-4-one (4t): Yellow solid; yield: 297 mg (80%); mp 129-131 °C. ^{1}H NMR (400 MHz, $CDCl_3$) δ = 8.35 (d, *J* = 8.4 Hz, 1H), 7.43-7.31 (m, 7H), 6.16 (s, 1H), 3.57 (s, 2H), 3.54-3.51 (m, 4H), 2.60-2.57 (m, 4H). ^{13}C NMR (100 MHz, $CDCl_3$) δ = 179.5, 159.0, 137.3, 137.1, 134.6, 129.7, 129.1, 128.5, 128.4, 127.7, 127.5,

124.9, 101.1, 62.7, 52.1, 47.7. HRMS (ESI): m/z $[M+H]^+$ calcd for $C_{20}H_{20}ClN_2OS$, 371.0979; found, 371.0983.

7-Chloro-2-morpholino-4*H*-thiochromen-4-one (4u): Yellow solid; yield: 245 mg (87%); mp 199-201 °C. ^{1}H NMR (400 MHz, $CDCl_3$) δ = 8.36 (d, *J* = 8.4 Hz, 1H), 7.45 (d, *J* = 2.0 Hz, 1H), 7.42-7.40 (m, 1H), 6.20 (s, 1H), 3.85-3.83 (m, 4H), 3.50-3.48 (m, 4H). ^{13}C NMR (100 MHz, $CDCl_3$) δ = 179.5, 159.4, 137.3, 134.4, 129.7, 128.4, 127.8, 125.0, 101.4, 66.0, 47.7. HRMS (ESI): m/z $[M+H]^+$ calcd for $C_{13}H_{13}ClNO_2S$, 282.0350; found, 282.0352.

Copies of ^{1}H and ^{13}C NMR spectra.

^{1}H and ^{13}C NMR spectra of 2-(ethylthio)-4*H*-thiochromen-4-one **(1a)**

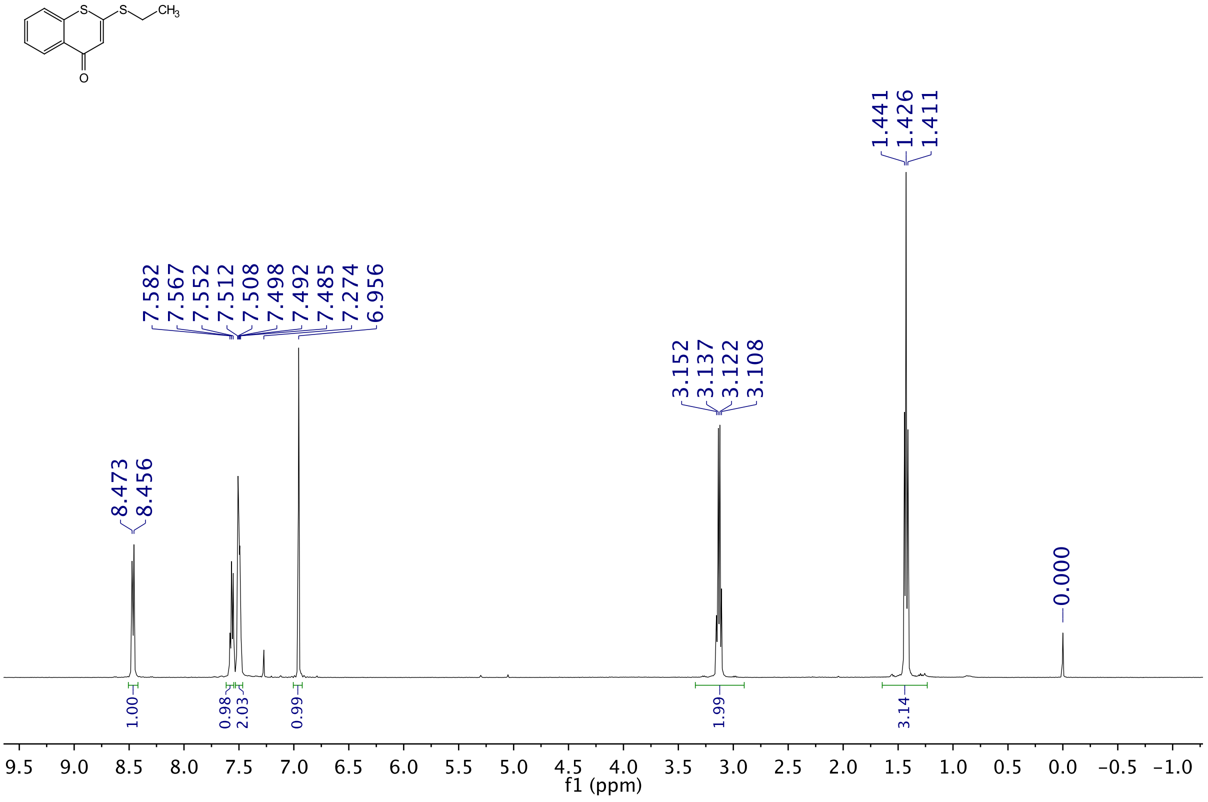

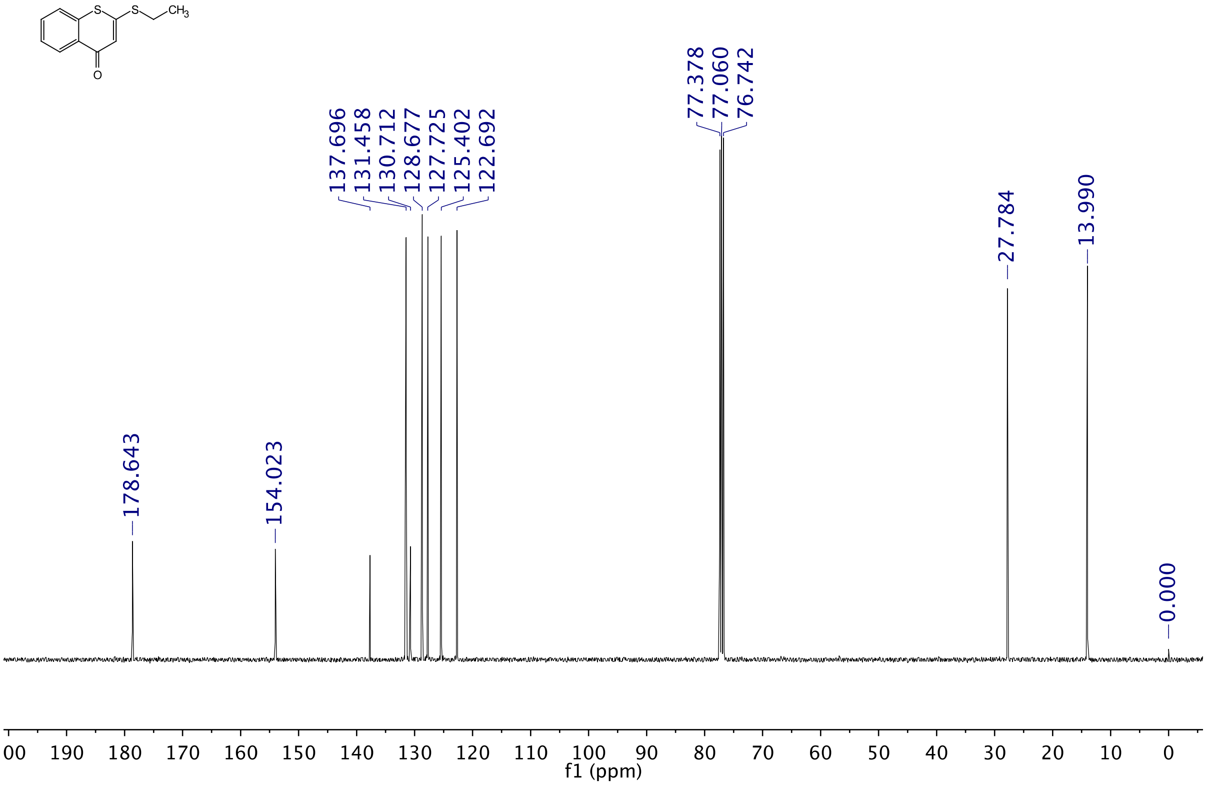

^{1}H and ^{13}C NMR spectra of 5-chloro-2-(ethylthio)-4*H*-thiochromen-4-one **(1b)**

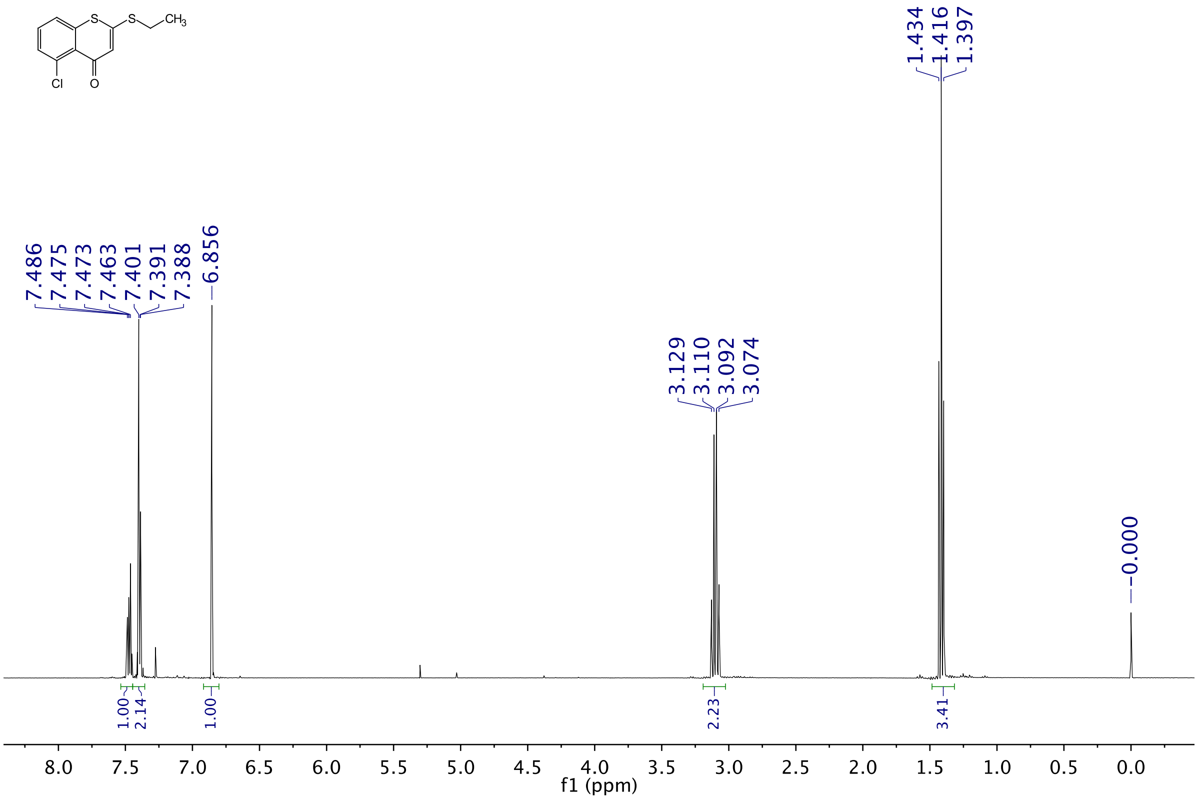

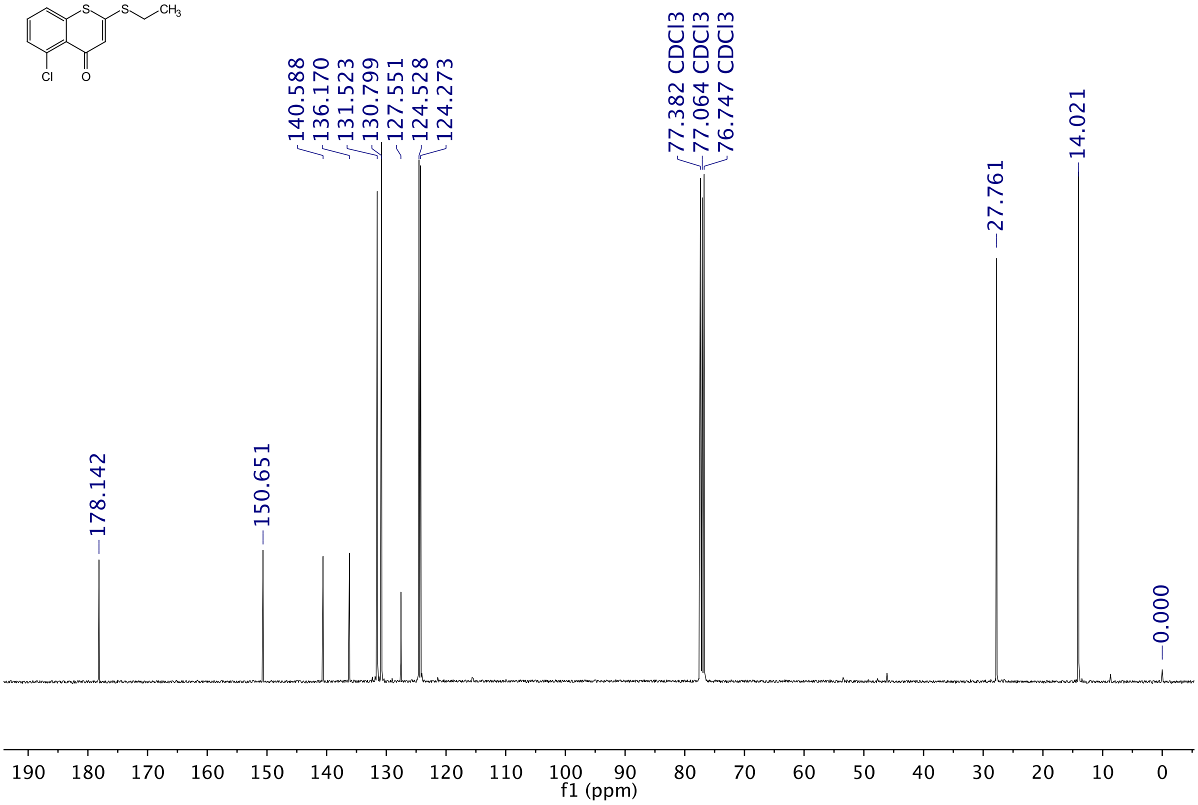

^{1}H and ^{13}C NMR spectra of 2-(ethylthio)-7-fluoro-4*H*-thiochromen-4-one **(1c)**

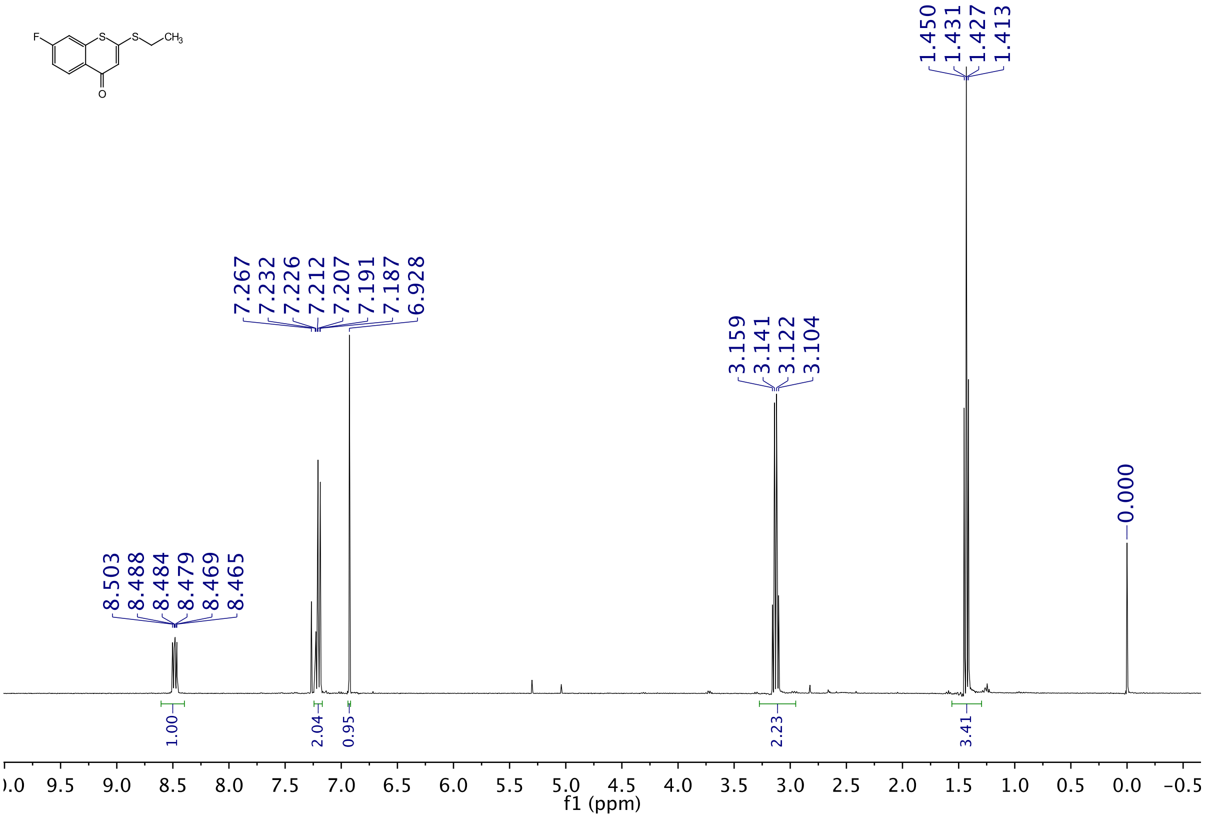

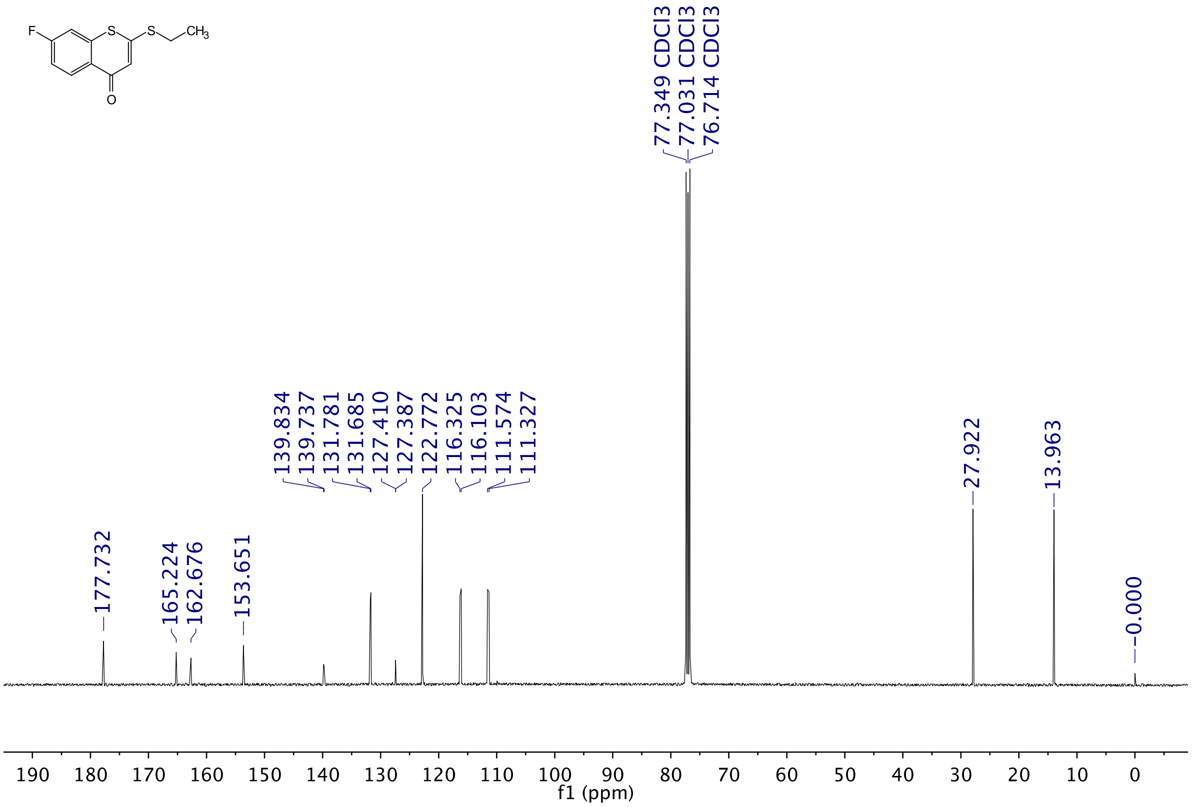

^{1}H and ^{13}C NMR spectra of 7,8-dichloro-2-(ethylthio)-4*H*-thiochromen-4-one **(1d)**

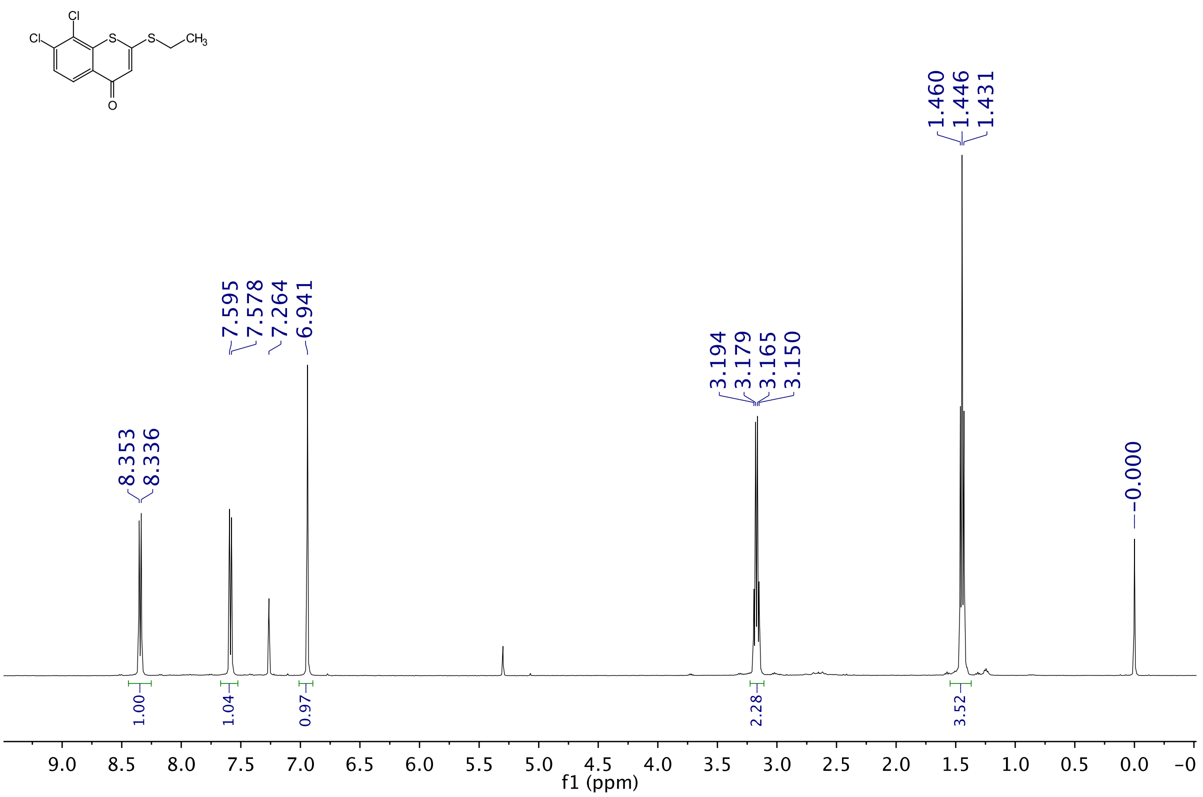

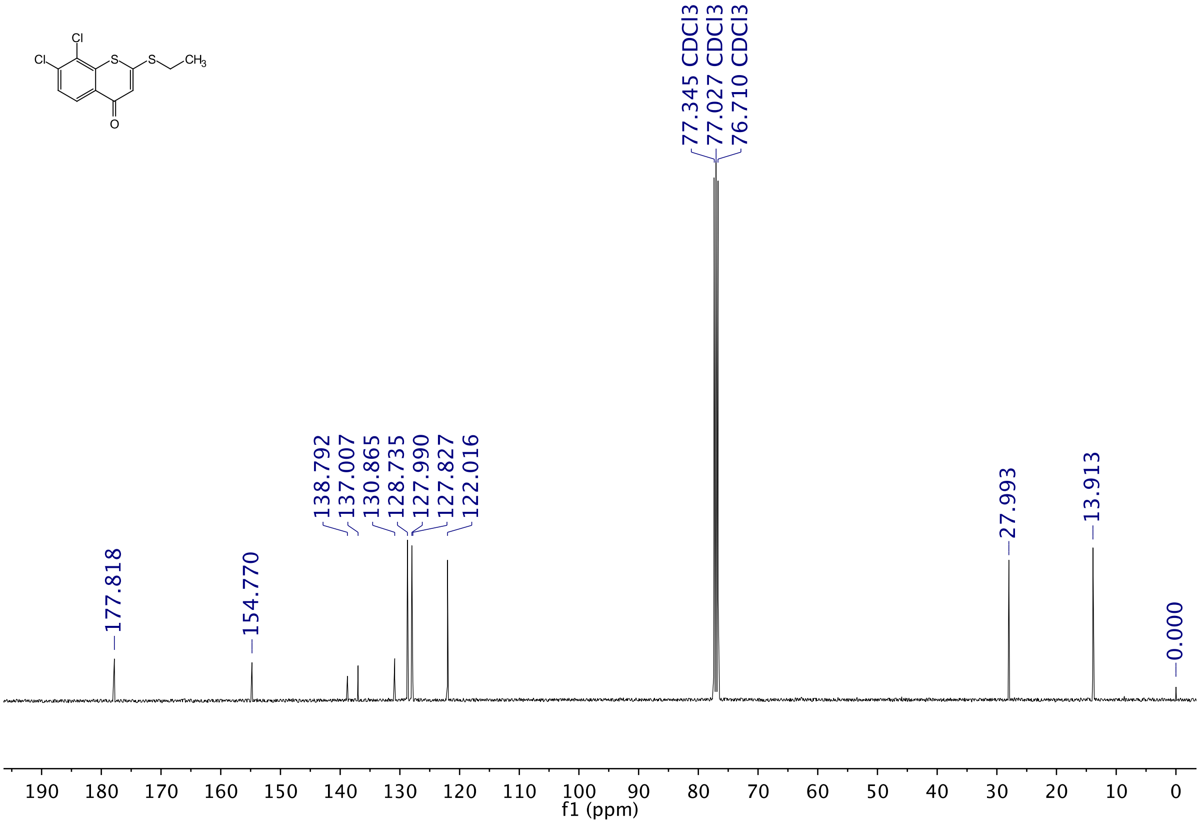

^{1}H and ^{13}C NMR spectra of 2-(ethylthio)-6-(trifluoromethyl)-4*H*-thiochromen-4-one **(1e)**

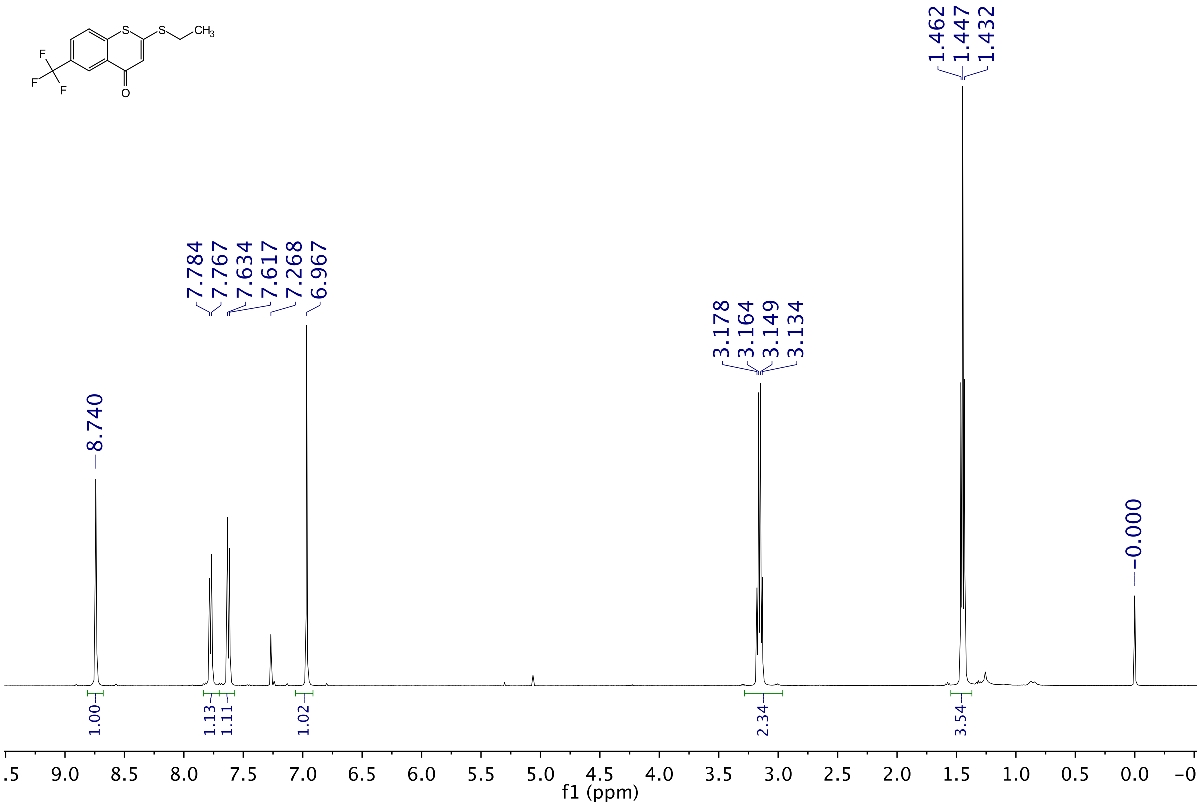

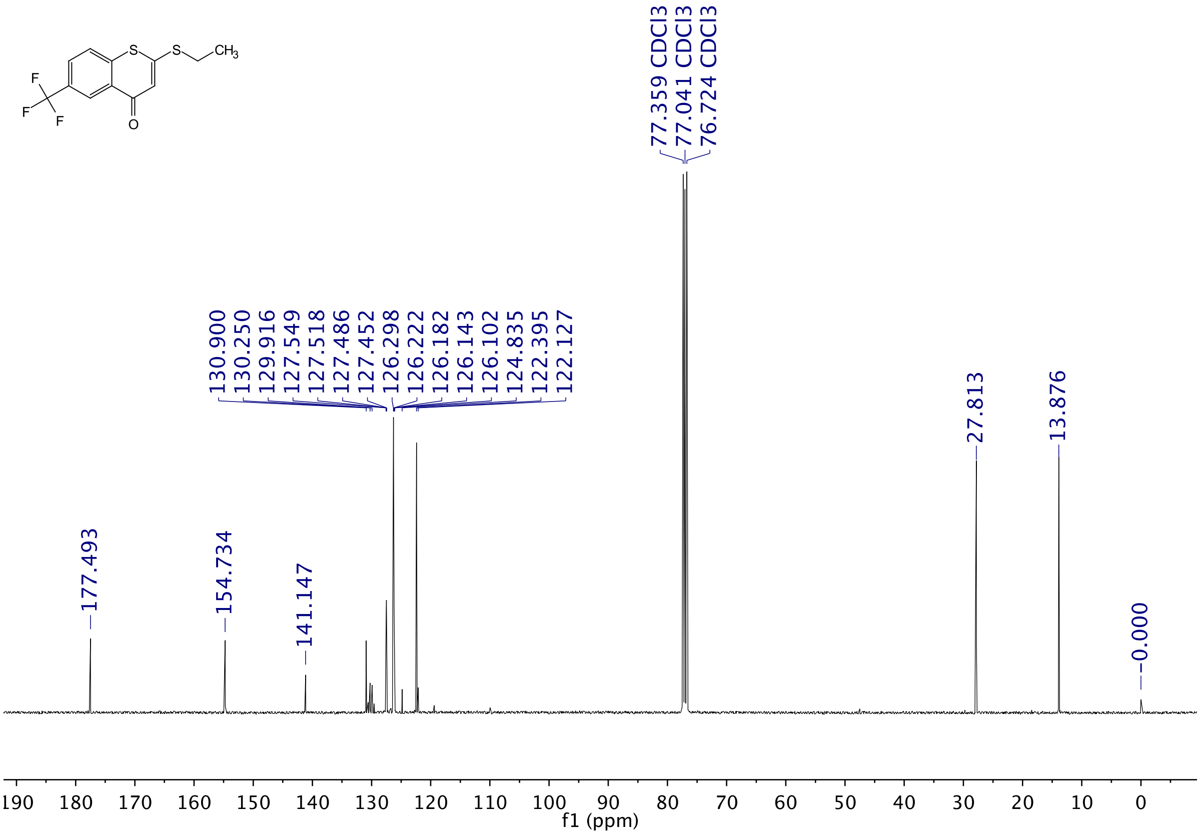

^1H and ^{13}C NMR spectra of 2-(ethylthio)-8-iodo-6-(trifluoromethyl)-4*H*-thiochromen-4-one **(1f)**

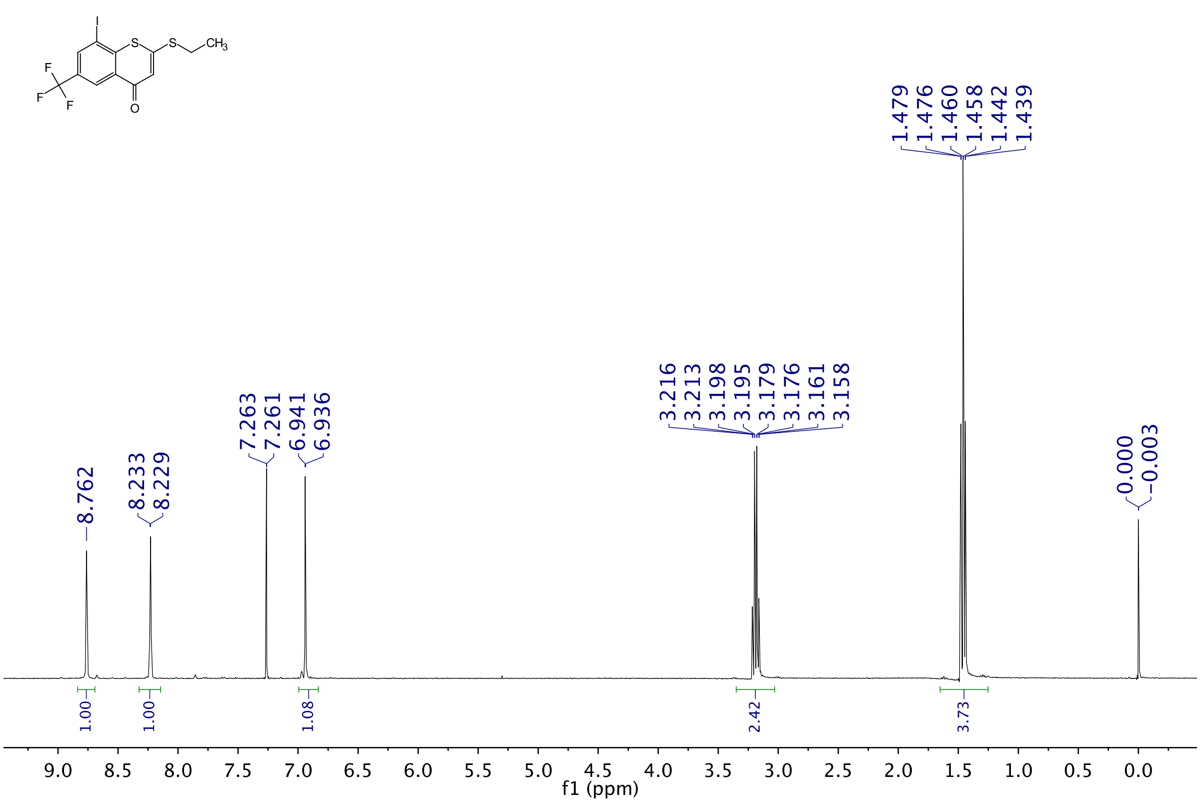

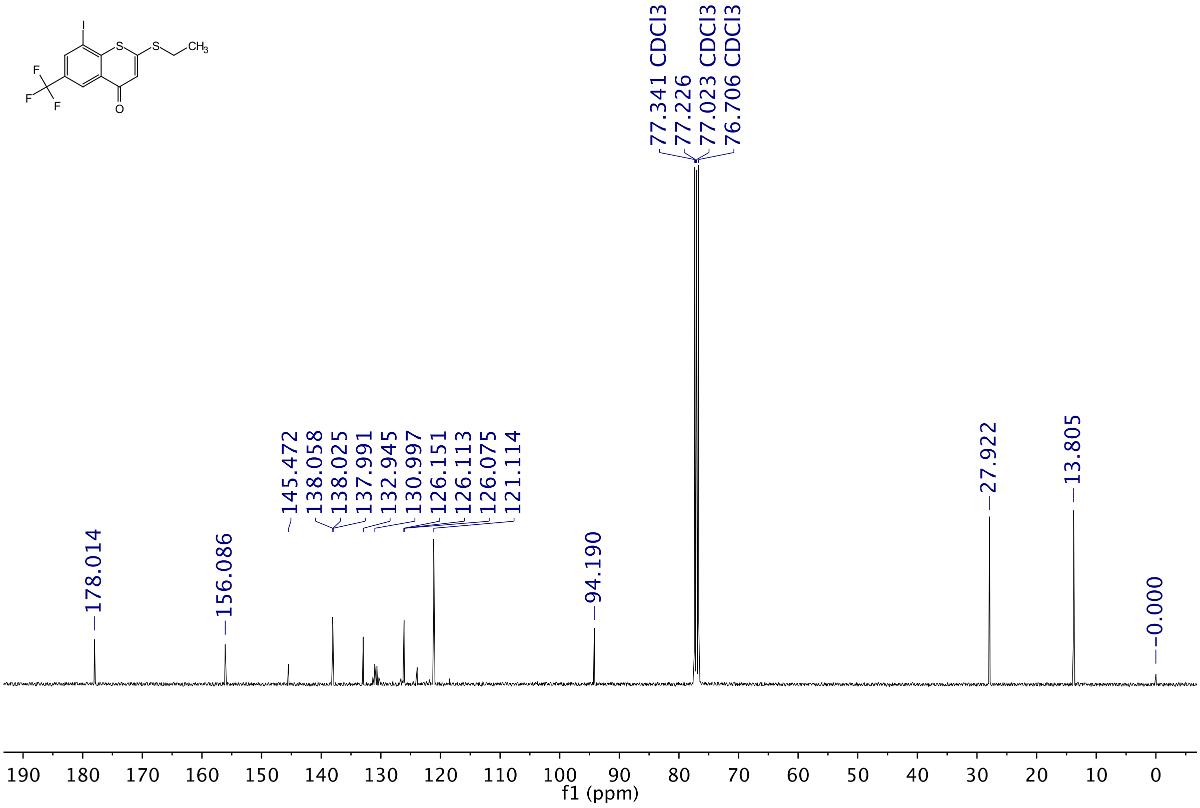

^{1}H and ^{13}C NMR spectra of 2-(ethylthio)-8-methyl-4*H*-thiochromen-4-one **(1g)**

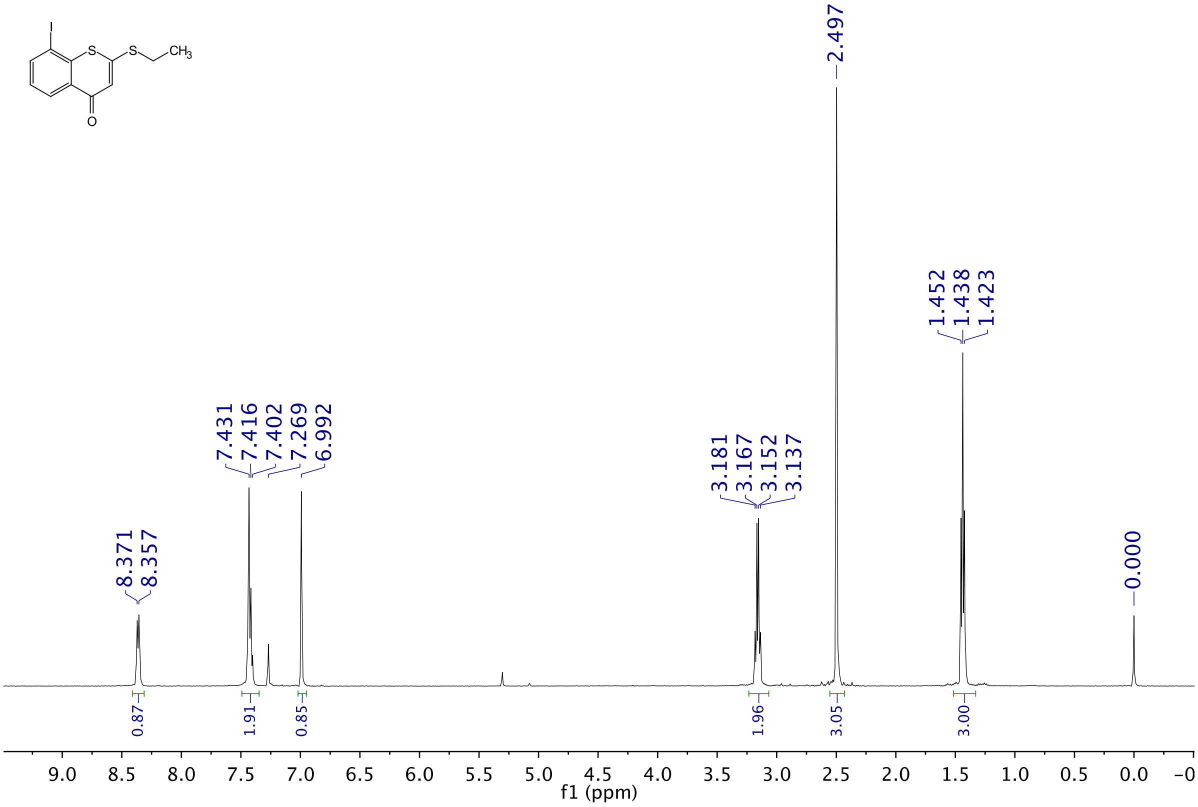

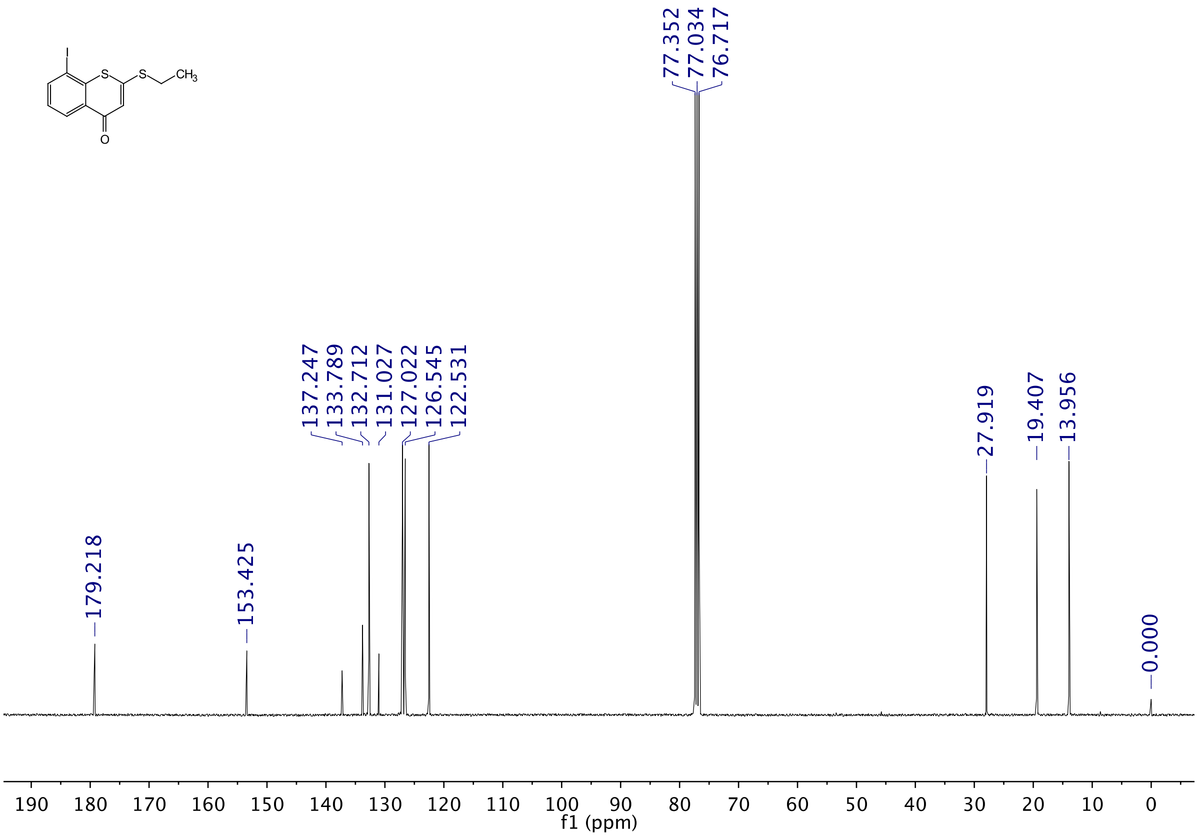

^{1}H and ^{13}C NMR spectra of 7-chloro-2-(ethylthio)-4*H*-thiochromen-4-one **(1h)**

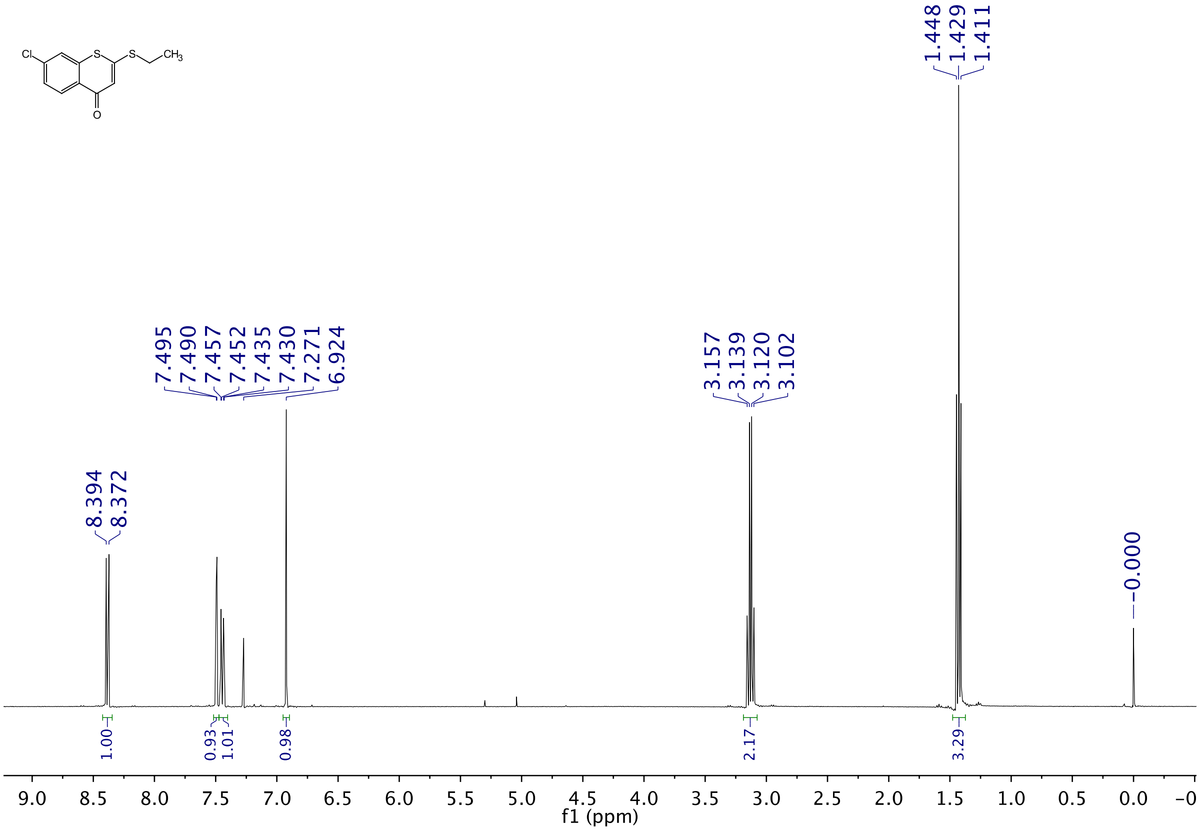

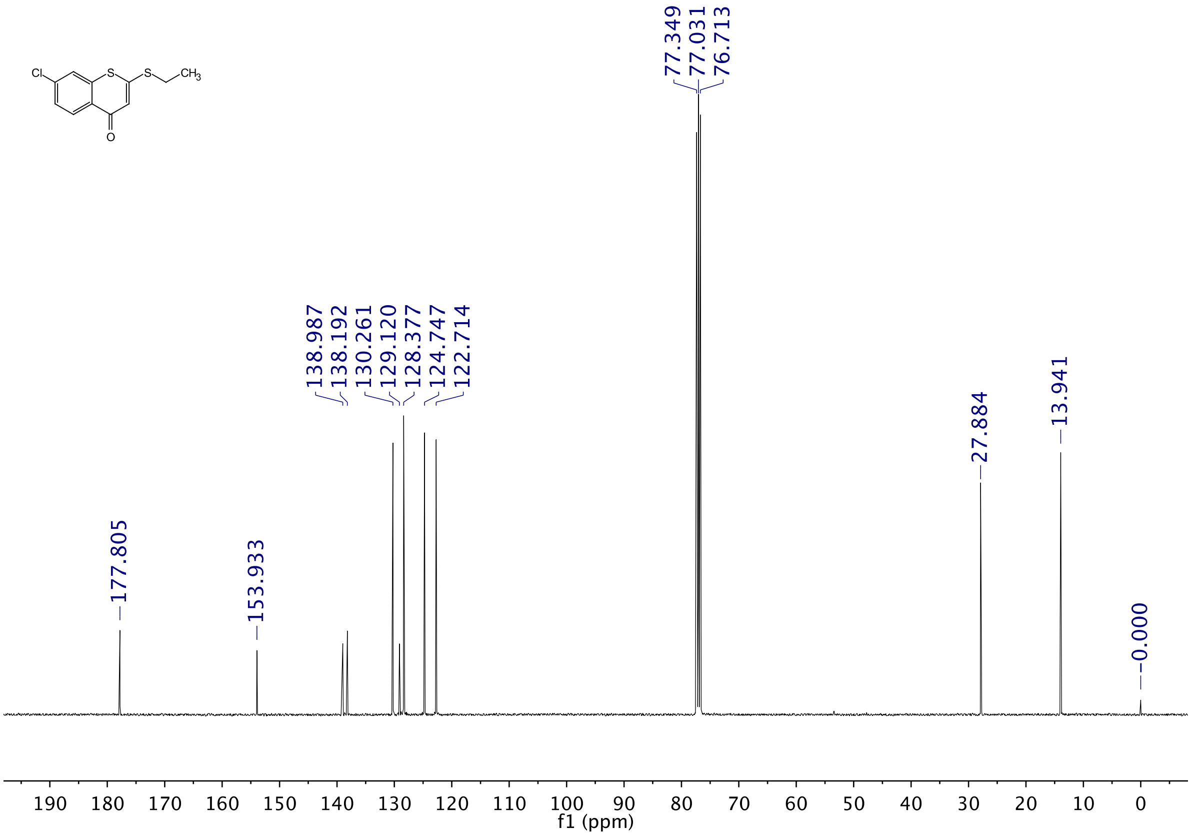

^{1}H and ^{13}C NMR spectra of 2-(ethylsulfinyl)-4*H*-thiochromen-4-one **(2a)**

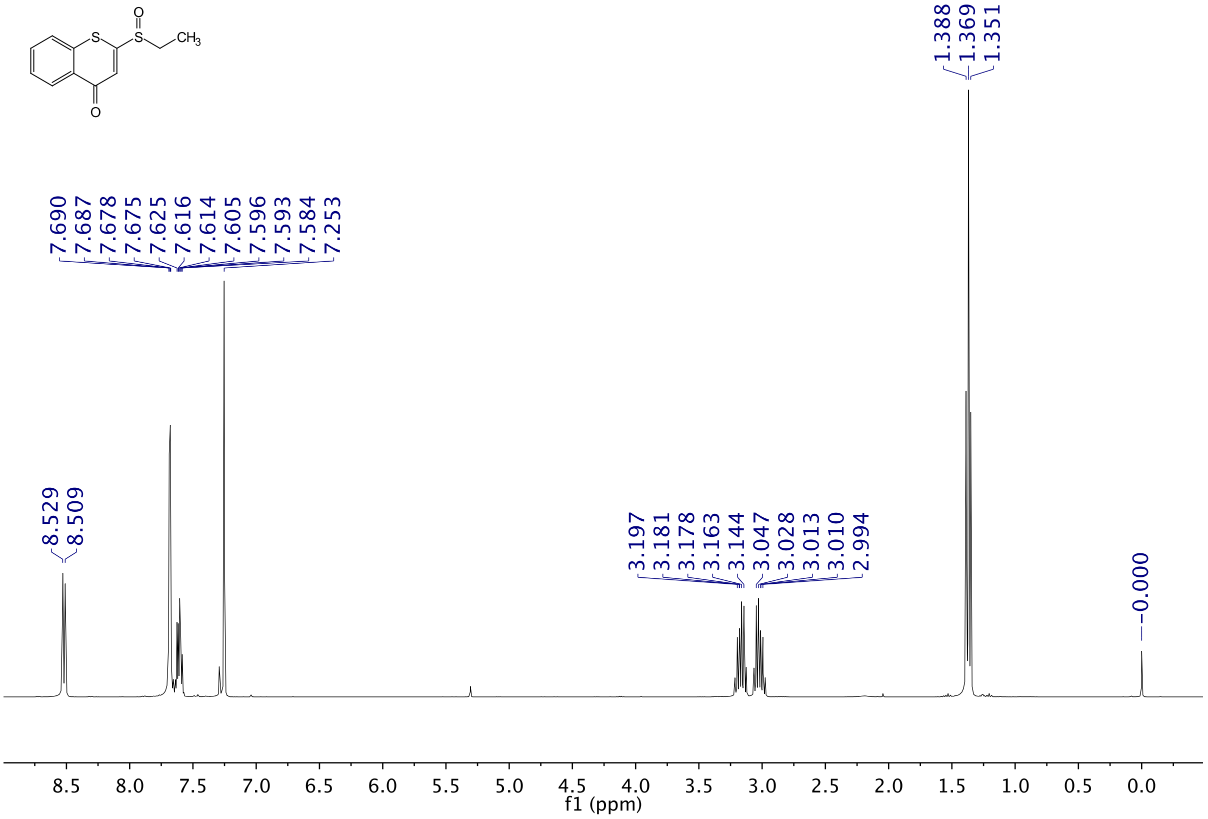

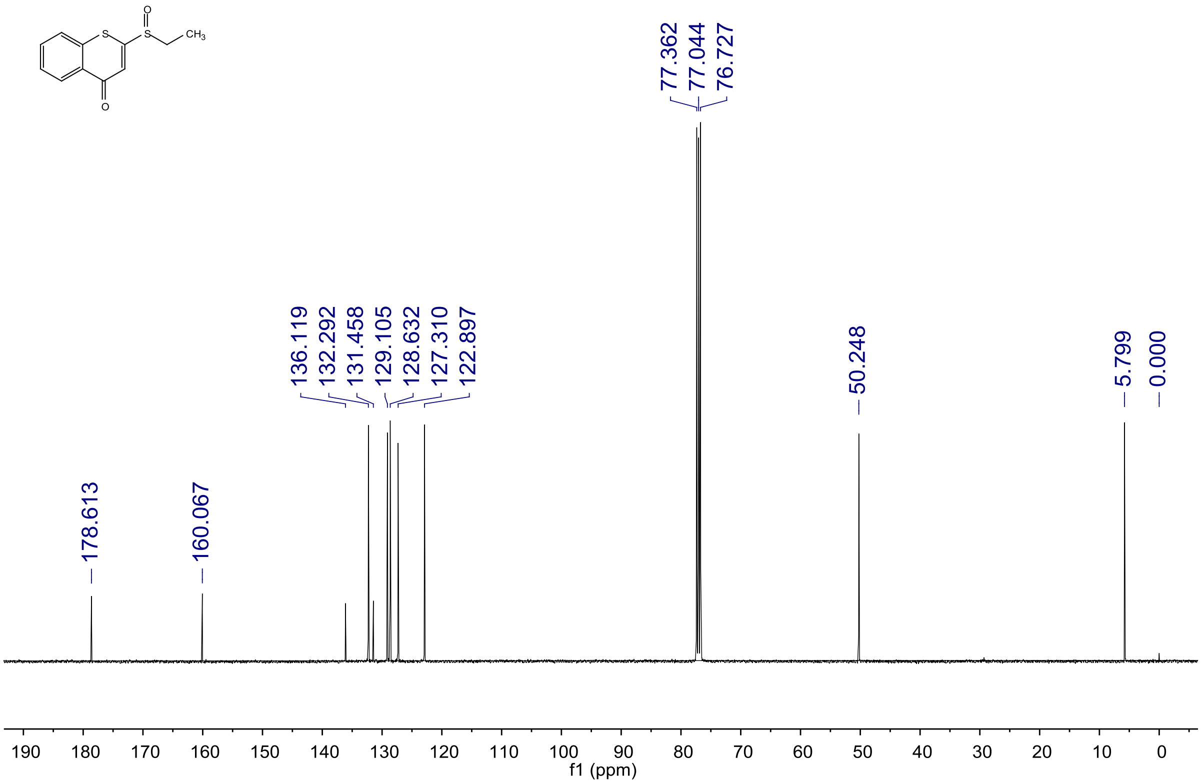

^{1}H and ^{13}C NMR spectra of 5-chloro-2-(ethylsulfinyl)-4*H*-thiochromen-4-one **(2b)**

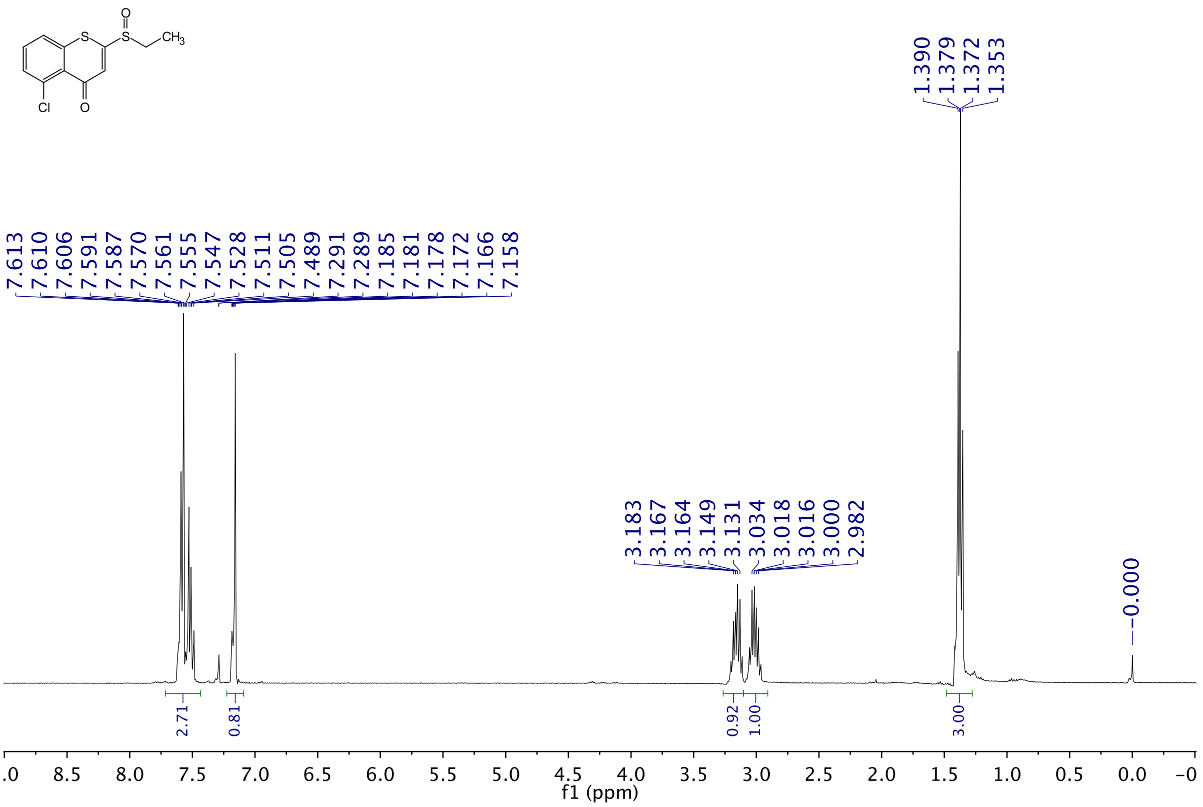

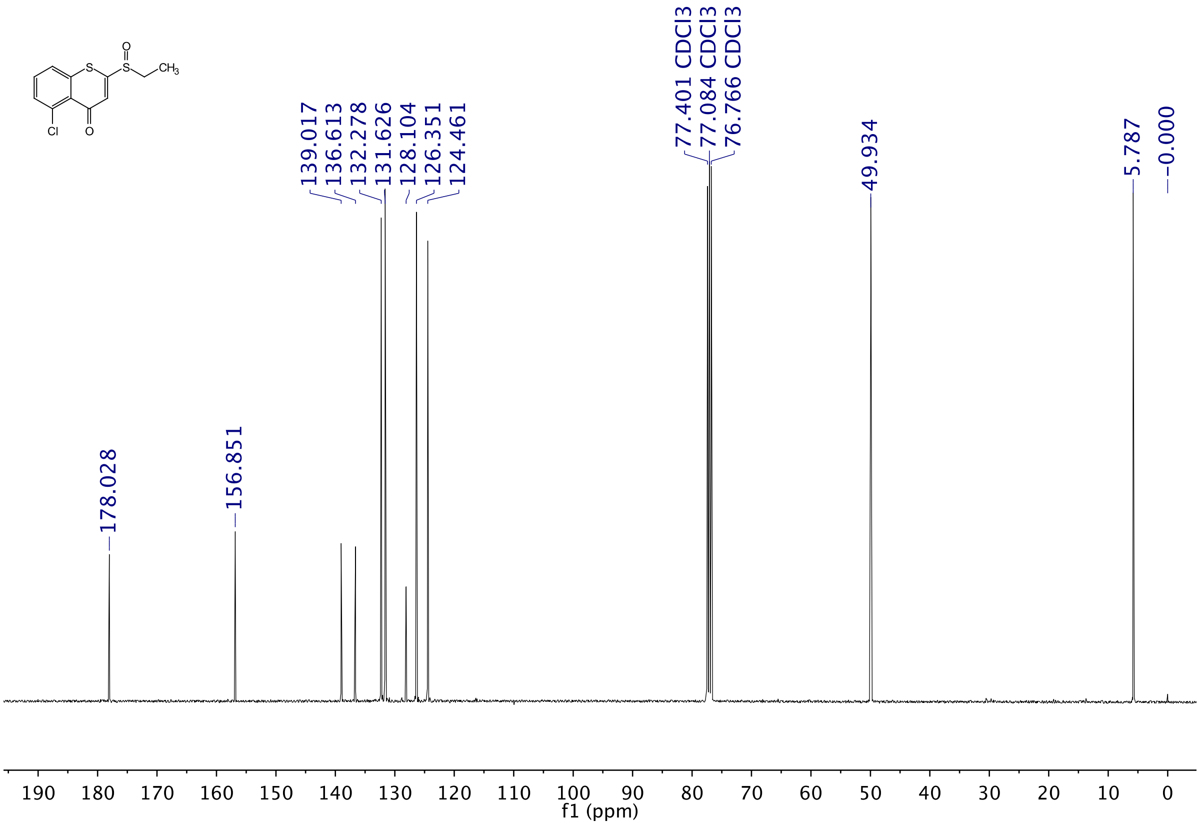

^{1}H and ^{13}C NMR spectra of 2-(ethylsulfinyl)-7-fluoro-4*H*-thiochromen-4-one **(2c)**

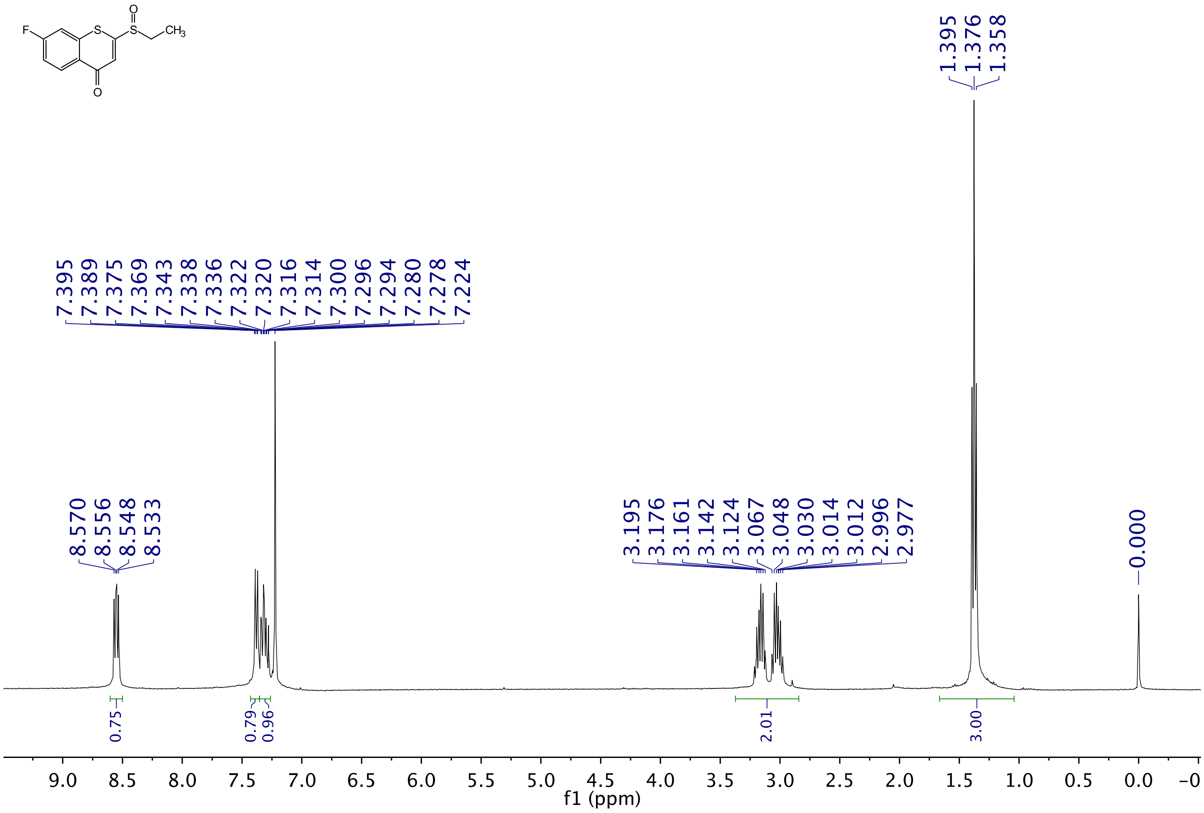

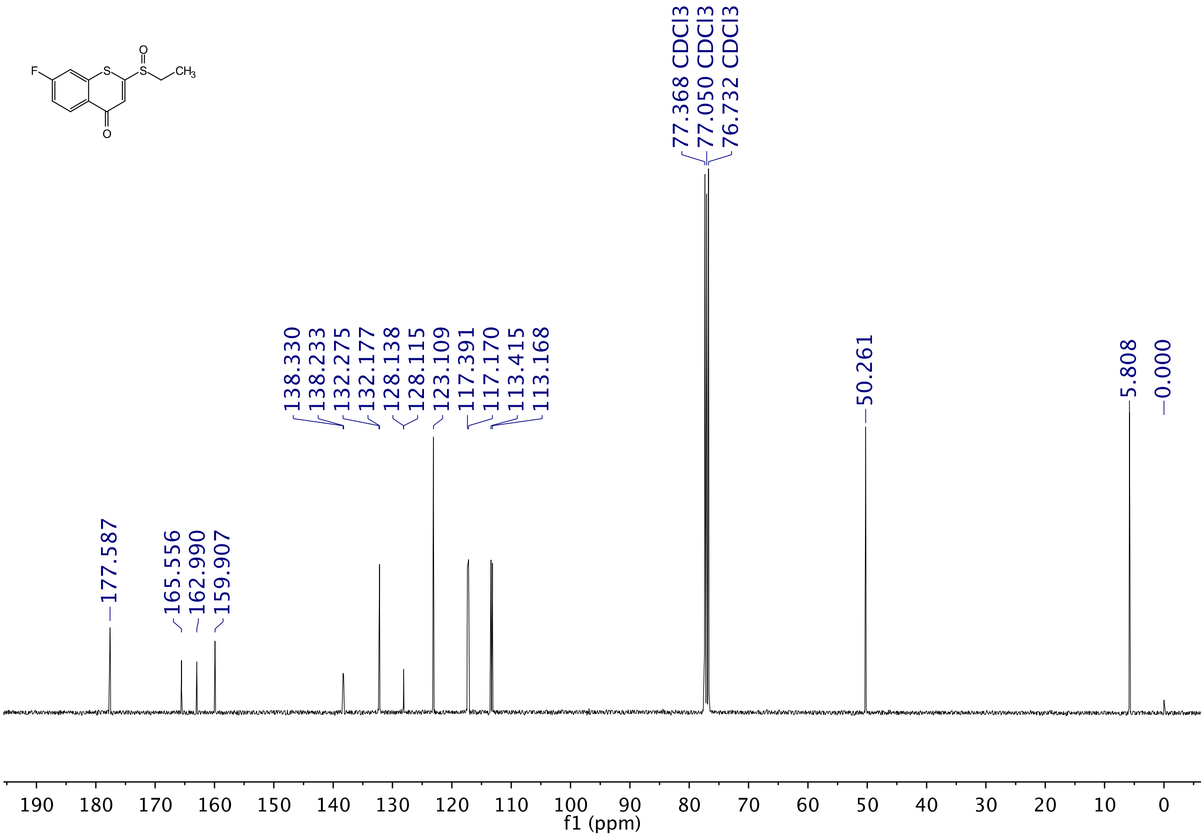

^{1}H and ^{13}C NMR spectra of 7,8-dichloro-2-(ethylsulfinyl)-4*H*-thiochromen-4-one **(2d)**

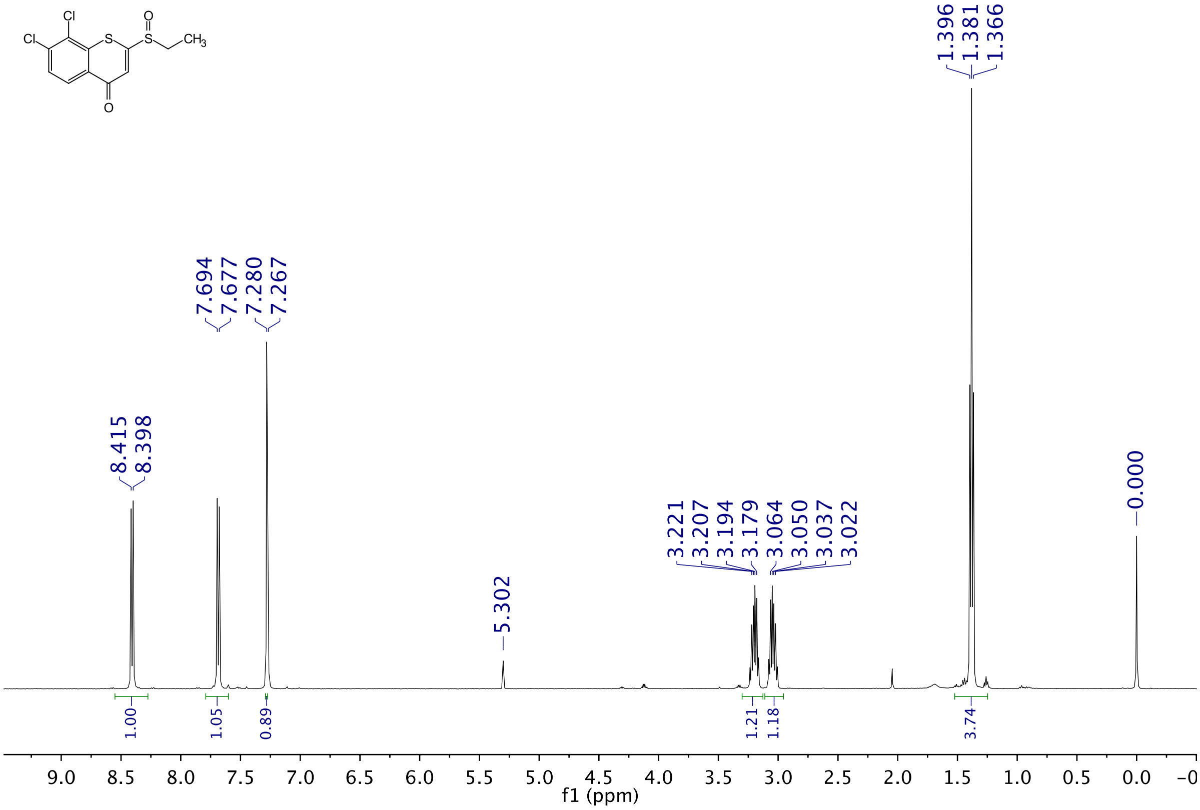

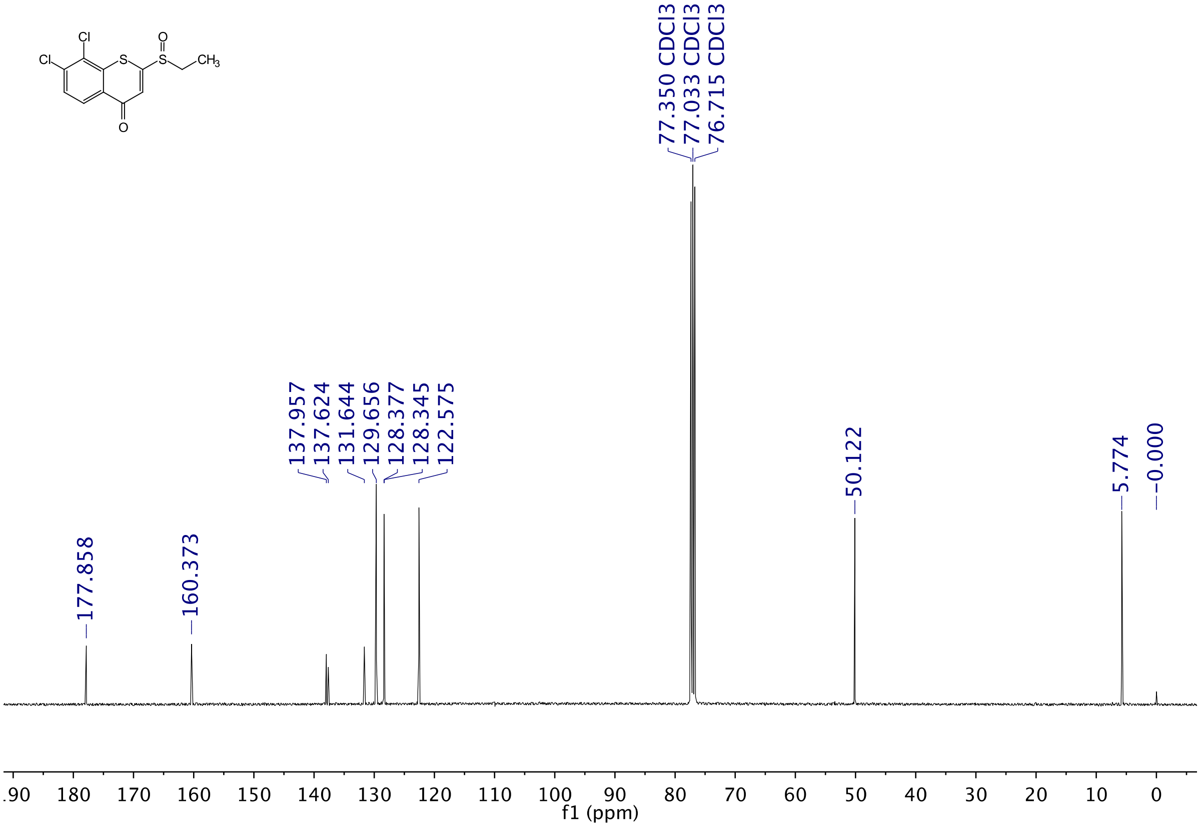

^{1}H and ^{13}C NMR spectra of 2-(ethylsulfinyl)-6-(trifluoromethyl)-4*H*-thiochromen-4-one **(2e)**

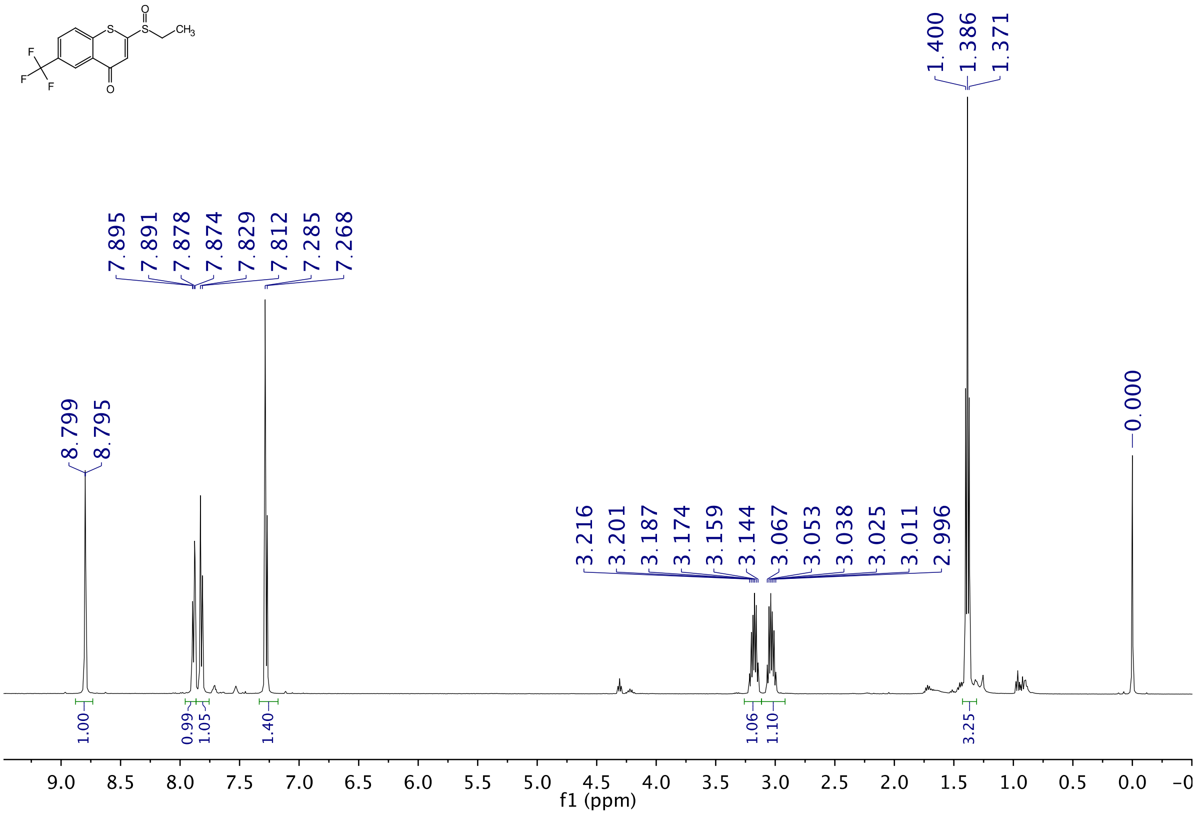

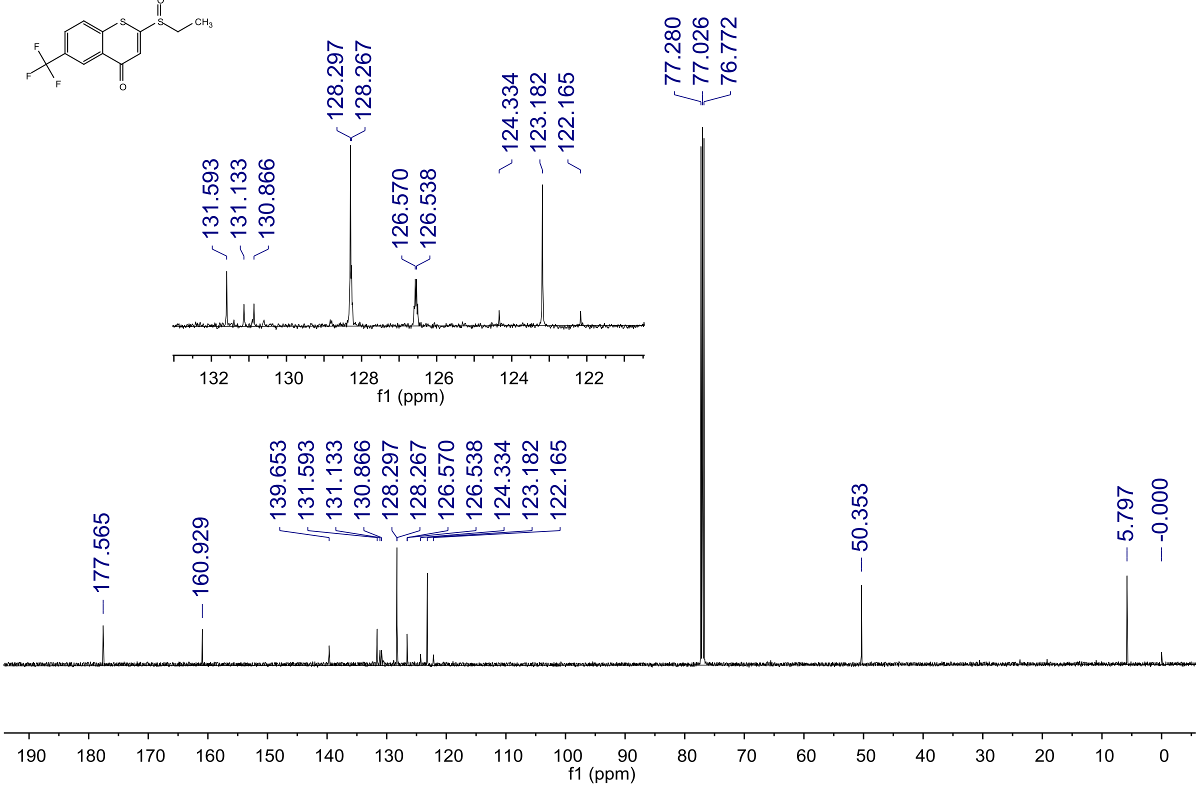

^{1}H and ^{13}C NMR spectra of 2-(ethylsulfinyl)-8-iodo-6-(trifluoromethyl)-4*H*-thiochromen-4-one **(2f)**

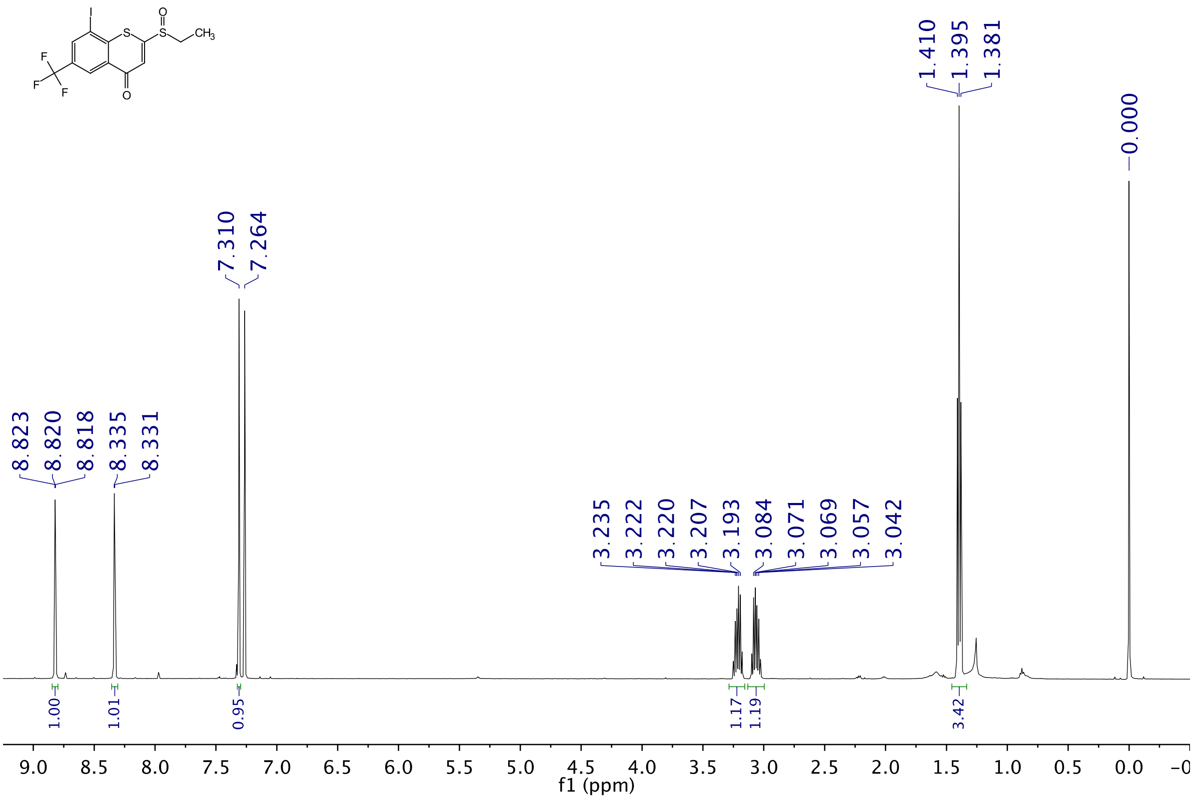

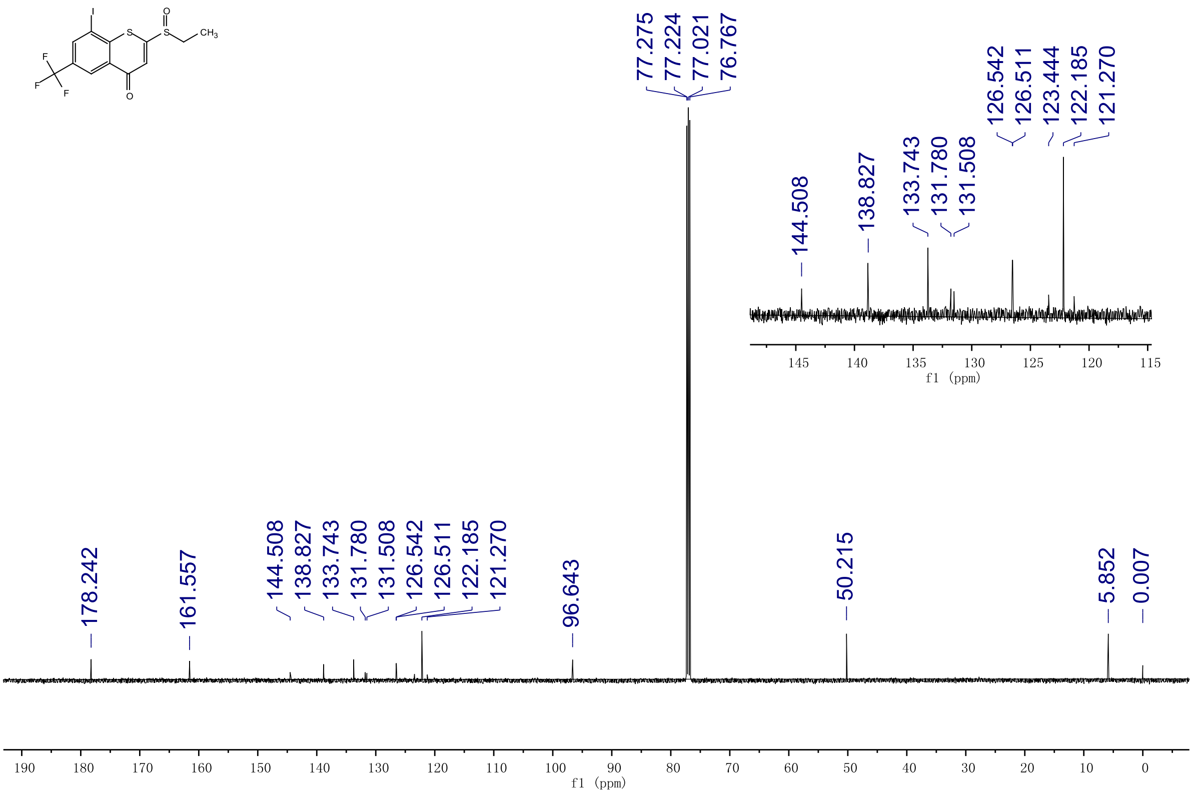

^{1}H and ^{13}C NMR spectra of 2-(ethylsulfinyl)-8-methyl-4*H*-thiochromen-4-one **(2g)**

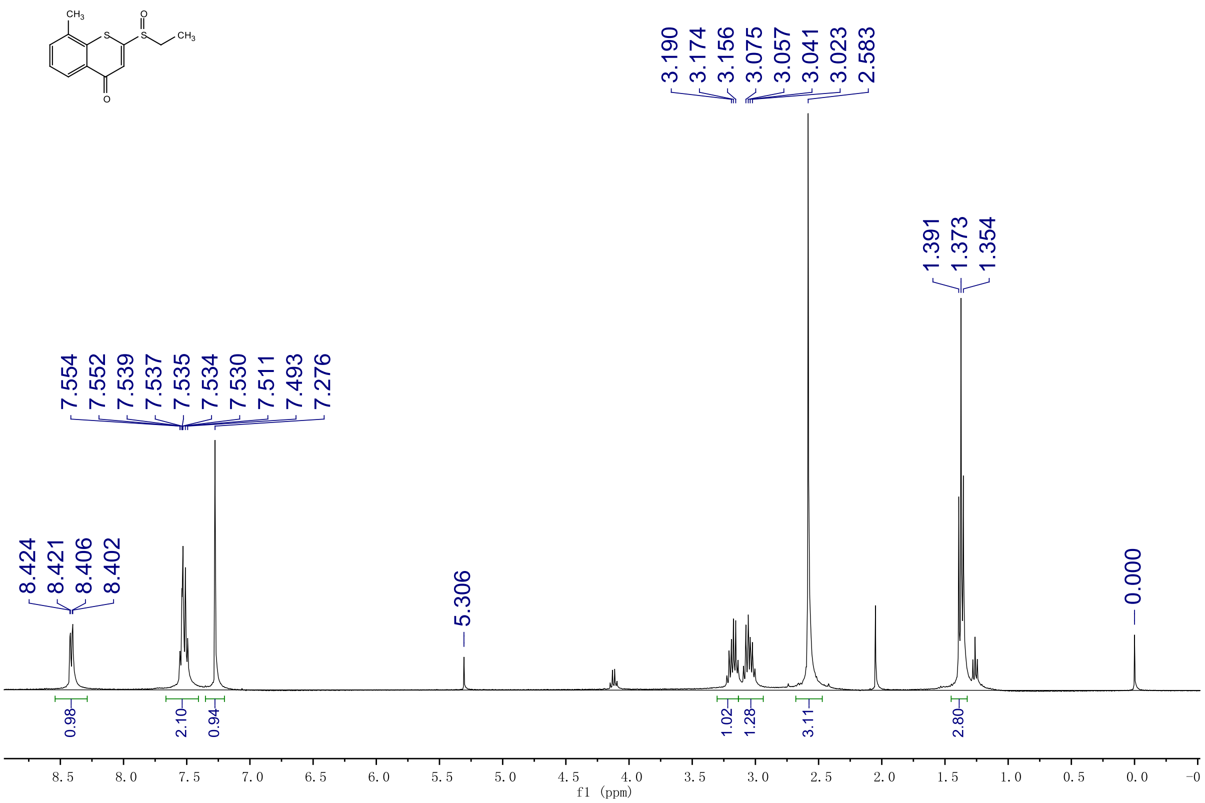

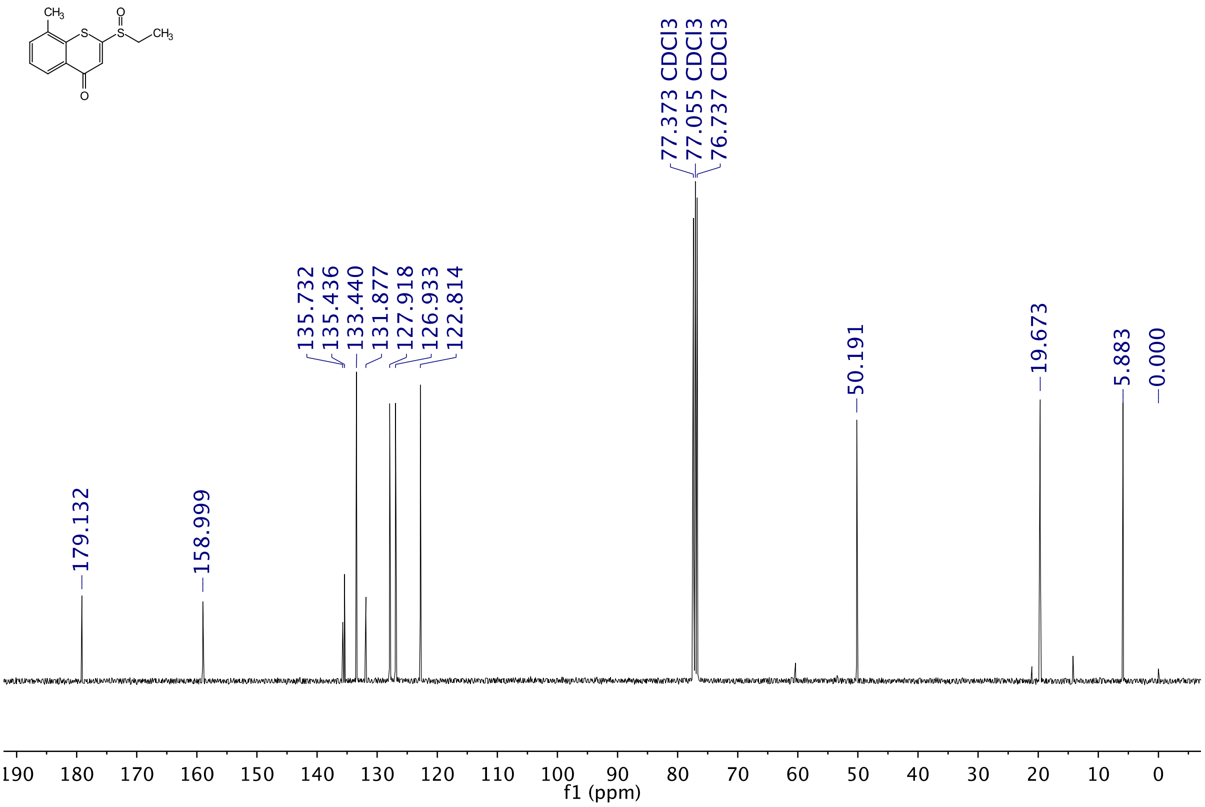

^{1}H and ^{13}C NMR spectra of 7-chloro-2-(ethylsulfinyl)-4*H*-thiochromen-4-one **(2h)**

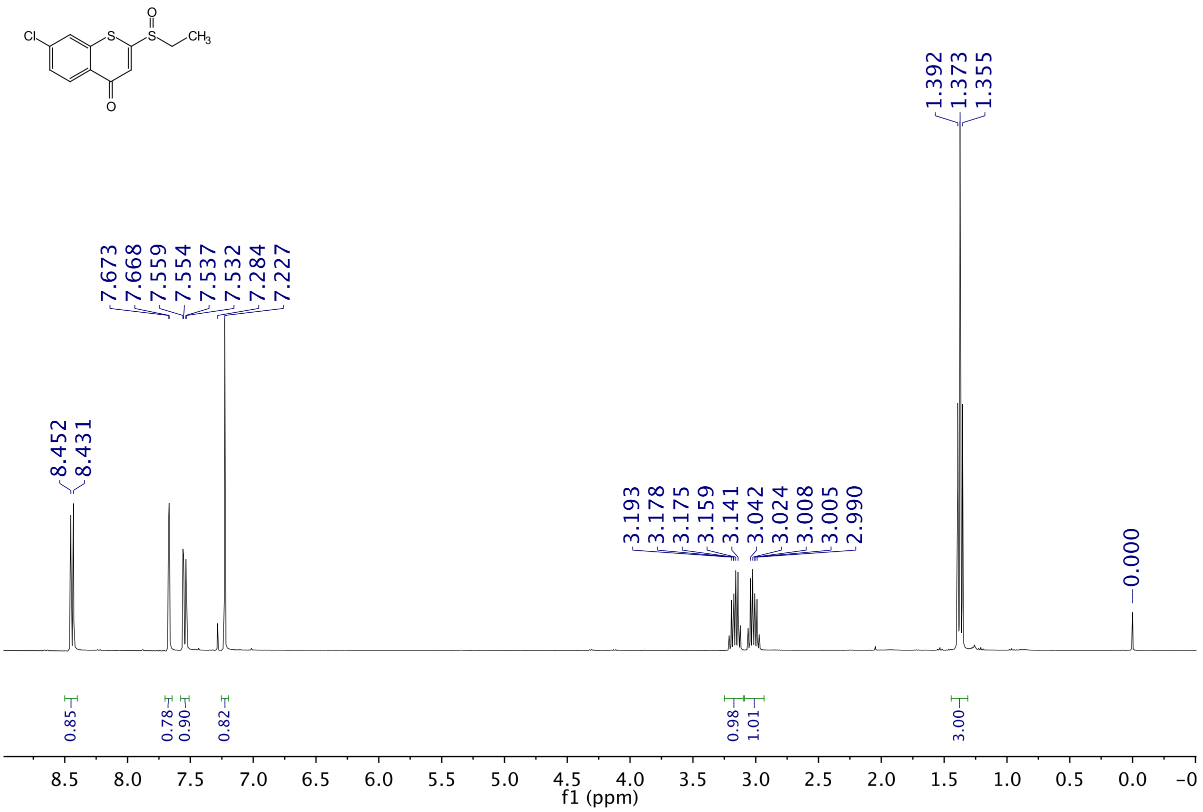

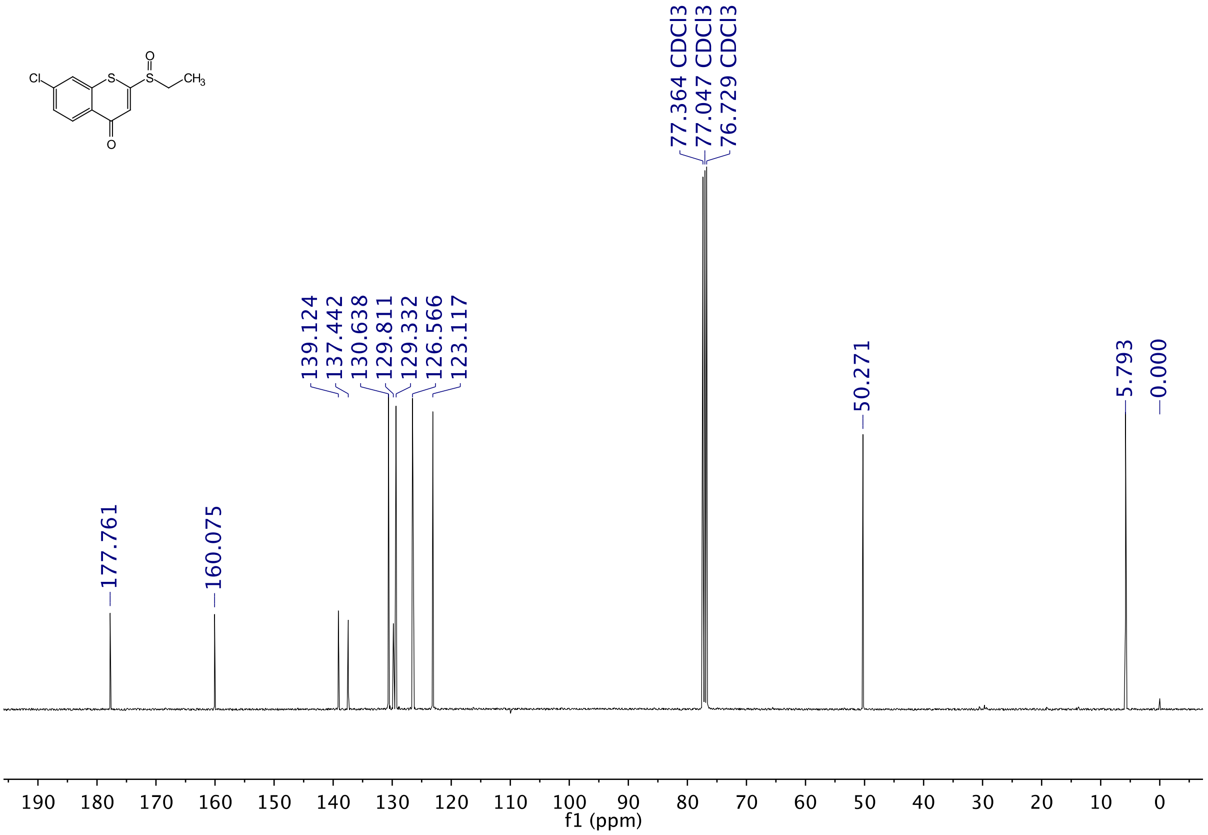

^{1}H and ^{13}C NMR spectra of 2-(ethylsulfonyl)-4*H*-thiochromen-4-one **(3a)**

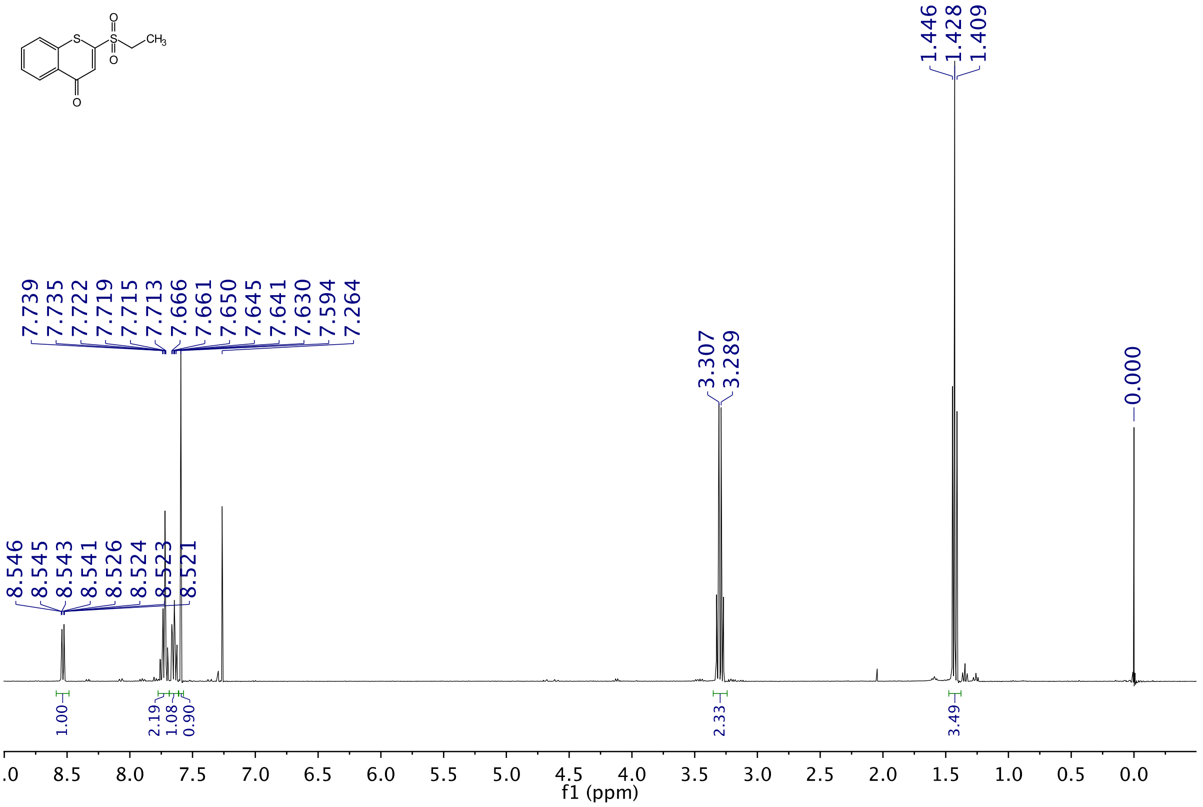

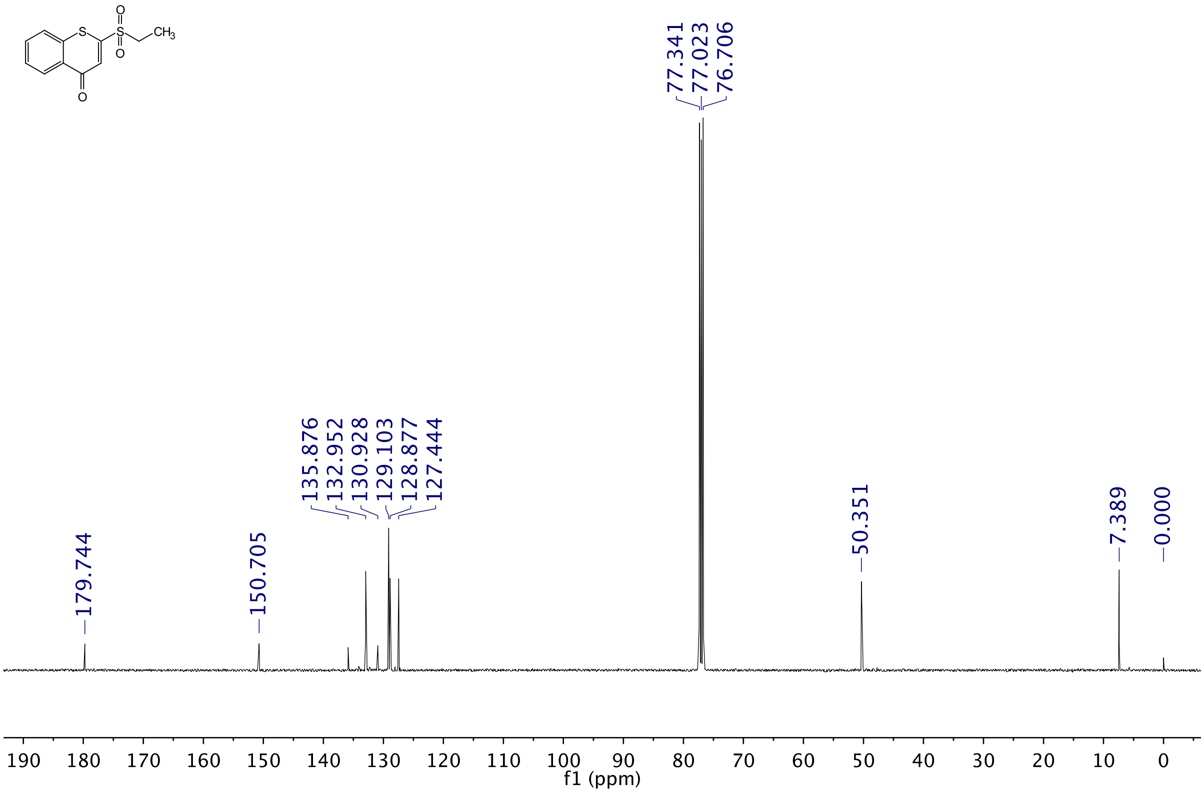

^{1}H and ^{13}C NMR spectra of 2-(4-benzylpiperazin-1-yl)-4*H*-thiochromen-4-one **(4a)**

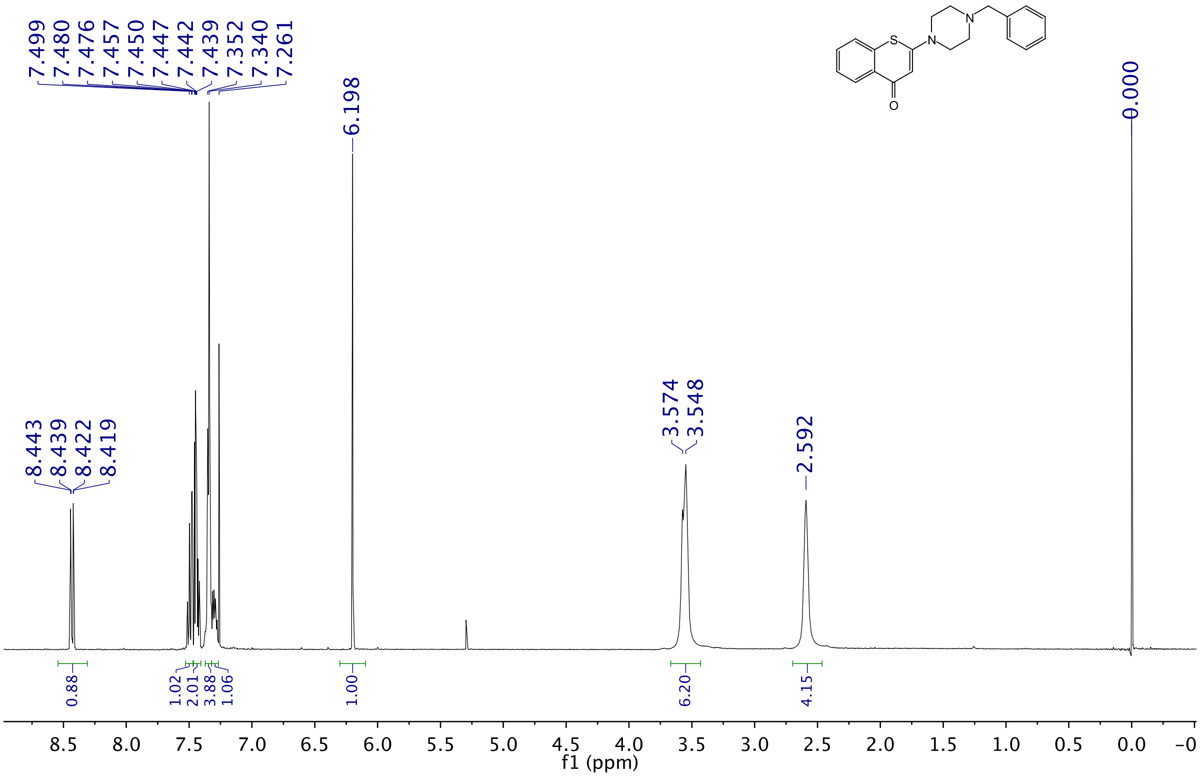

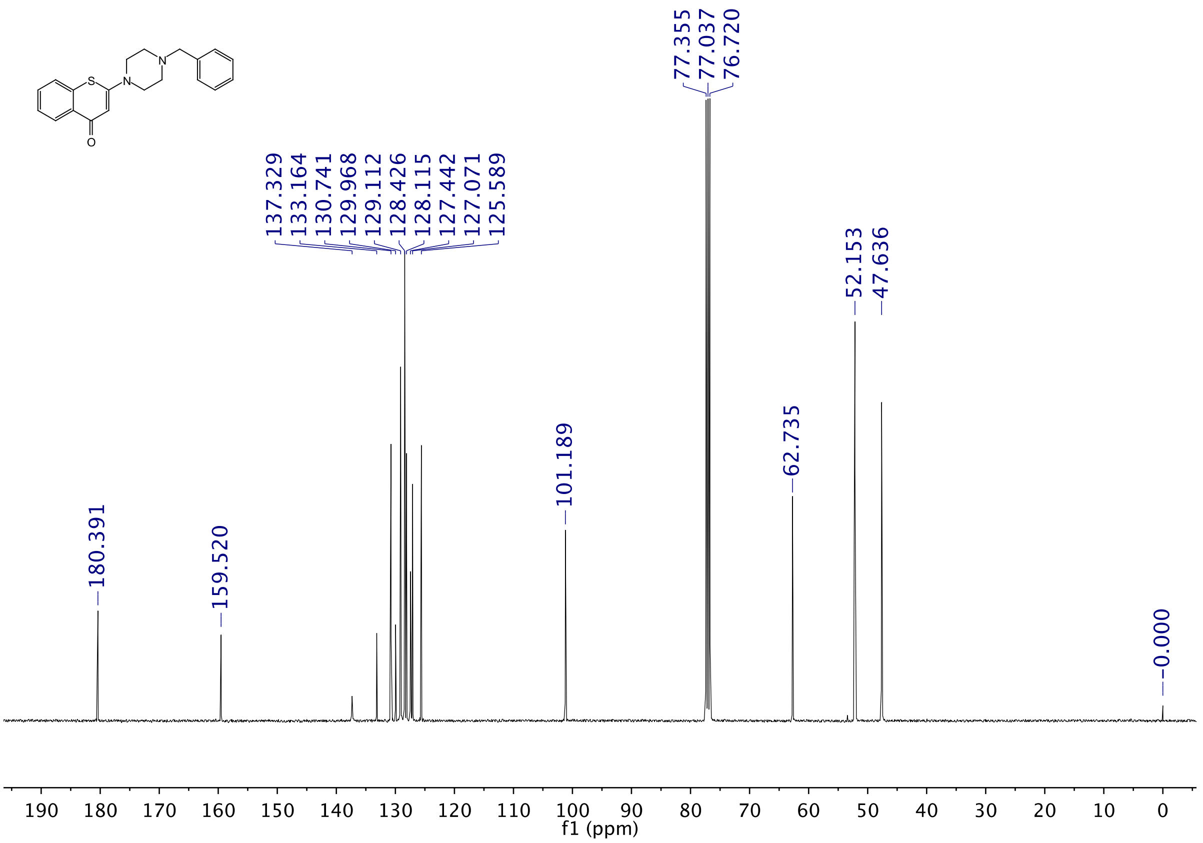

^{1}H and ^{13}C NMR spectra of 2-(phenylamino)-4*H*-thiochromen-4-one **(4b)**

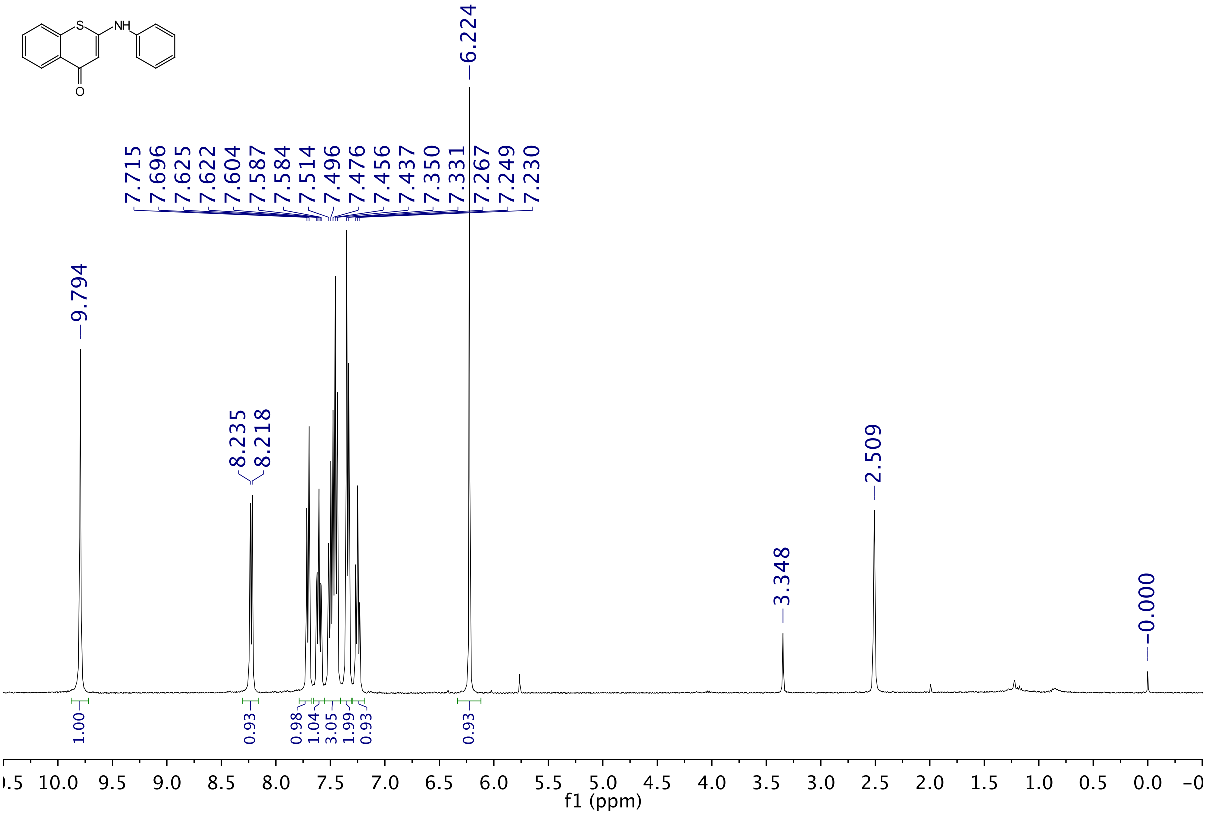

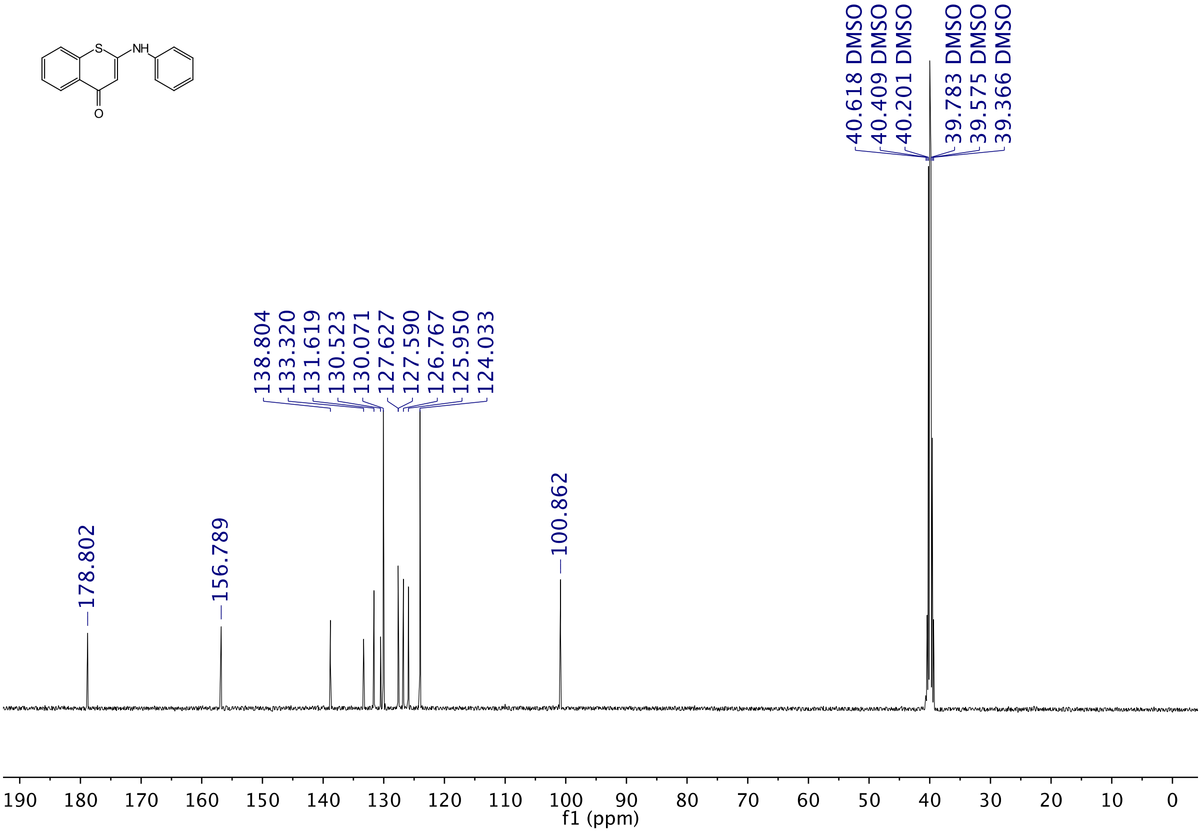

^{1}H and ^{13}C NMR spectra of 2-(benzylamino)-4*H*-thiochromen-4-one **(4c)**

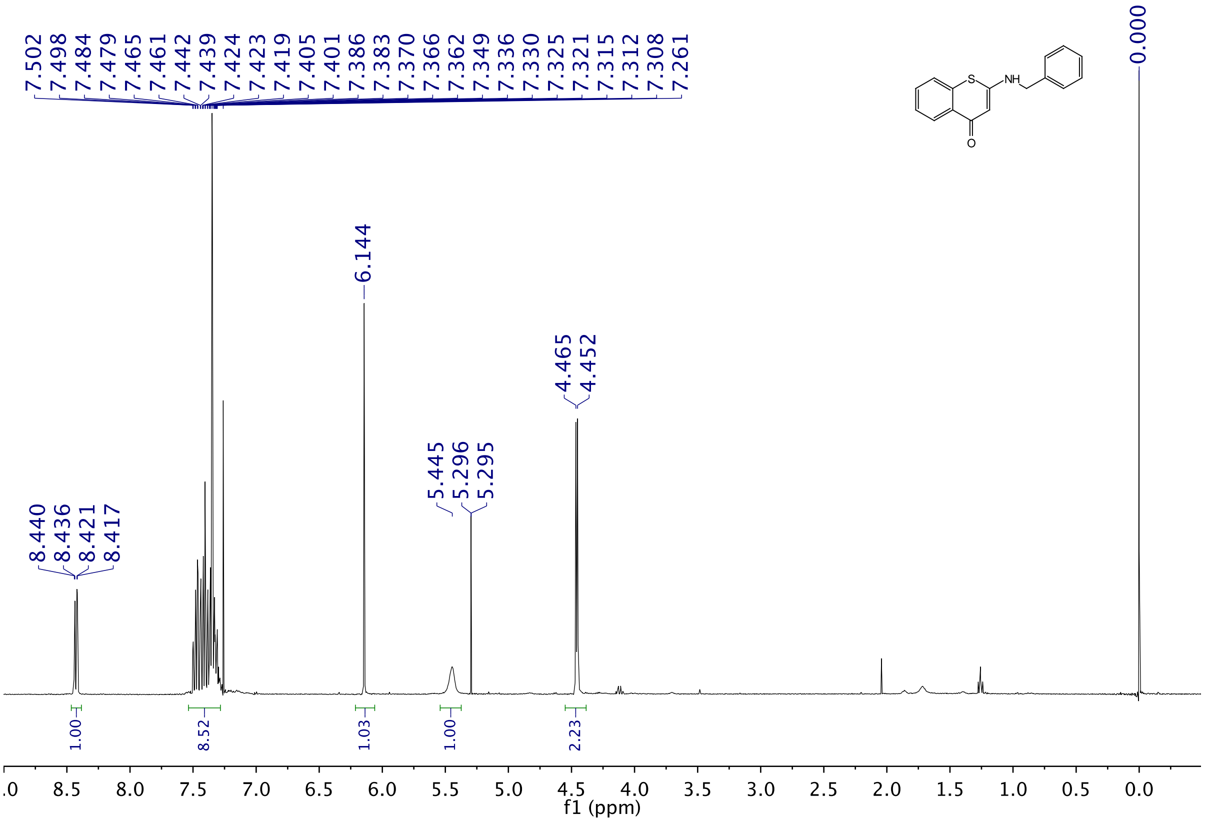

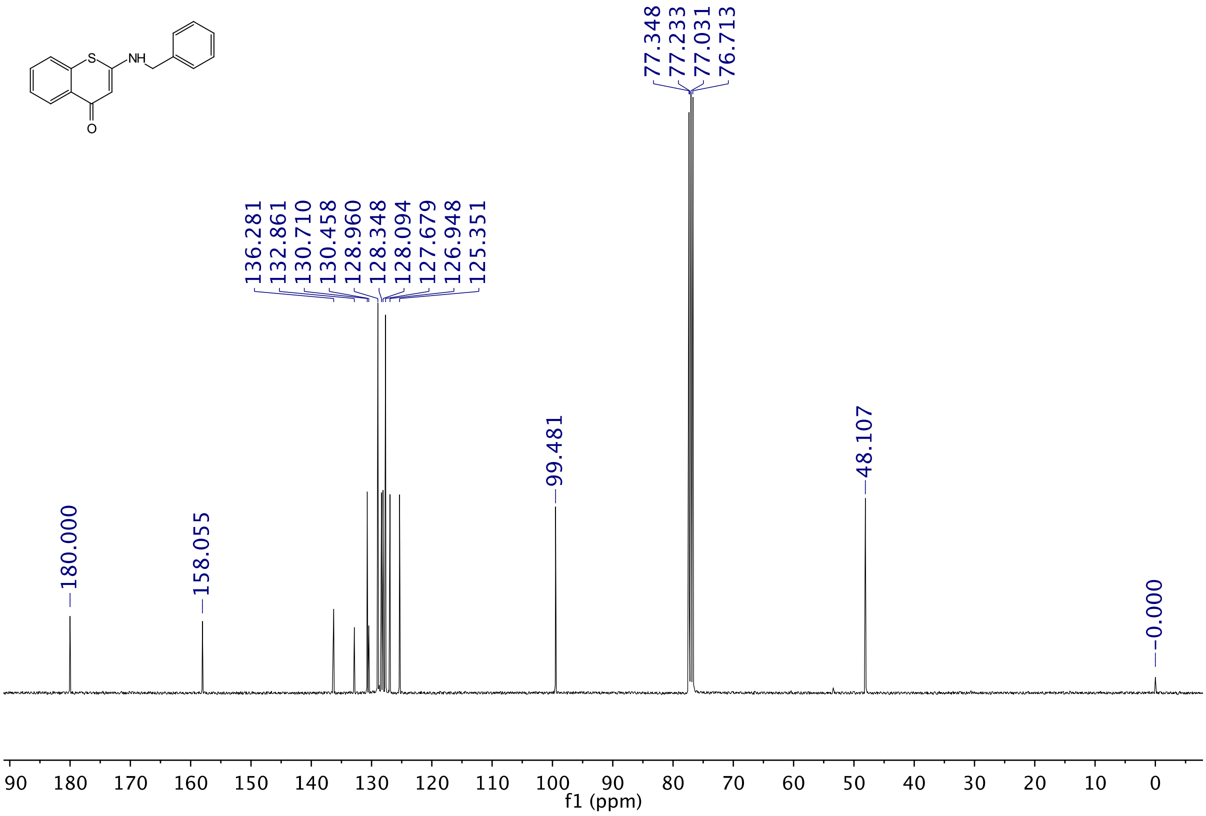

^{1}H and ^{13}C NMR spectra of 2-(propylamino)-4*H*-thiochromen-4-one **(4d)**

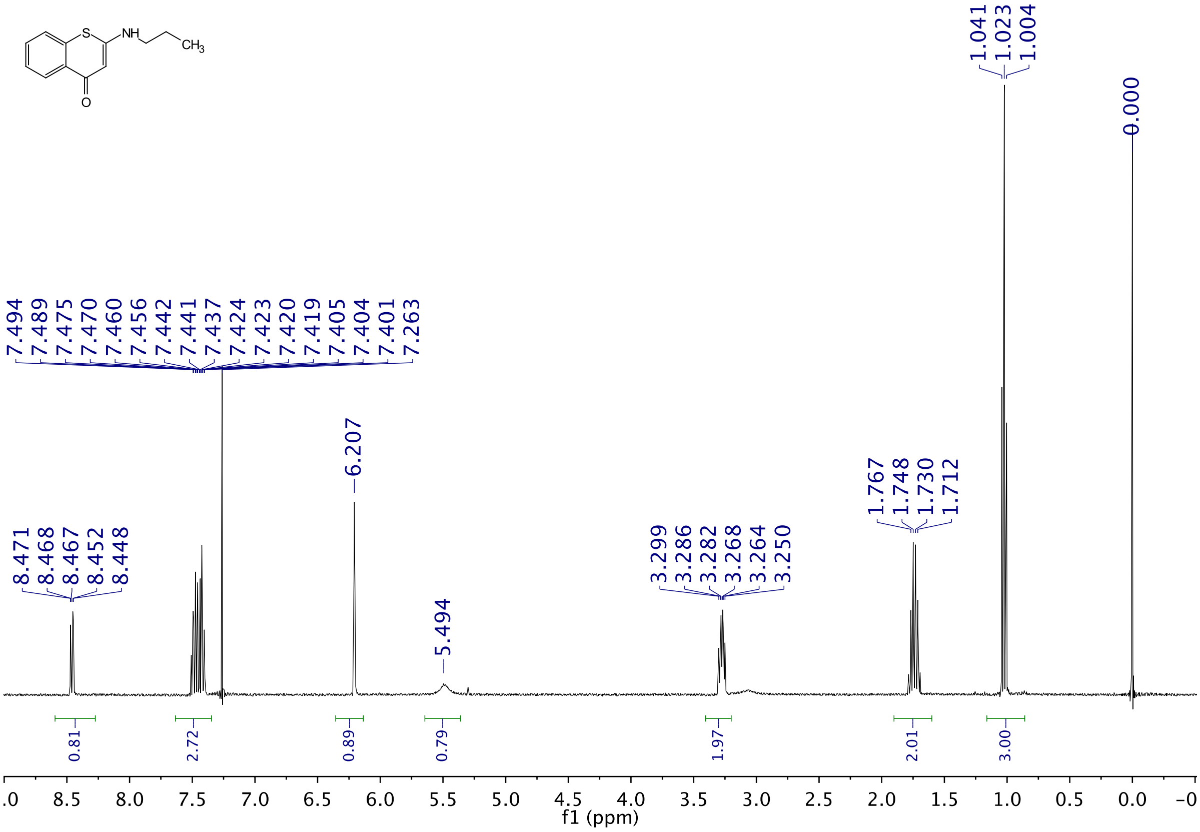

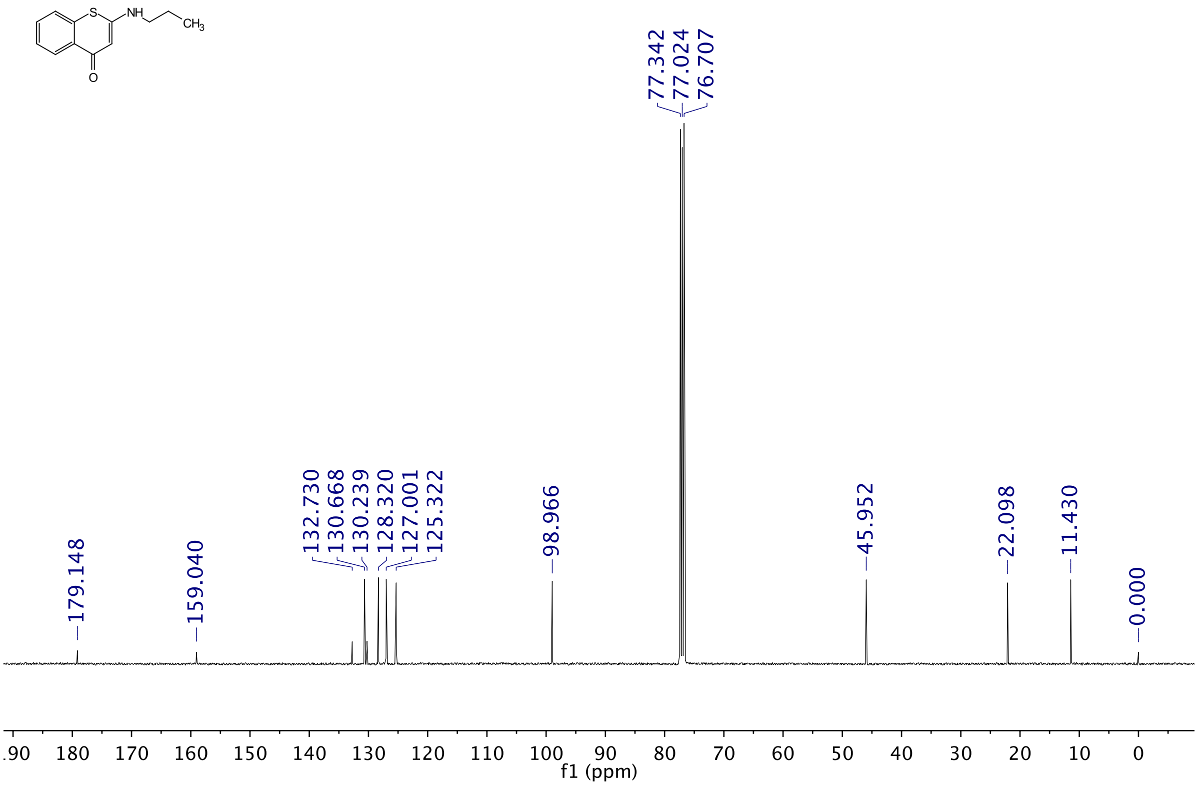

^{1}H and ^{13}C NMR spectra of 2-((4-methoxyphenyl)amino)-4*H*-thiochromen-4-one **(4e)**

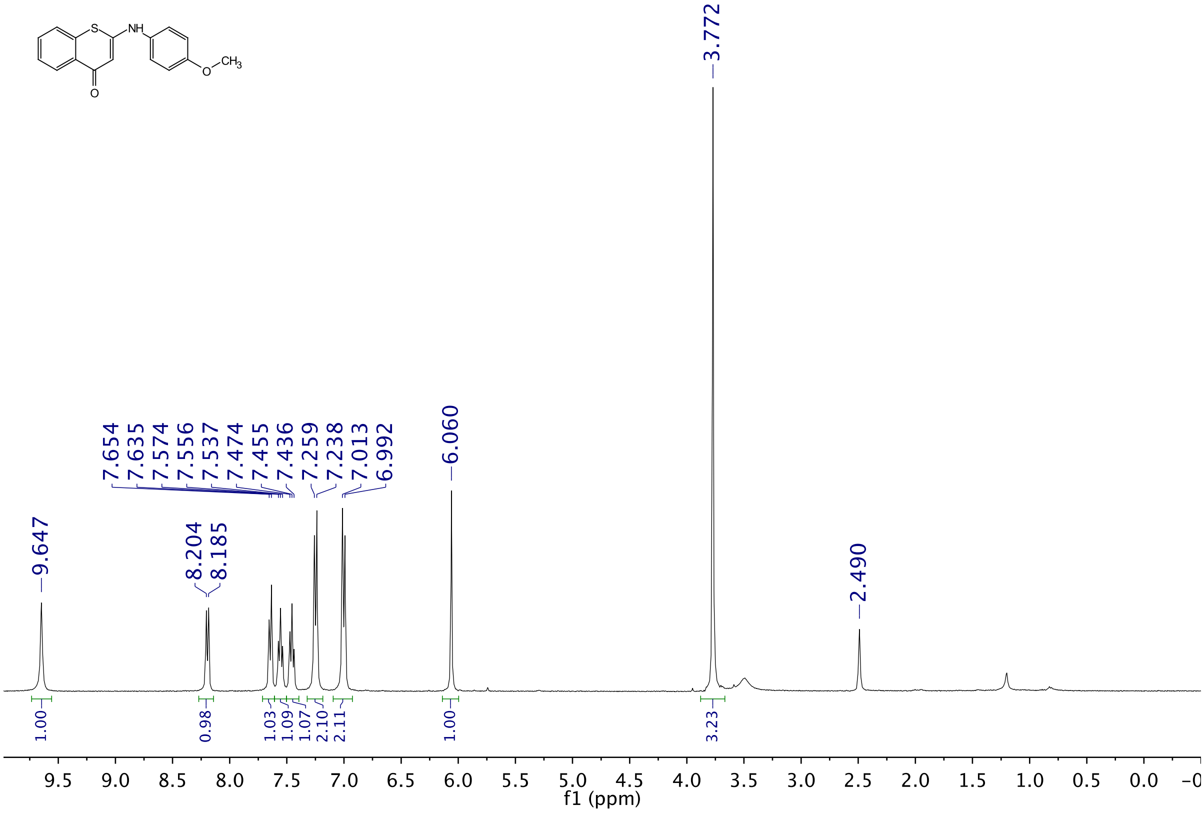

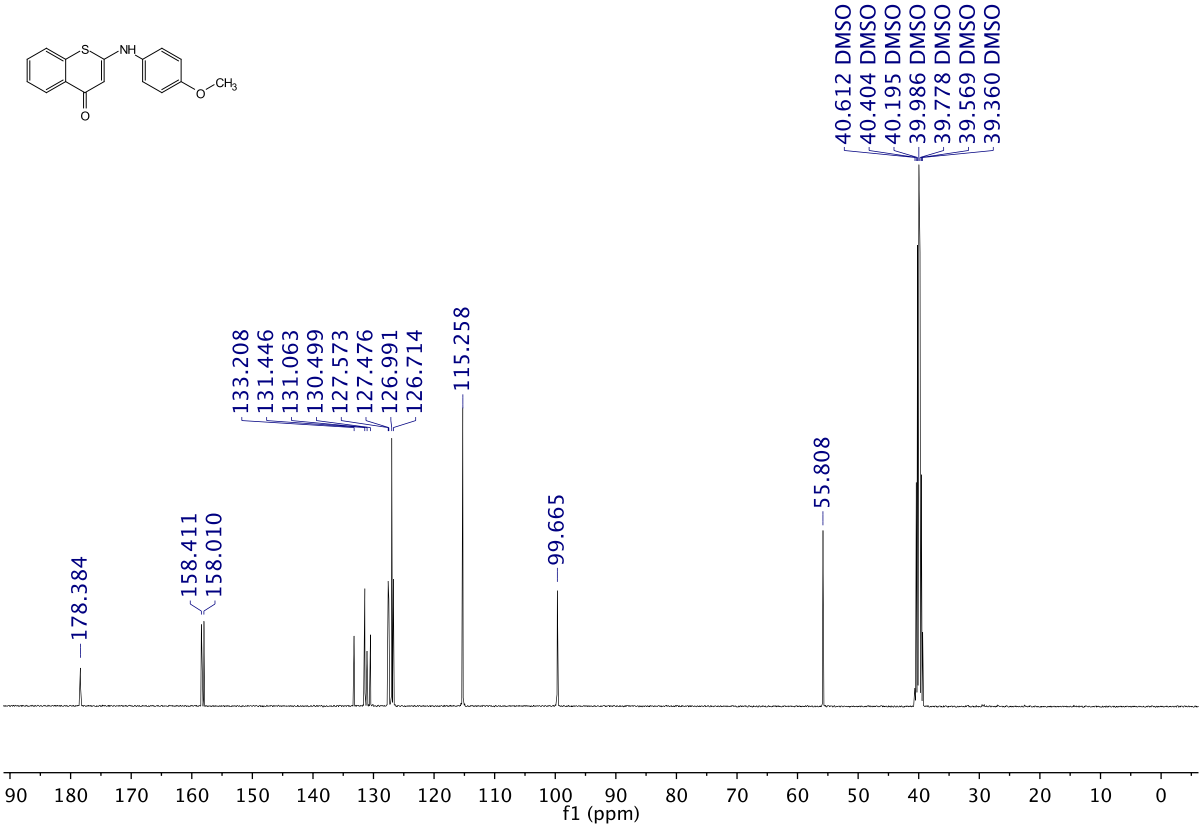

^{1}H and ^{13}C NMR spectra of 2-((3,4-dimethoxybenzyl)amino)-4*H*-thiochromen-4-one **(4f)**

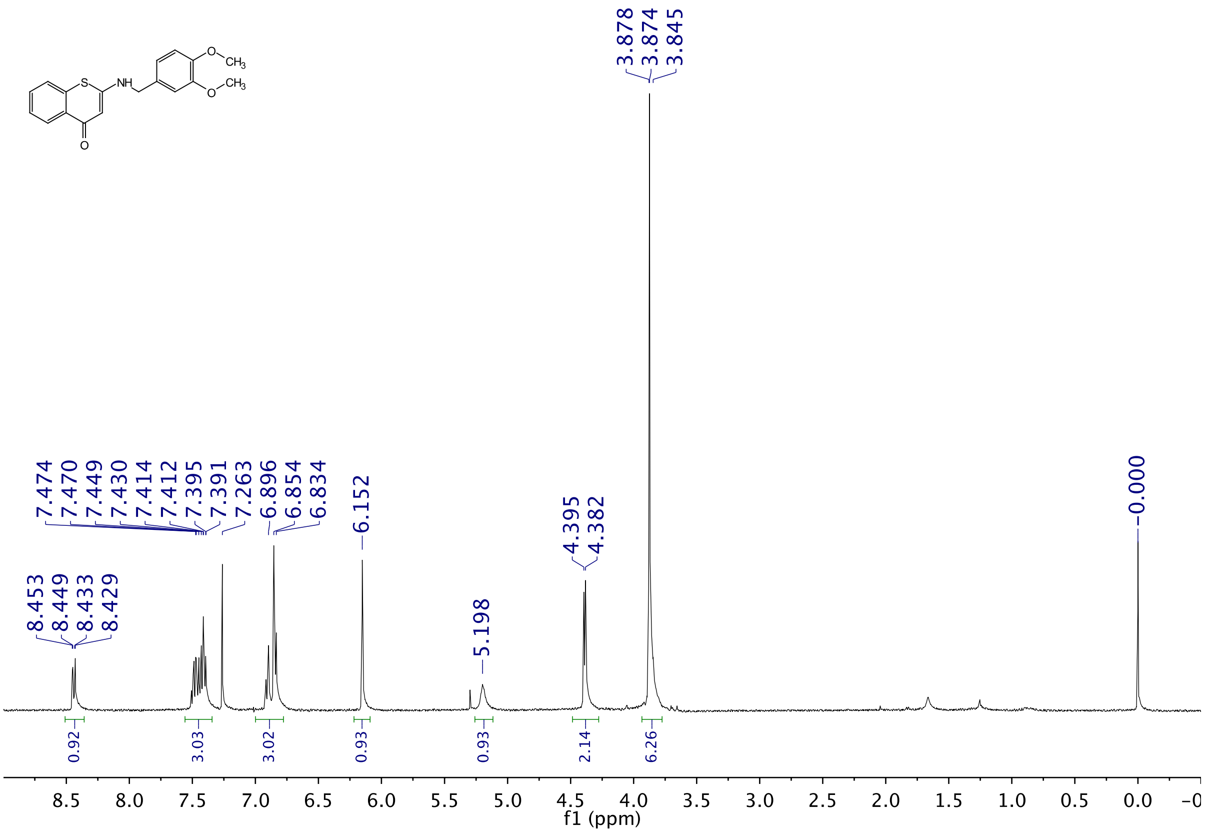

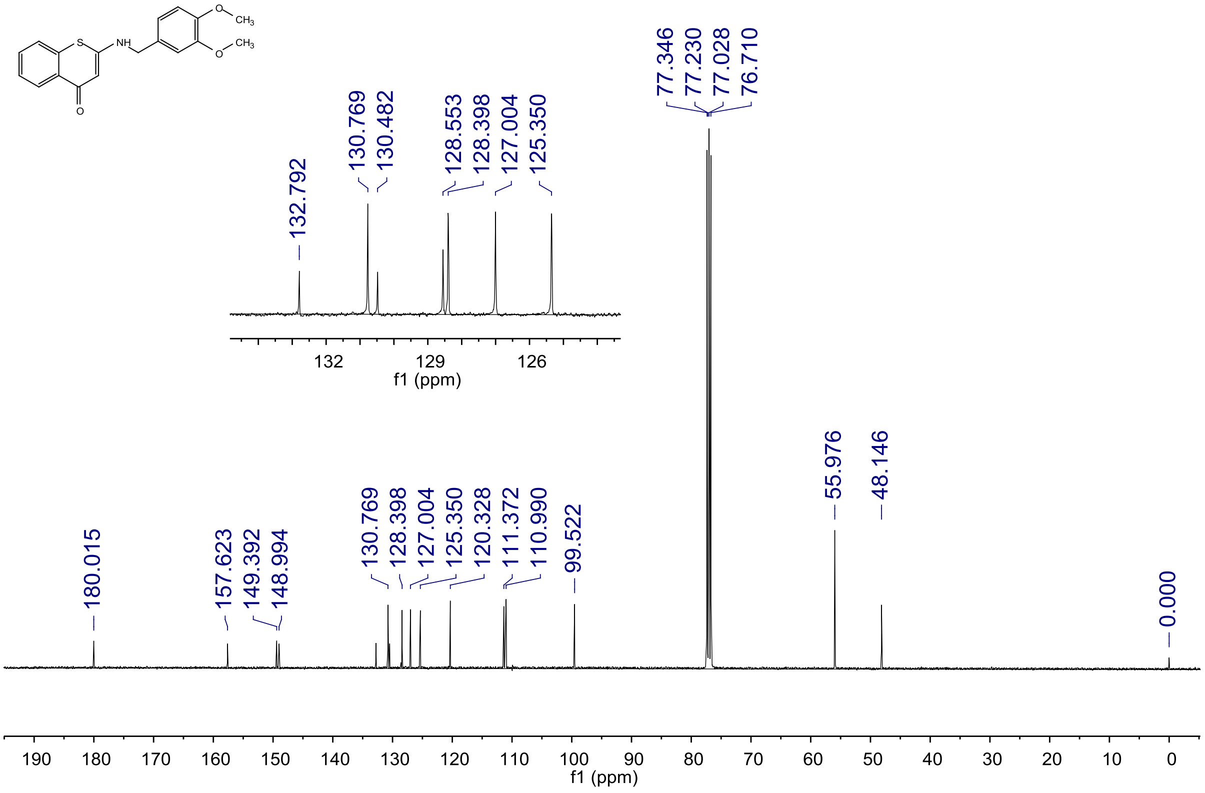

^{1}H and ^{13}C NMR spectra of 2-((cyclohexylmethyl)amino)-4*H*-thiochromen-4-one **(4g)**

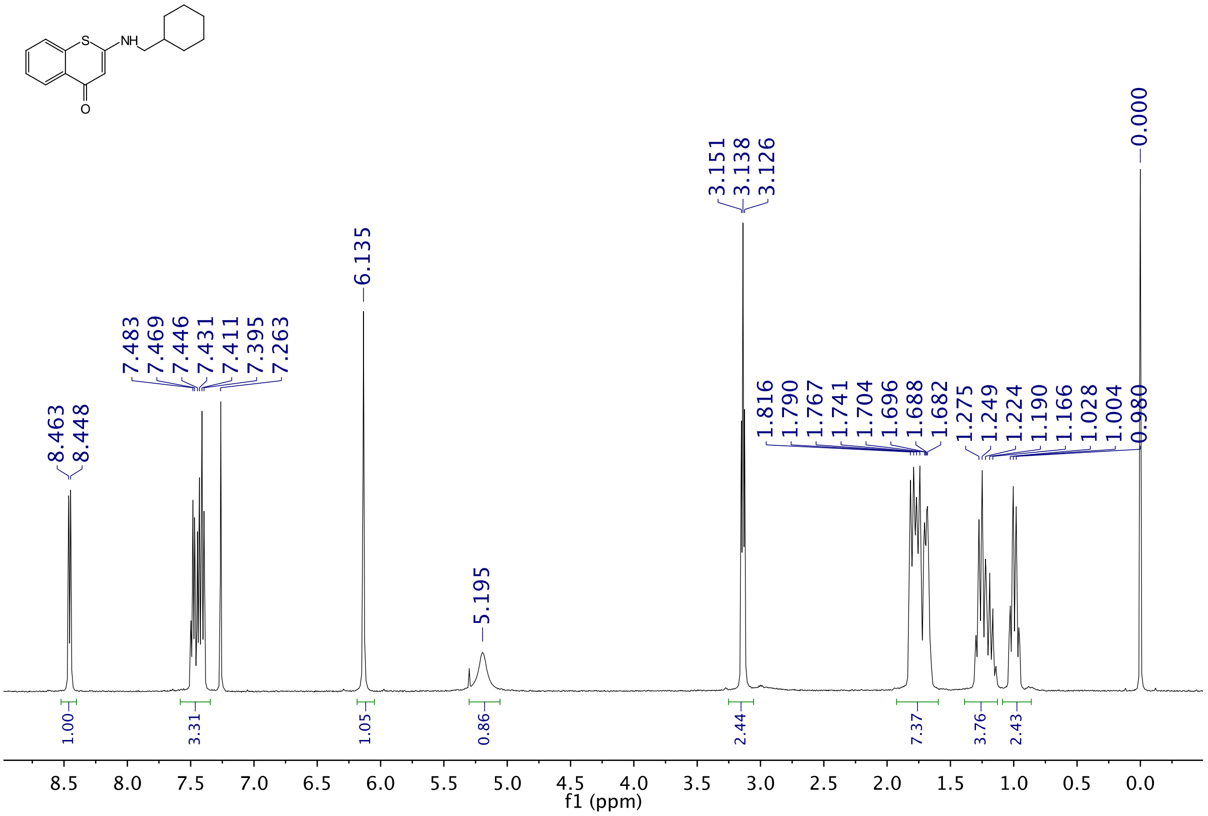

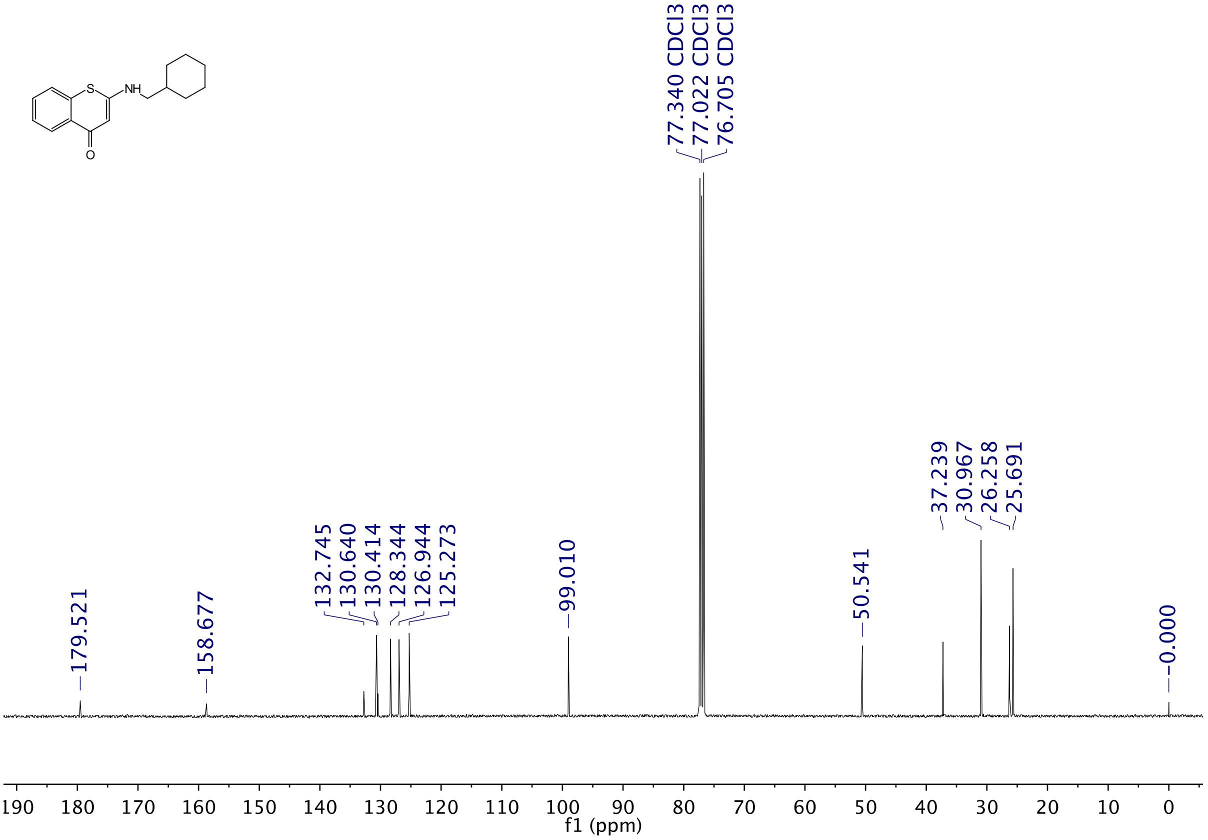

^{1}H and ^{13}C NMR spectra of 2-(cyclohexylamino)-4*H*-thiochromen-4-one **(4h)**

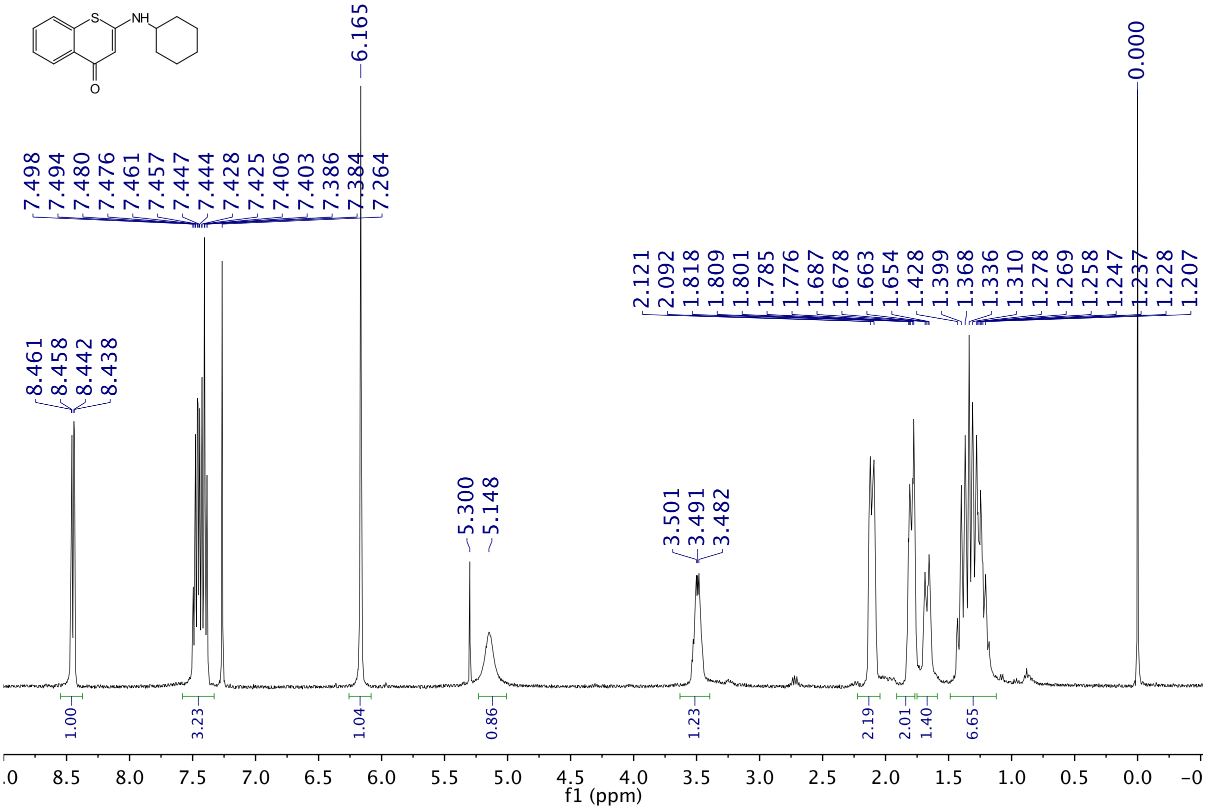

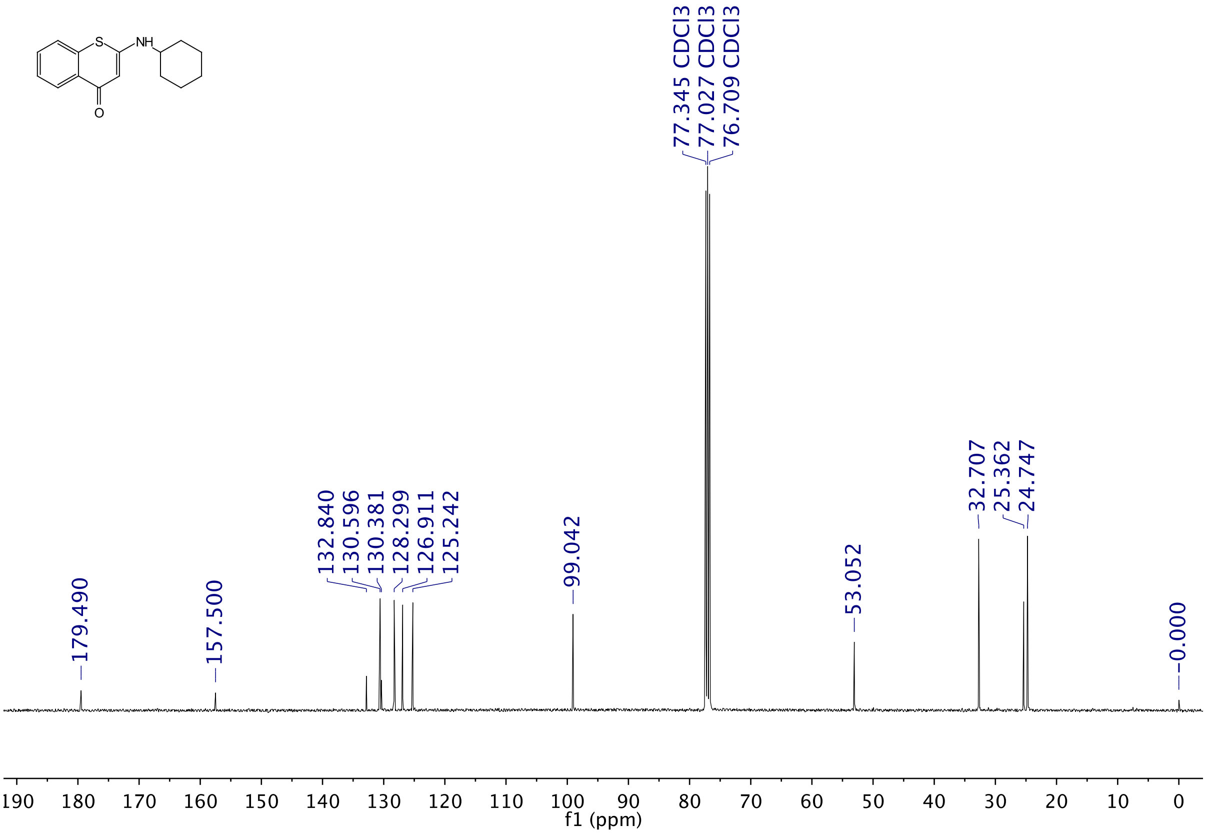

^{1}H and ^{13}C NMR spectra of 2-(diethylamino)-4*H*-thiochromen-4-one **(4i)**

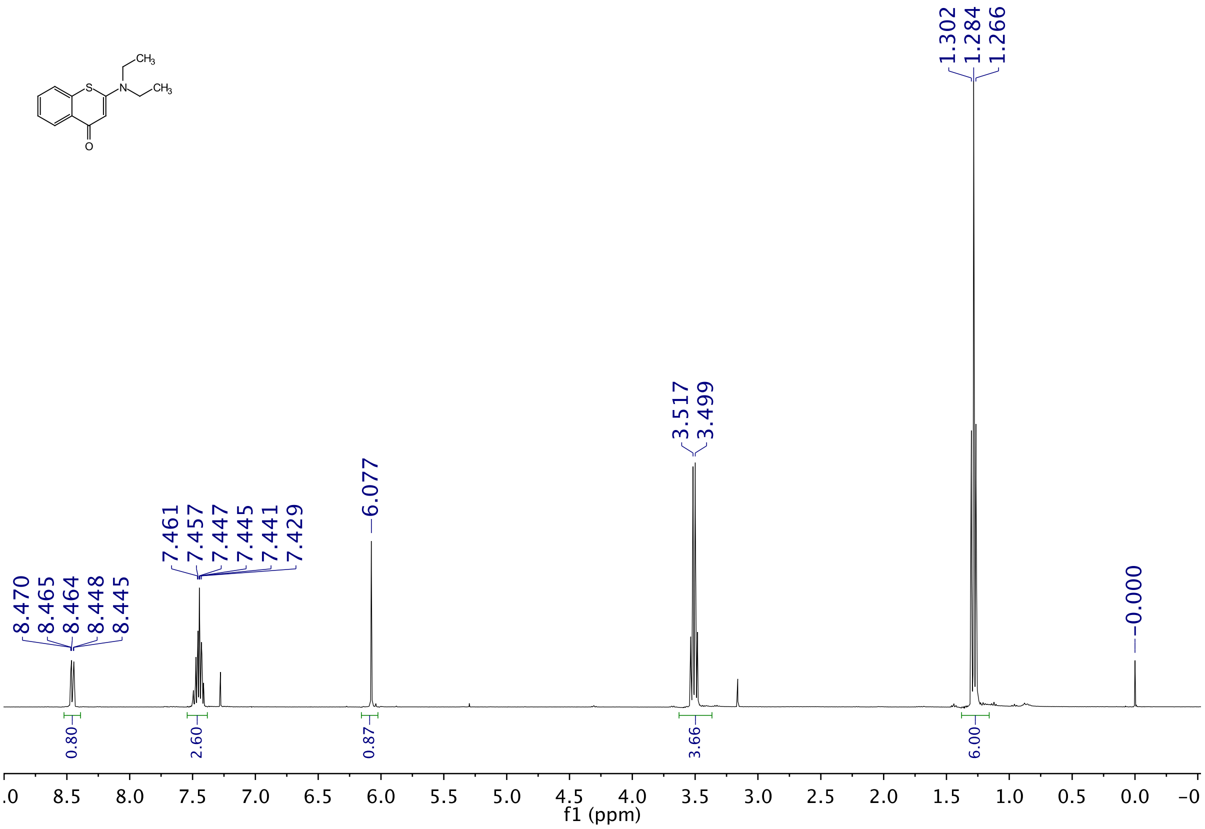

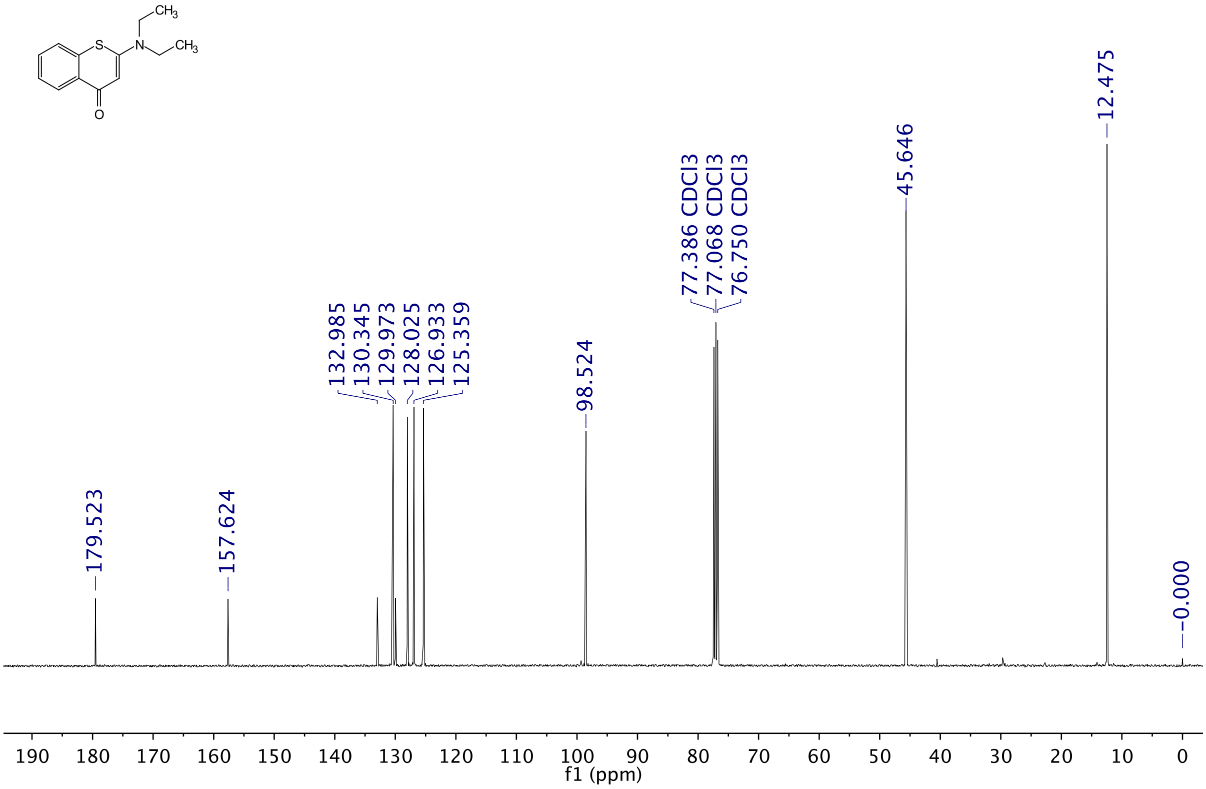

^{1}H and ^{13}C NMR spectra of 2-(4-benzylpiperazin-1-yl)-5-chloro-4*H*-thiochromen-4-one **(4j)**

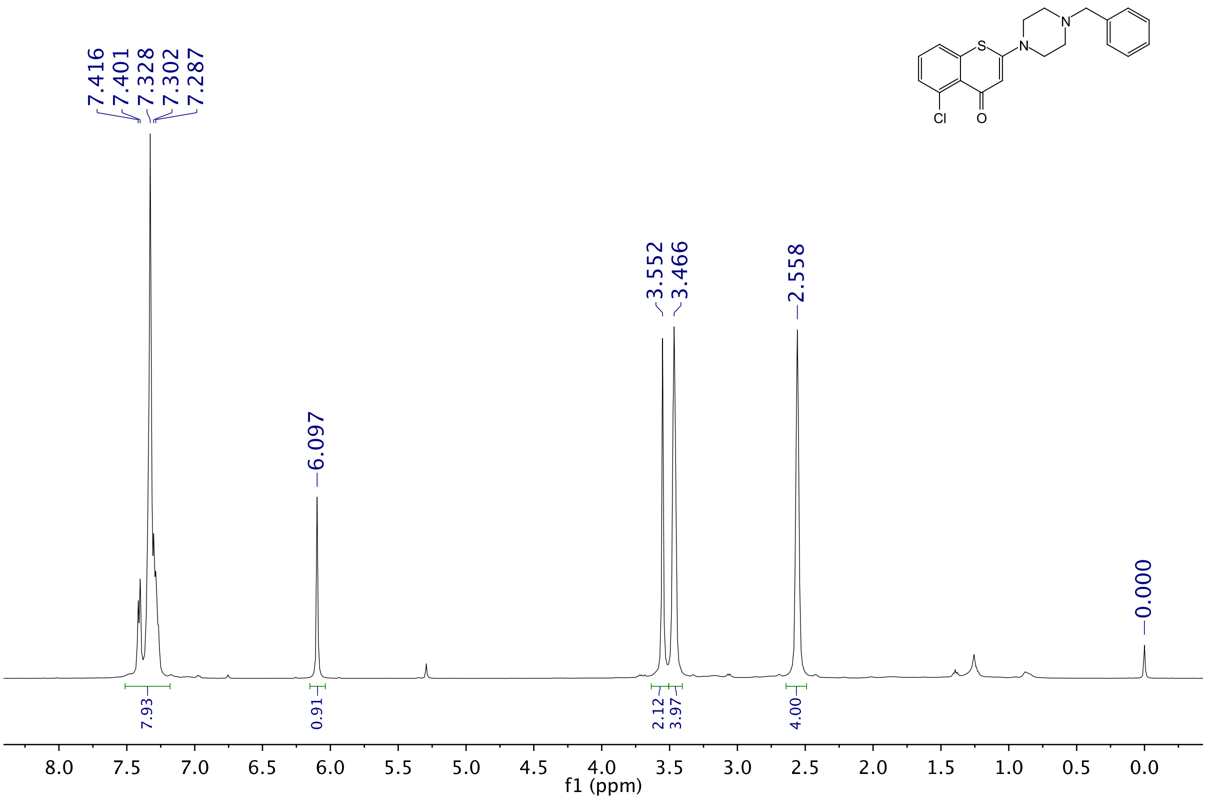

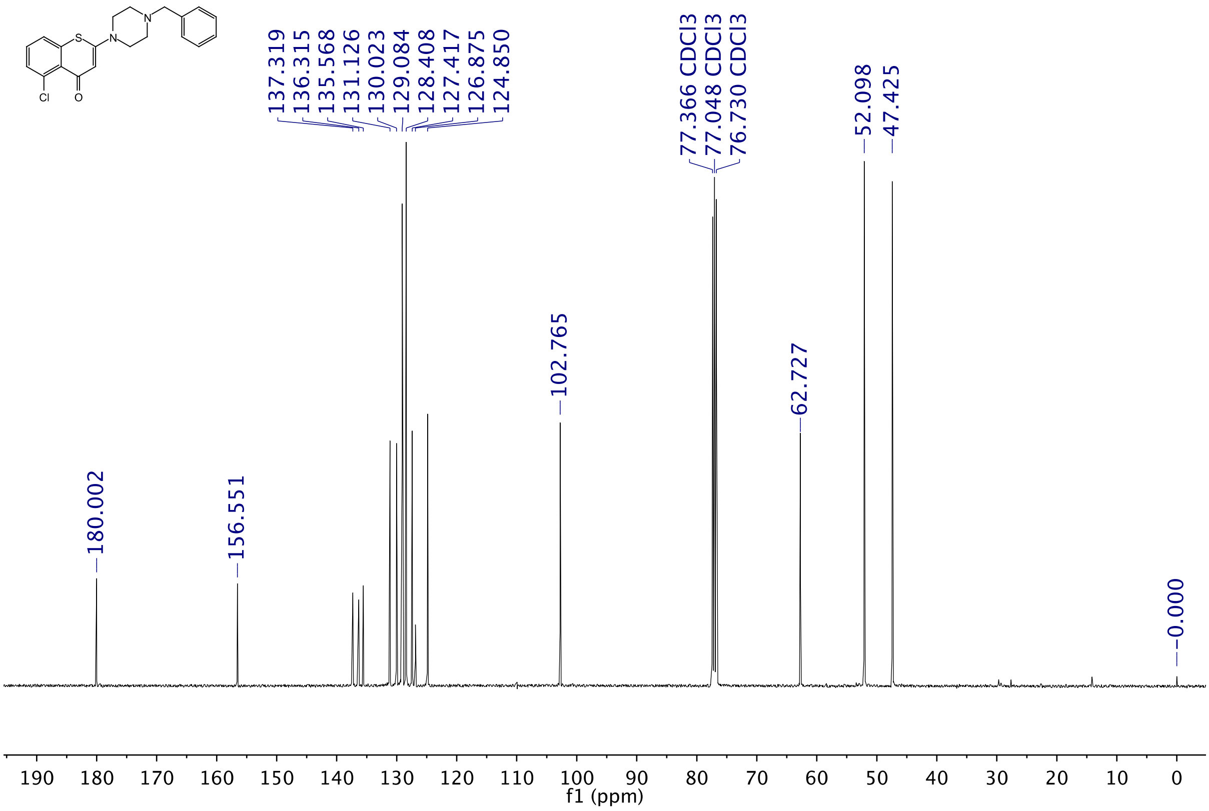

^{1}H and ^{13}C NMR spectra of 2-(4-benzylpiperazin-1-yl)-7-fluoro-4*H*-thiochromen-4-one **(4k)**

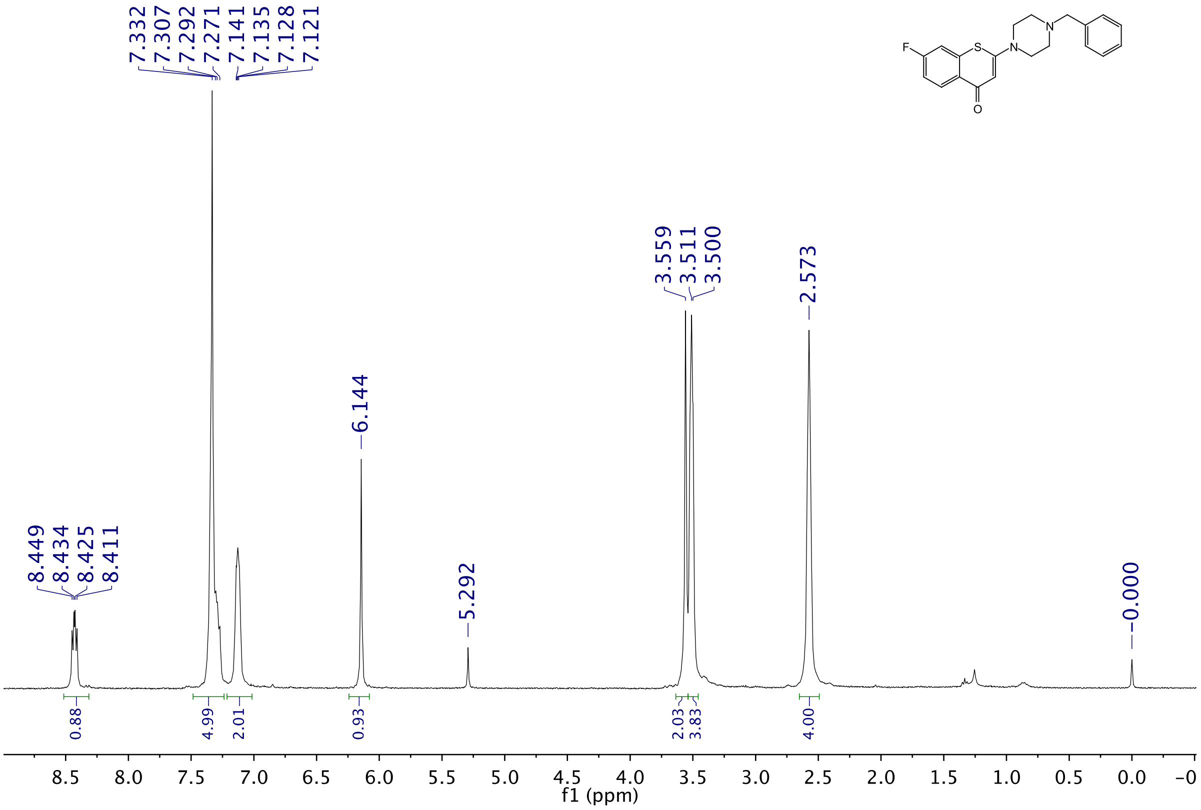

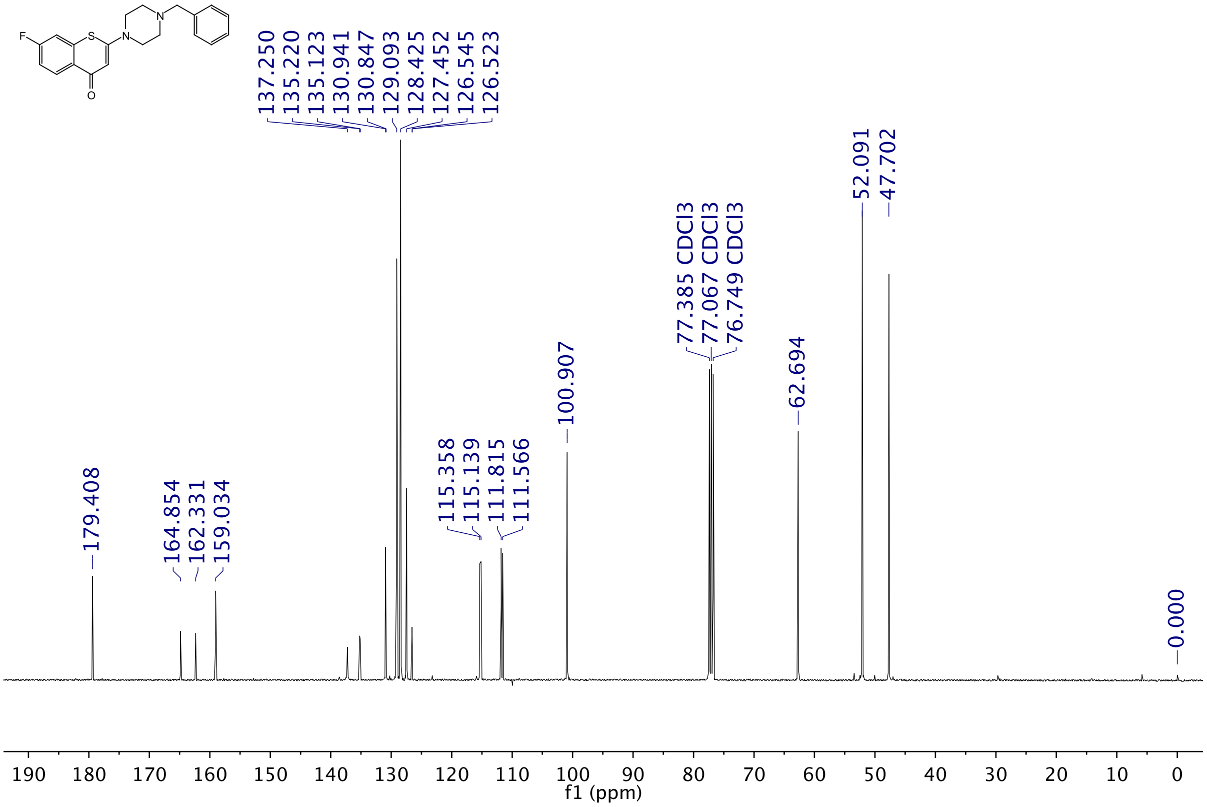

^{1}H and ^{13}C NMR spectra of 2-(4-benzylpiperazin-1-yl)-7,8-dichloro-4*H*-thiochromen-4-one **(4l)**

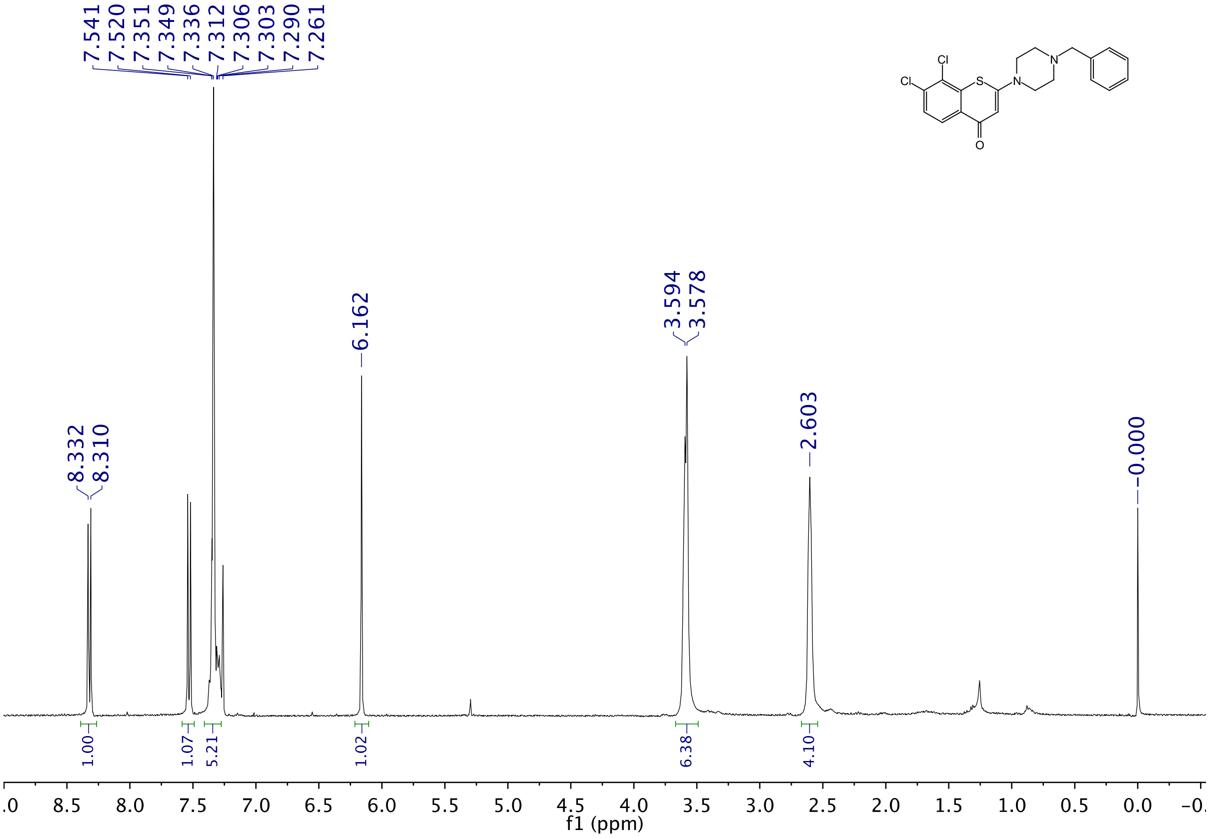

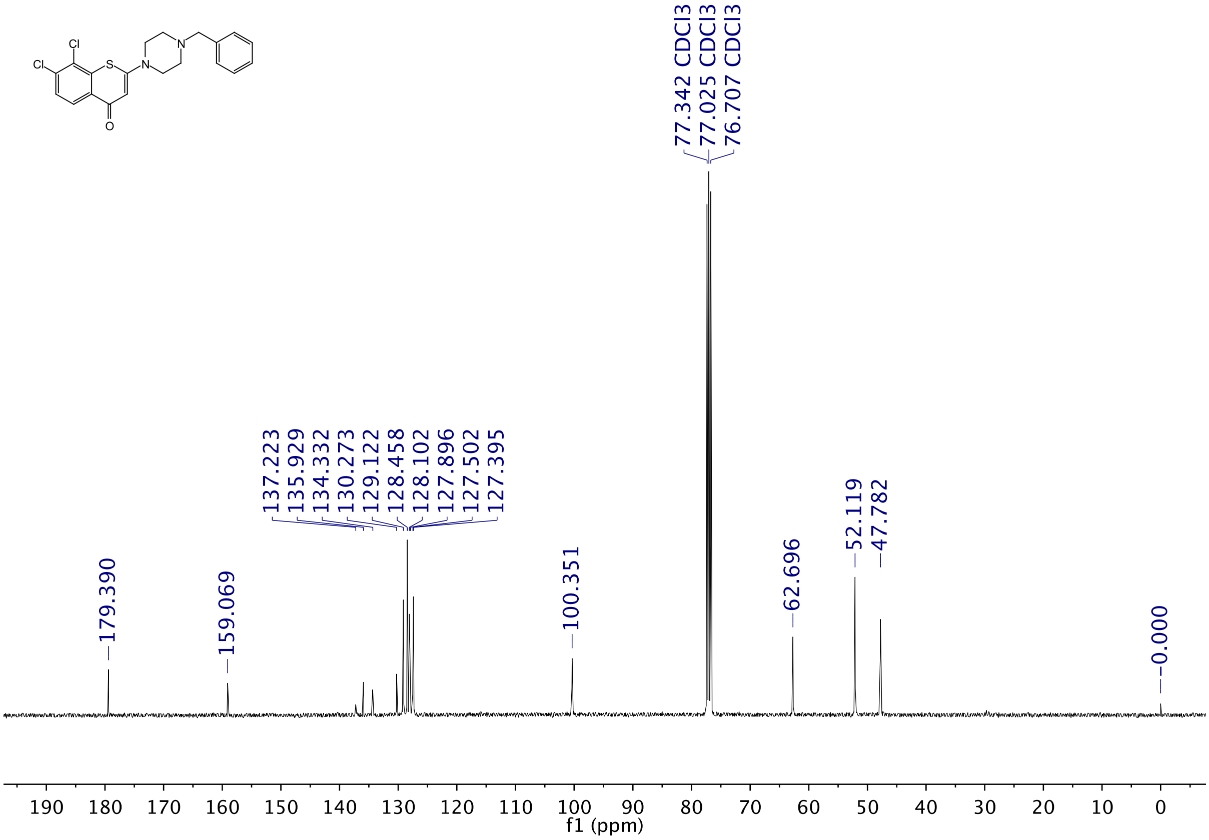

^{1}H and ^{13}C NMR spectra of 2-(4-benzylpiperazin-1-yl)-6-(trifluoromethyl)-4*H*-thiochromen-4-one **(4m)**

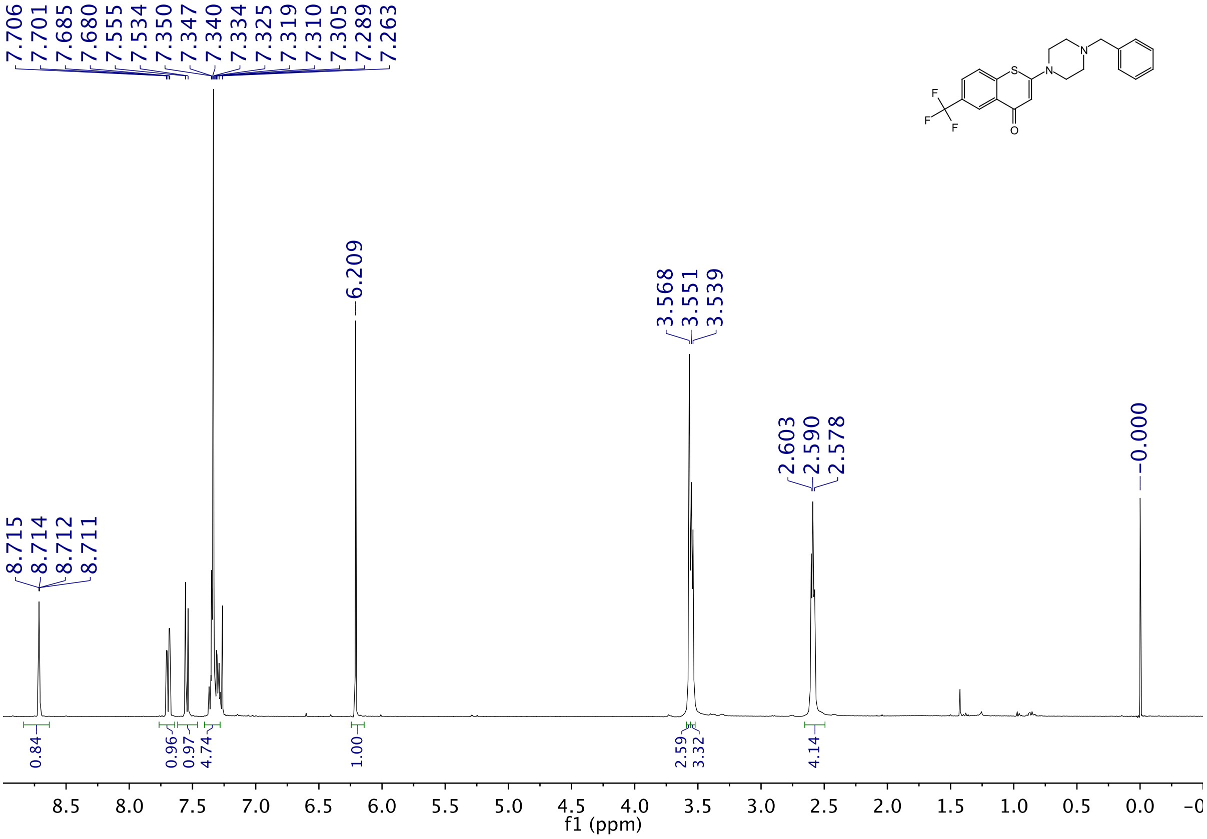

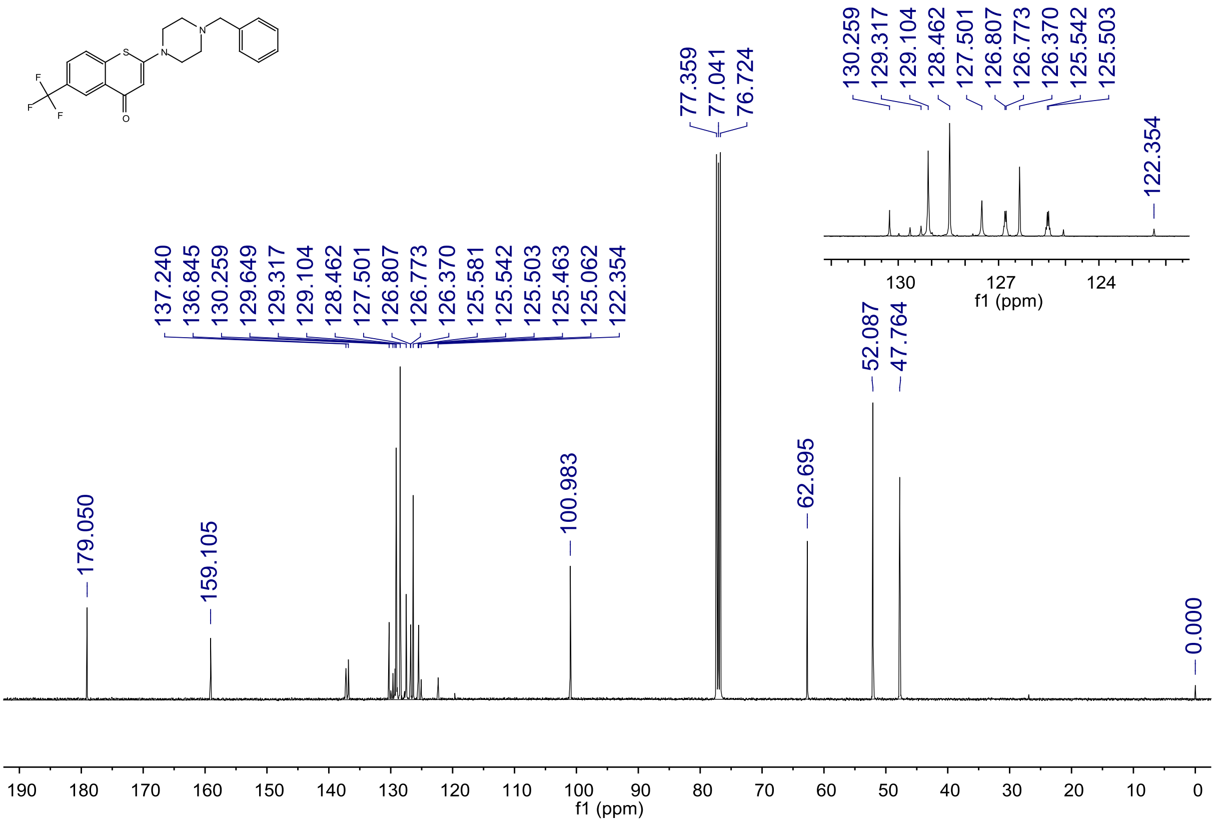

^{1}H and ^{13}C NMR spectra of 2-(4-benzylpiperazin-1-yl)-8-iodo-6-(trifluoromethyl)-4*H*-thiochromen-4-one **(4n)**

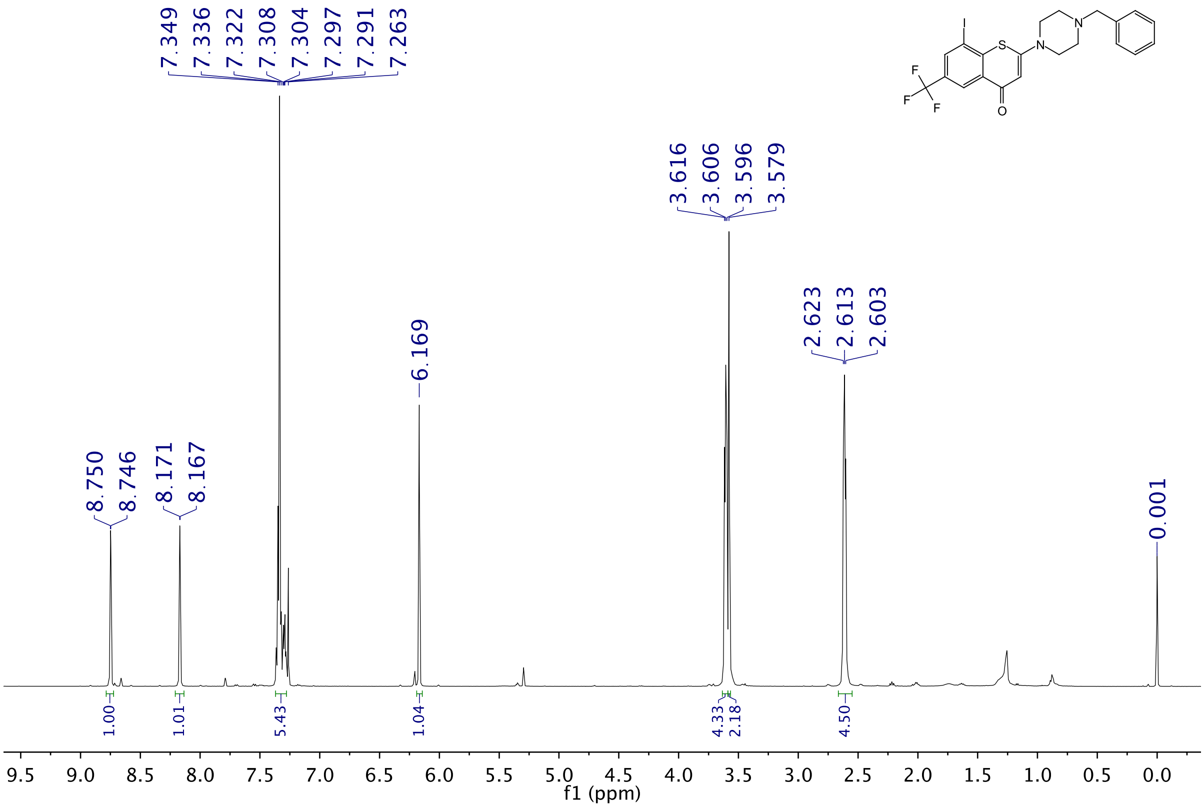

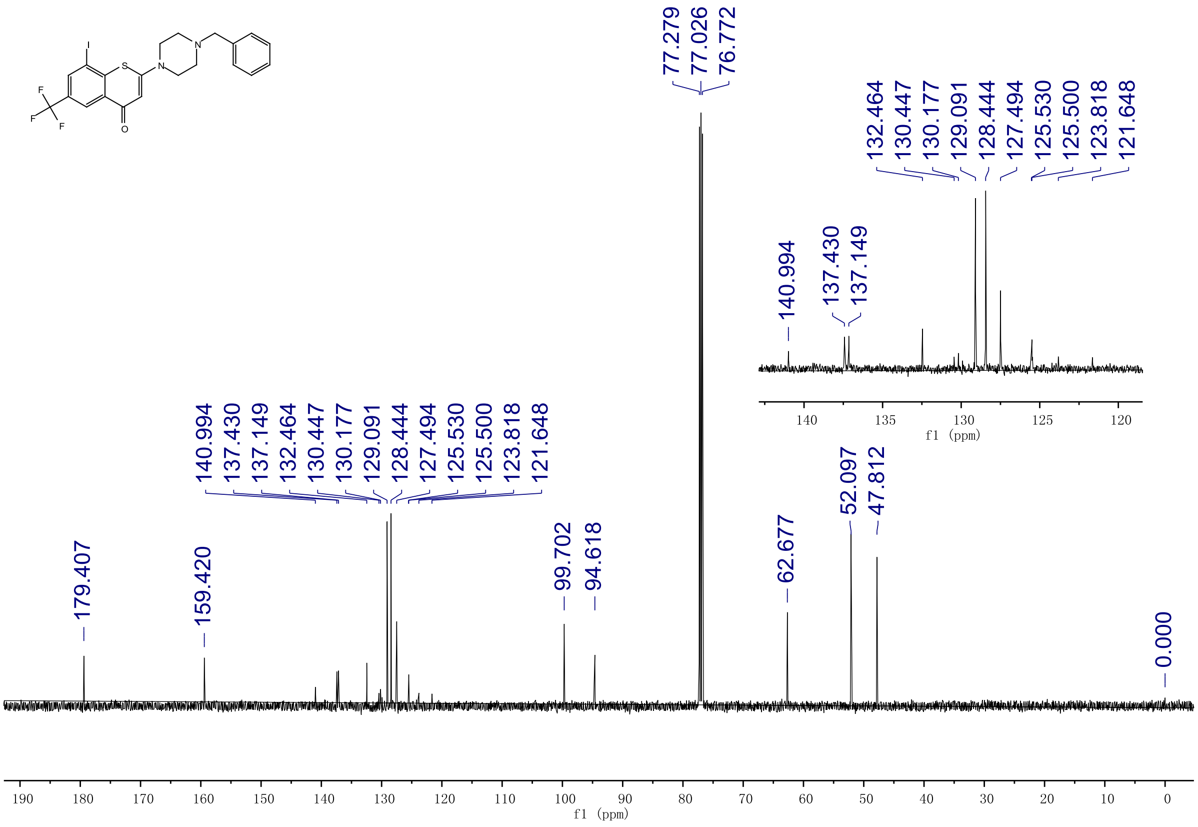

^{1}H and ^{13}C NMR spectra of 2-(4-benzylpiperazin-1-yl)-8-methyl-4*H*-thiochromen-4-one **(4o)**

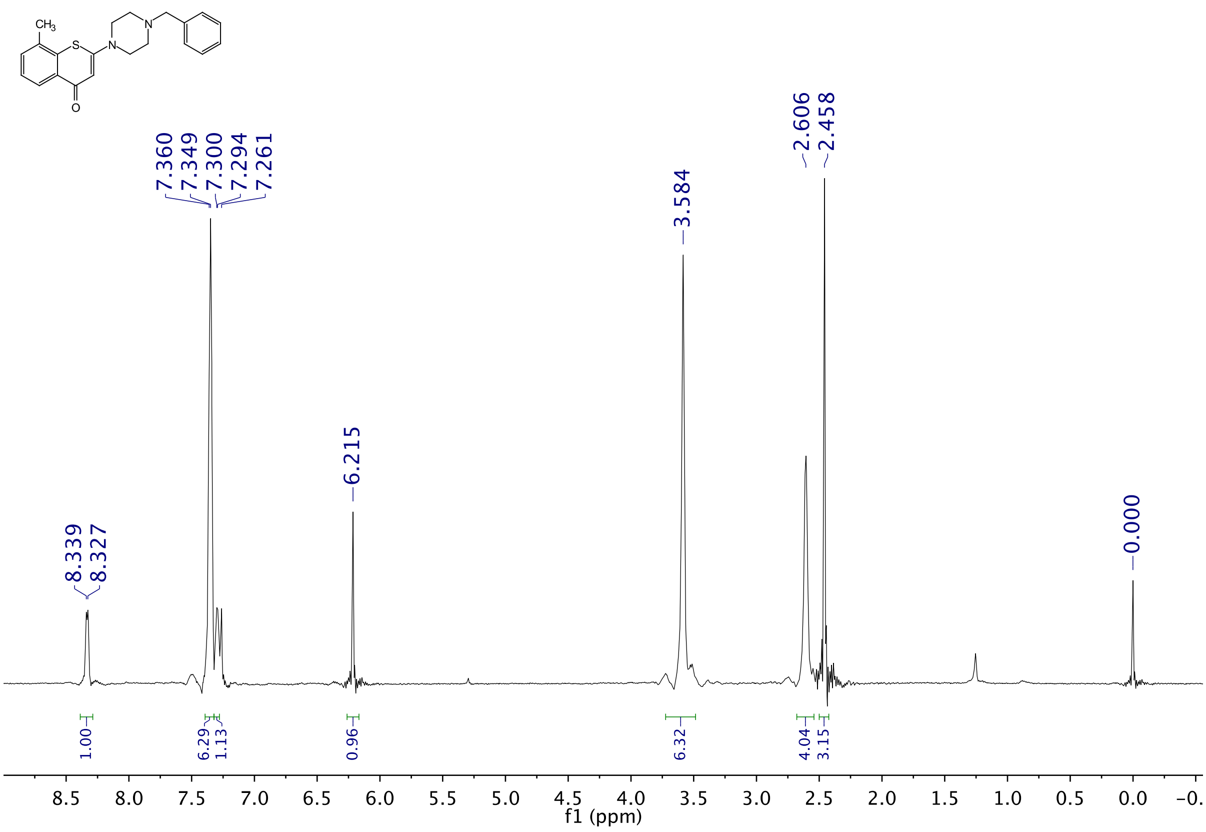

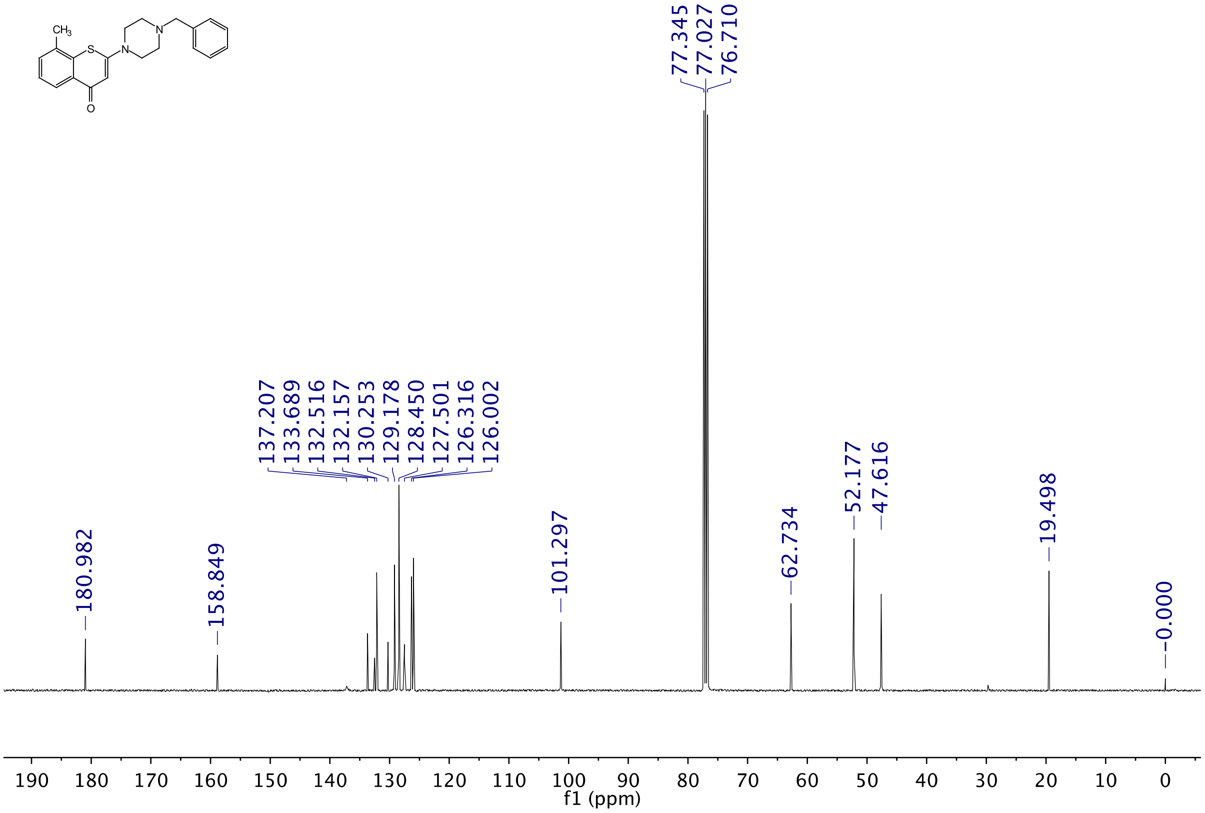

^{1}H and ^{13}C NMR spectra of 2-(benzylamino)-8-methyl-4*H*-thiochromen-4-one **(4p)**

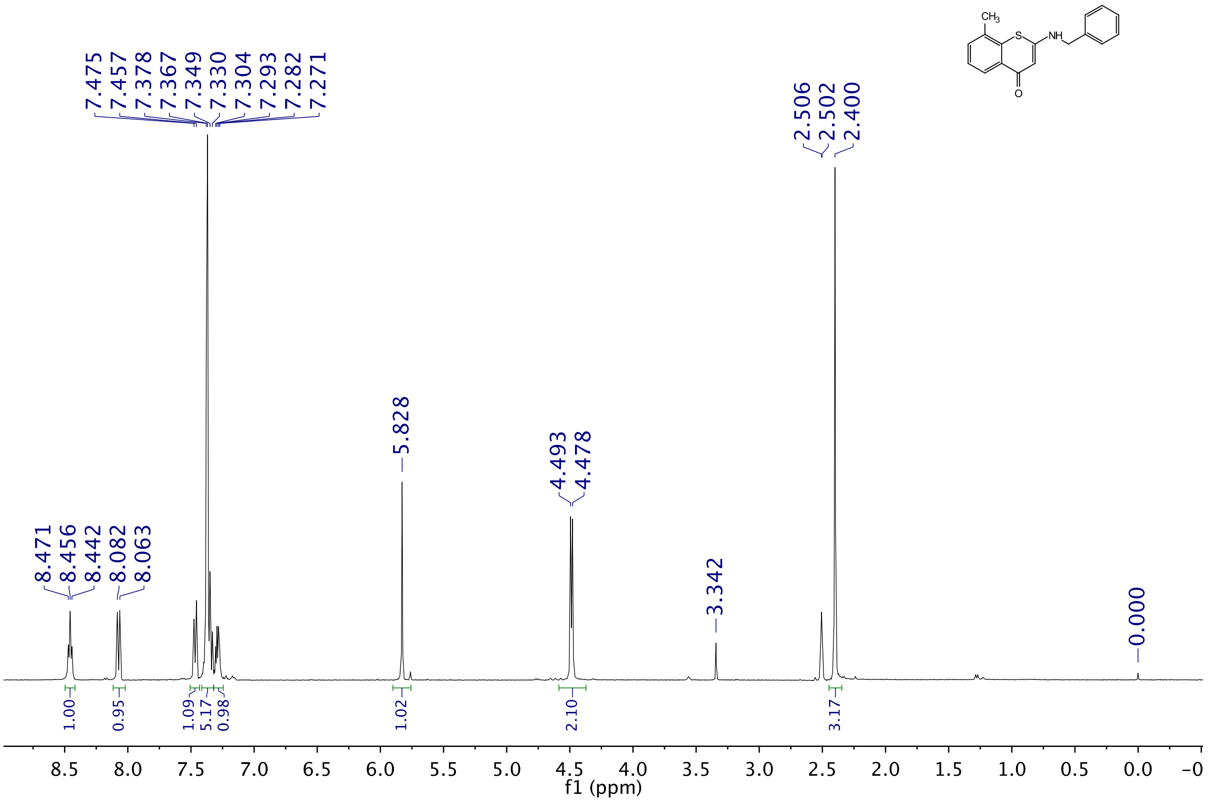

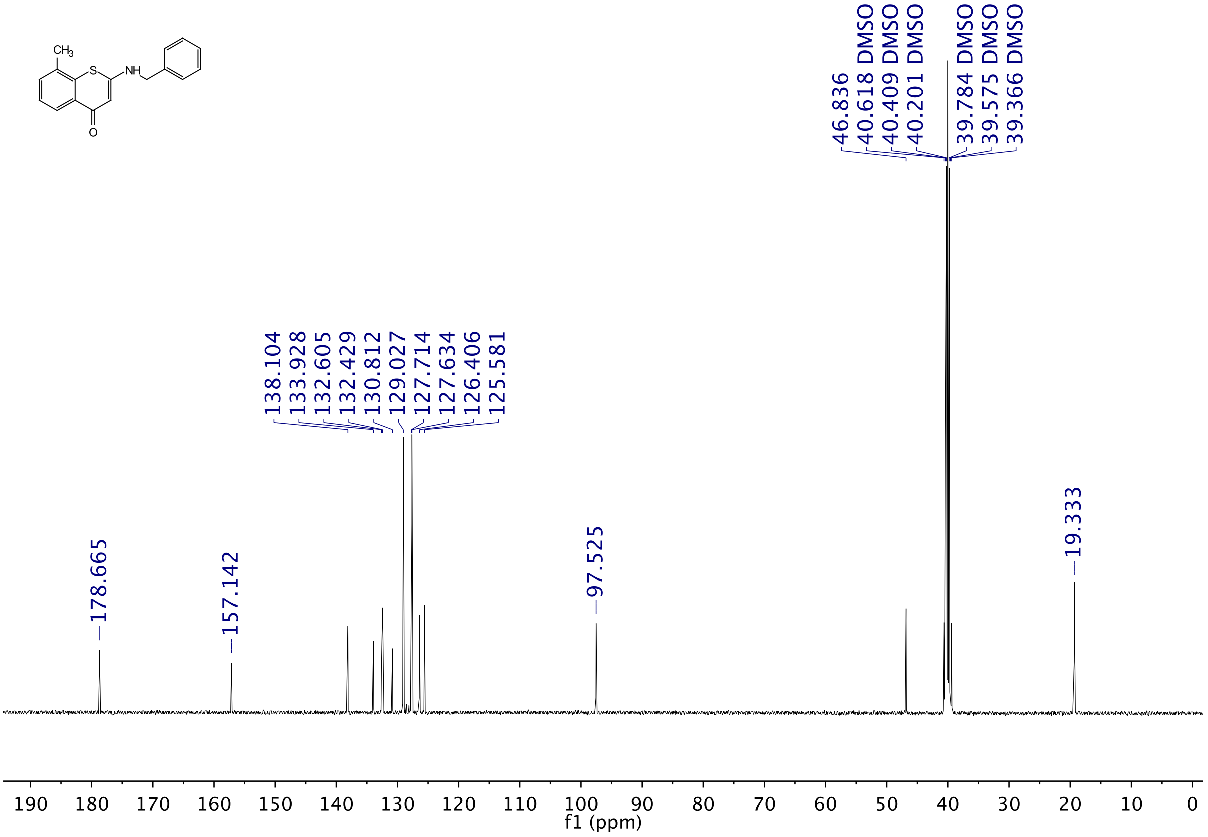

^{1}H and ^{13}C NMR spectra of 8-methyl-2-(propylamino)-4*H*-thiochromen-4-one **(4q)**

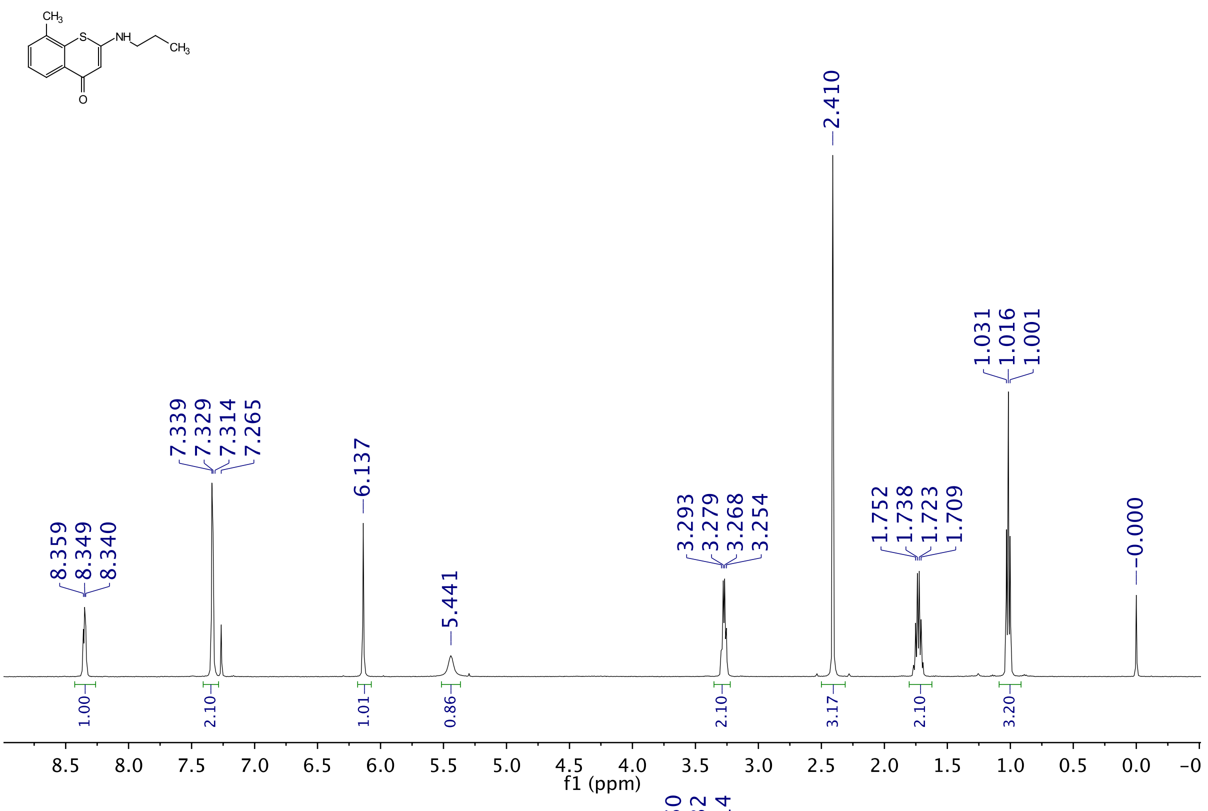

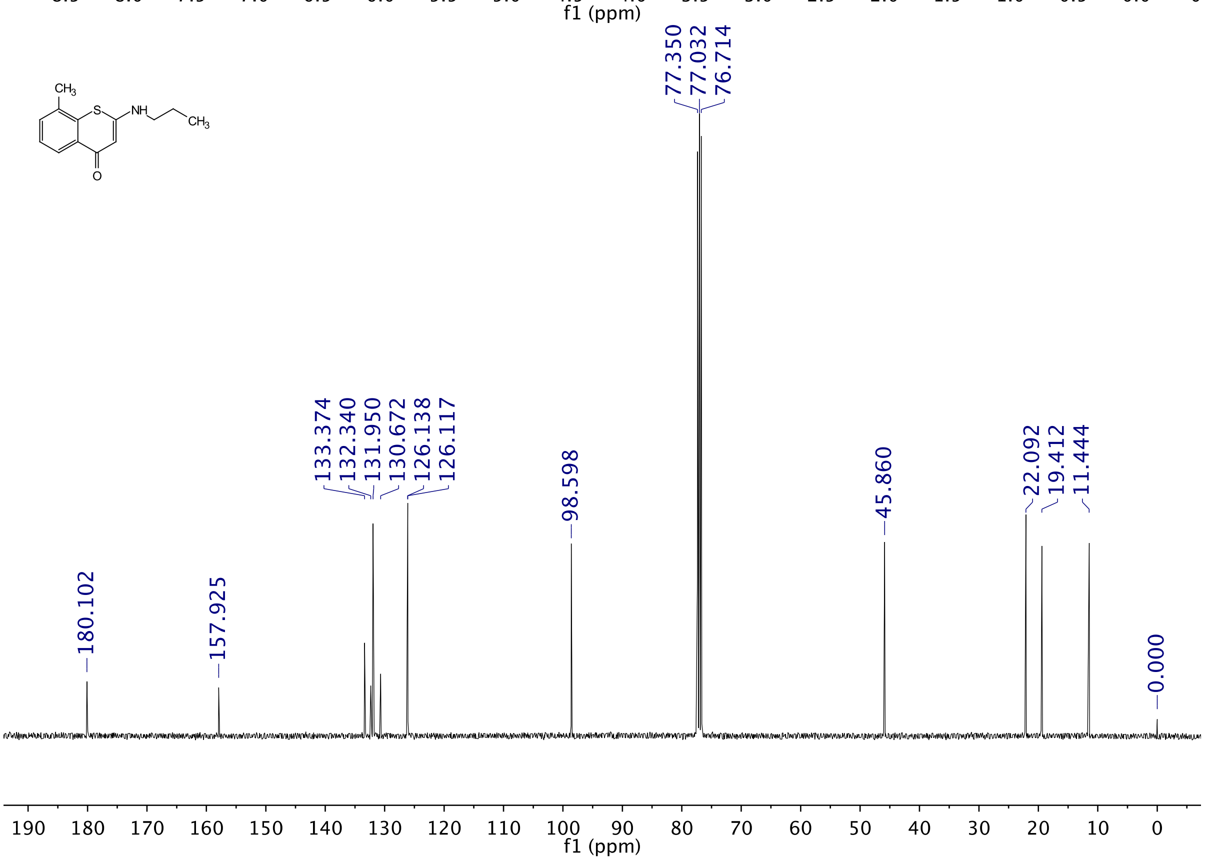

^{1}H and ^{13}C NMR spectra of 7-chloro-2-(phenylamino)-4*H*-thiochromen-4-one **(4r)**

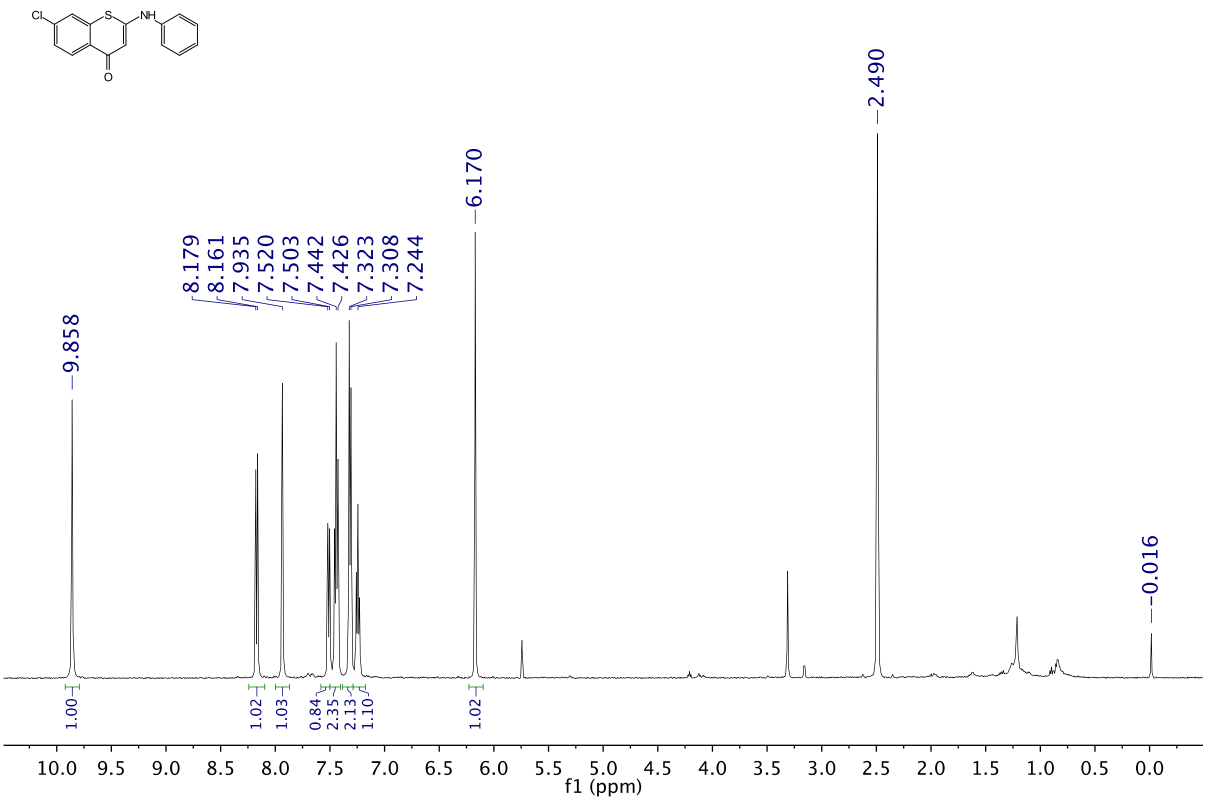

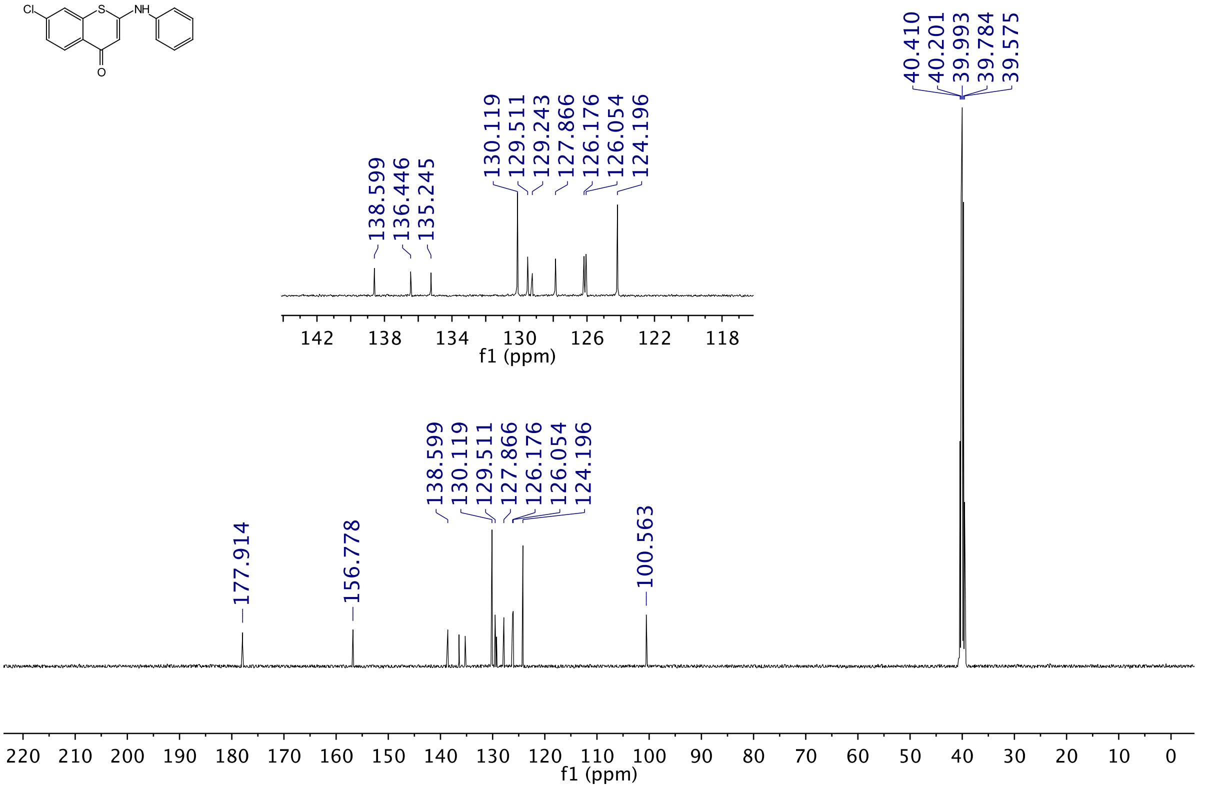

^{1}H and ^{13}C NMR spectra of 7-chloro-2-((3,4-dimethoxyphenyl)amino)-4*H*-thiochromen-4-one **(4s)**

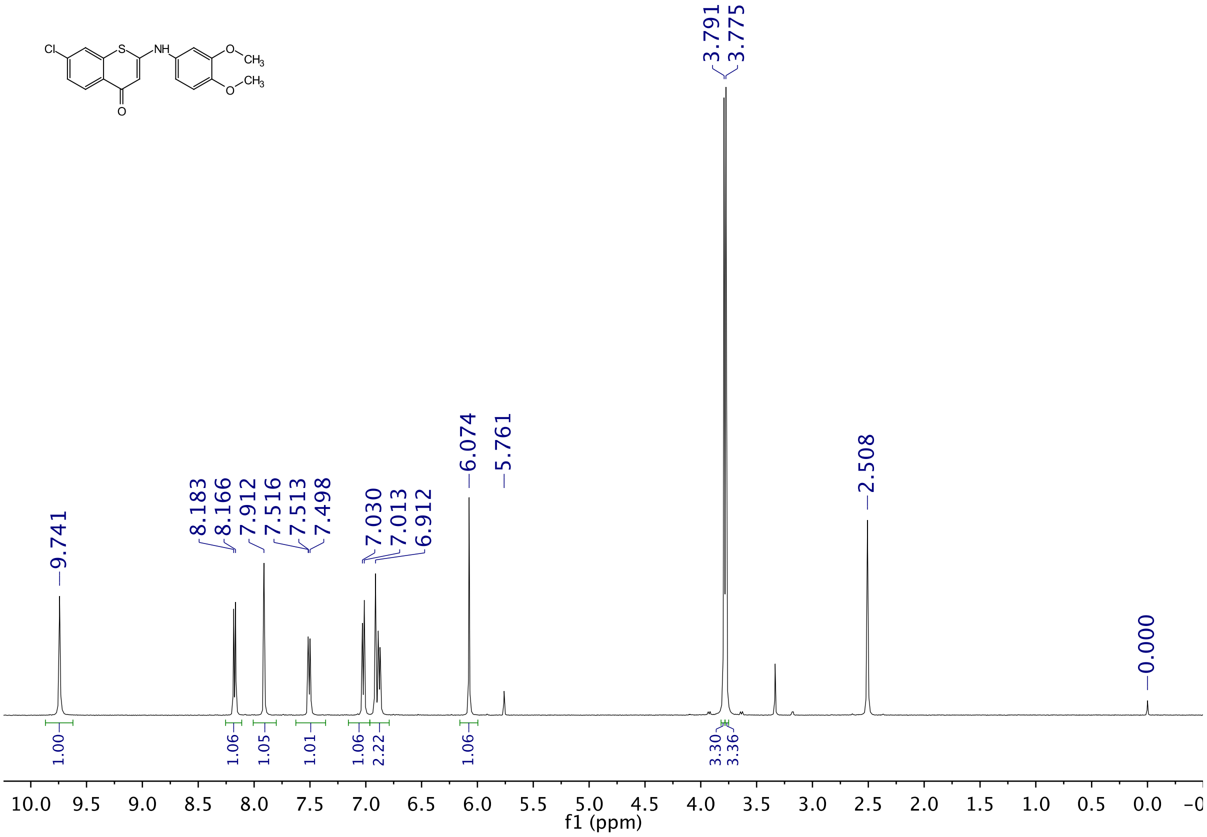

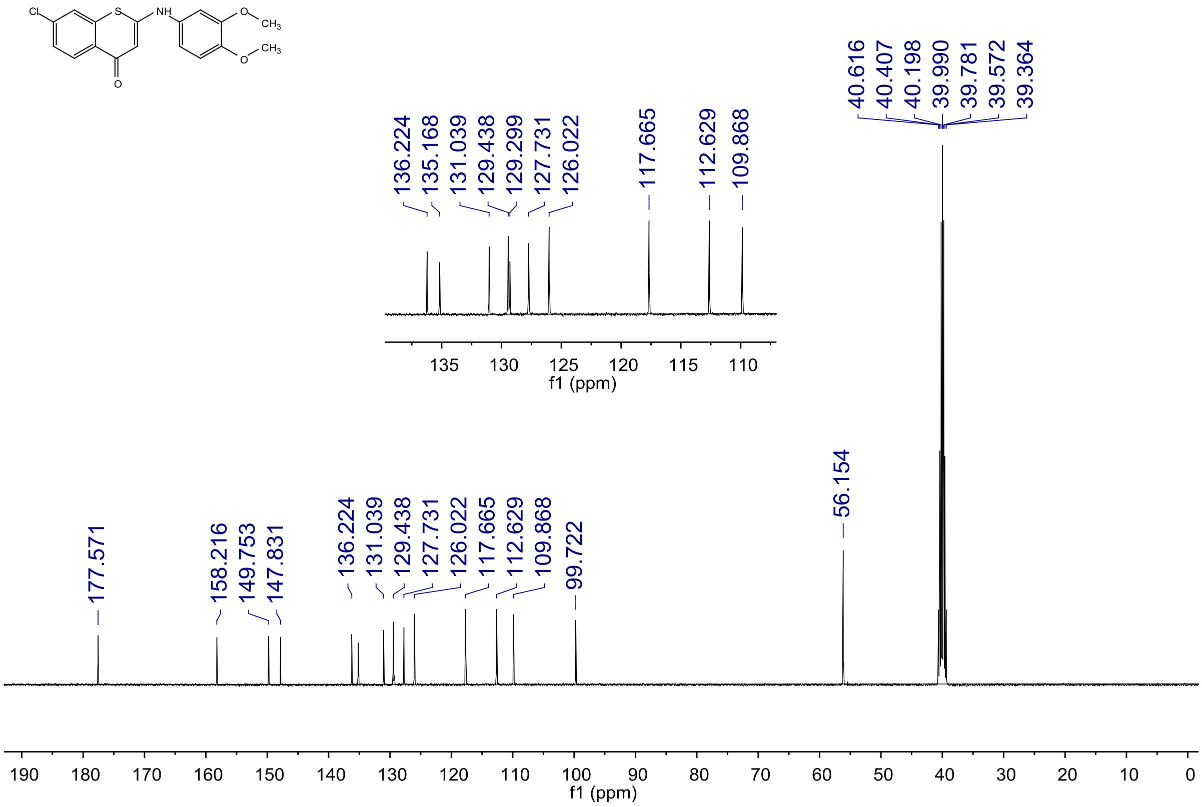

^{1}H and ^{13}C NMR spectra of 2-(4-benzylpiperazin-1-yl)-7-chloro-4*H*-thiochromen-4-one **(4t)**

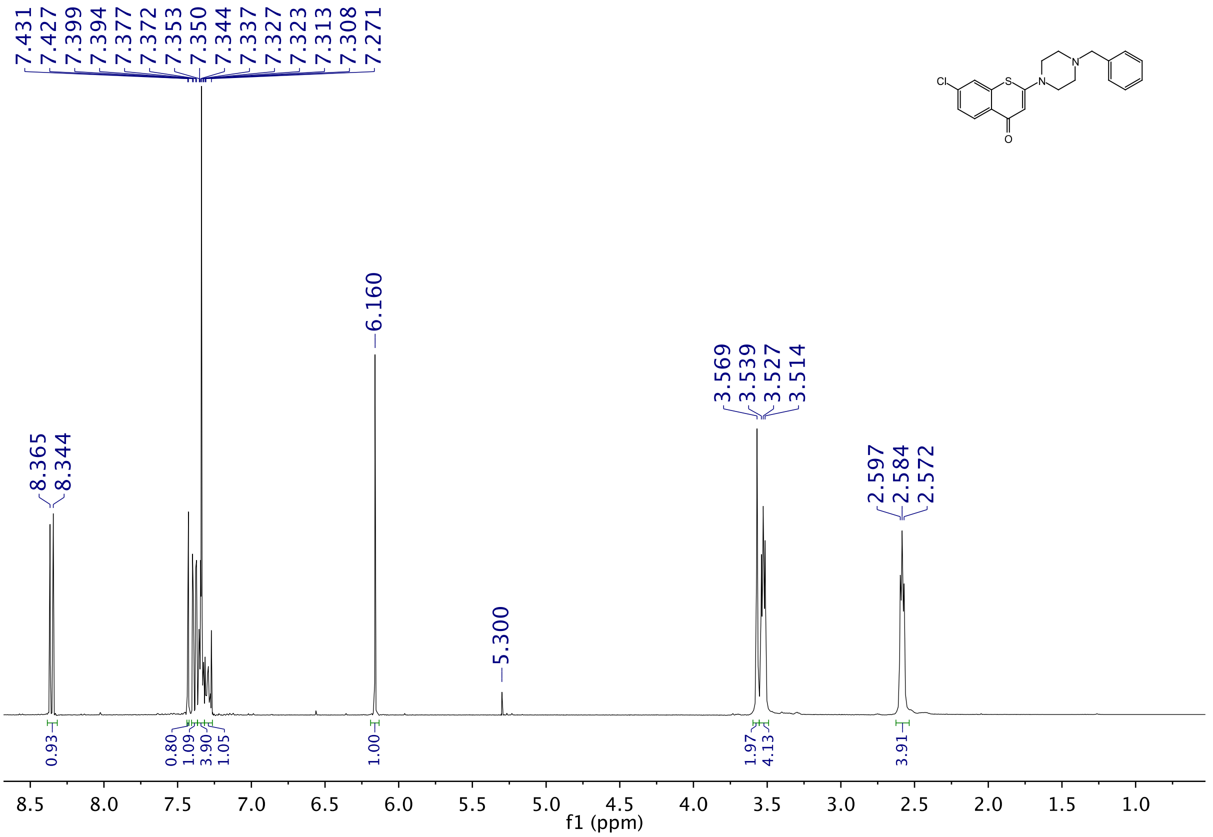

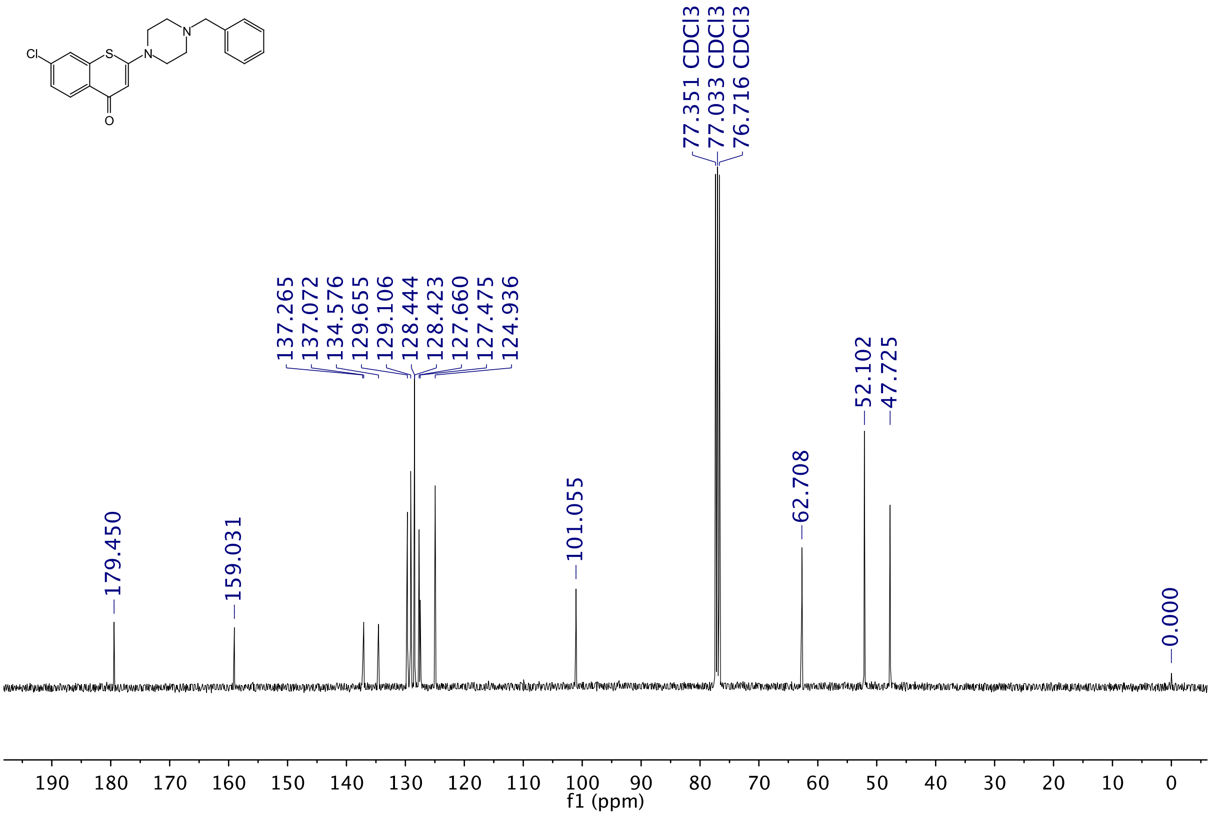

^{1}H and ^{13}C NMR spectra of 7-chloro-2-morpholino-4*H*-thiochromen-4-one **(4u)**

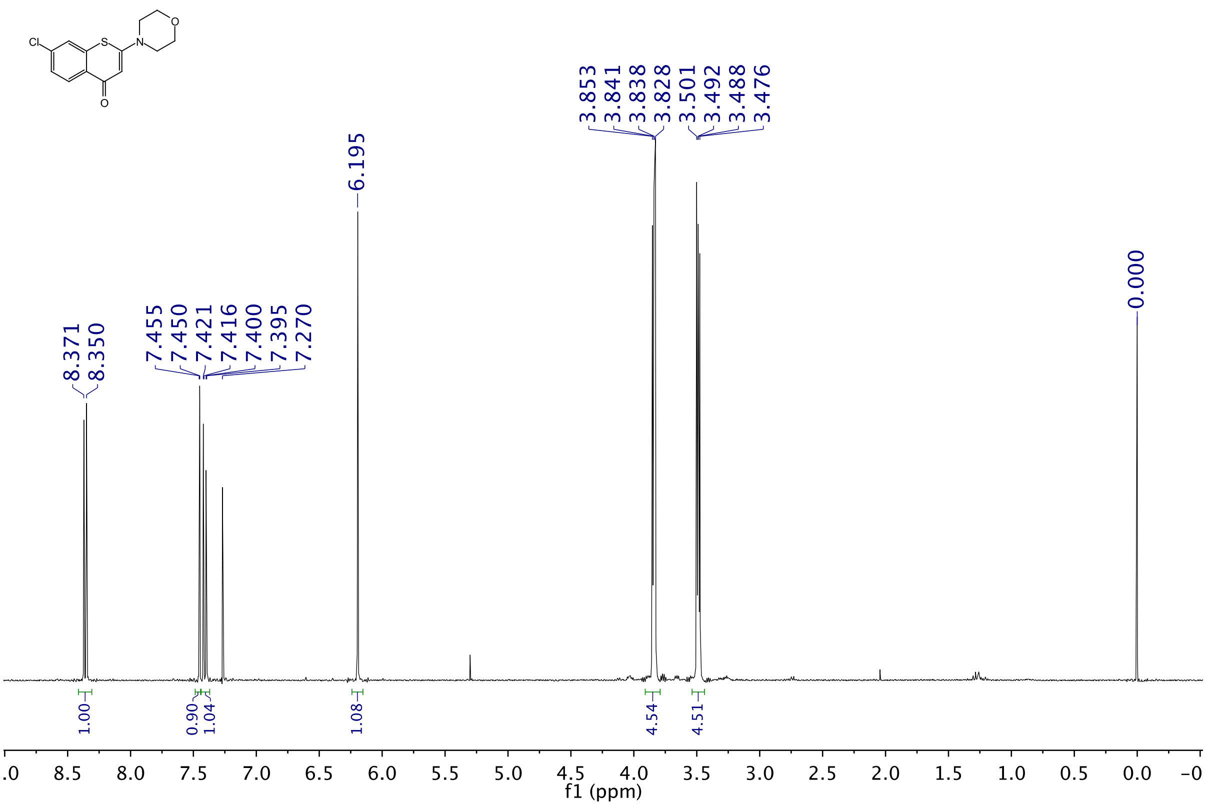

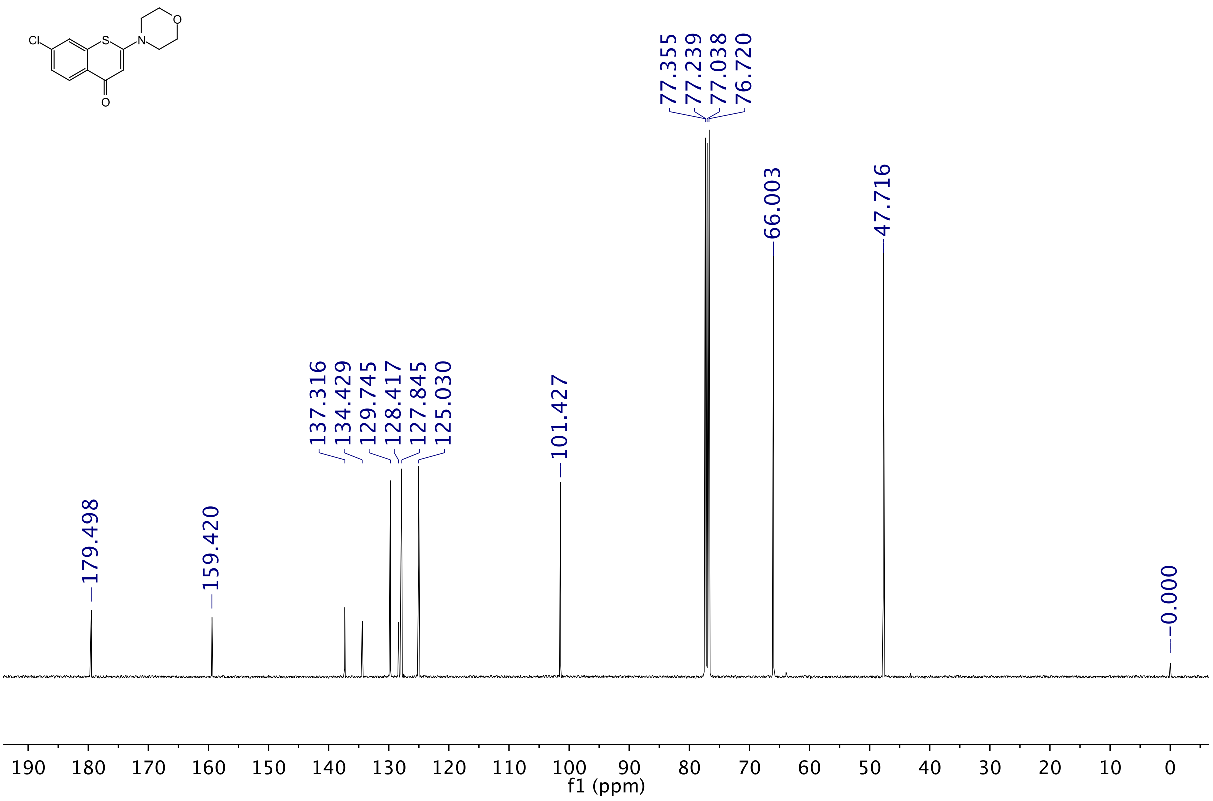

Supplement: File 1 — General methods, general procedures, characterization data and copies of 1H and 13C NMR spectra of 1a–h, 2a–h, 3a and 4a–u. [file Beilstein_J_Org_Chem-15-703-s001.pdf]
